# Aldehydes and ketones influence reactivity and selectivity in nickel-catalysed Suzuki–Miyaura reactions†‡§

**DOI:** 10.1039/c9sc05444h

**Published:** 2020-01-06

**Authors:** Alasdair K. Cooper, David K. Leonard, Sonia Bajo, Paul M. Burton, David J. Nelson

**Affiliations:** WestCHEM Department of Pure and Applied Chemistry, University of Strathclyde 295 Cathedral Street Glasgow G1 1XL Scotland UK david.nelson@strath.ac.uk; Syngenta, Jealott's Hill International Research Centre Bracknell Berkshire RG42 6EY UK

## Abstract

The energetically-favorable coordination of aldehydes and ketones – but not esters or amides – to Ni^0^ during Suzuki–Miyaura reactions can lead either to exquisite selectivity and enhanced reactivity, or to inhibition of the reaction. Aryl halides where the C–X bond is connected to the same π-system as an aldehyde or ketone undergo unexpectedly rapid oxidative addition to [Ni(COD)(dppf)] (**1**), and are selectively cross-coupled during competition reactions. When aldehydes and ketones are present in the form of exogenous additives, the cross-coupling reaction is inhibited to an extent that depends on the strength of the coordination of the pendant carbonyl group to Ni^0^. This work advances our understanding of how common functional groups interact with Ni^0^ catalysts and how these interactions affect workhorse catalytic reactions in academia and industry.

## Abstract 2

Aldehydes and ketones can have beneficial or detrimental effects on nickel-catalysed reactions. When present on the aryl halide, excellent site-selectivity can be achieved; when present as additives, they inhibit the reaction.

## Graphical abstract

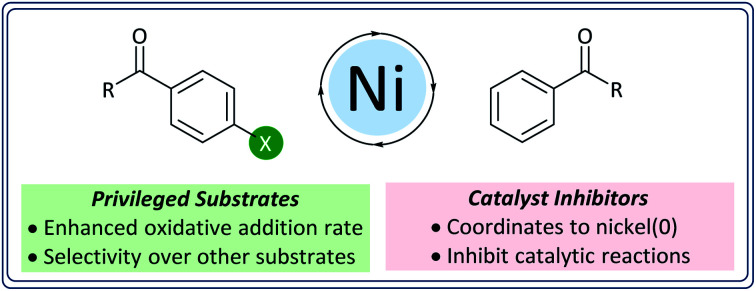

## Introduction

Nickel catalysis has the potential to replace palladium catalysis in some reactions and to enable new reactivity that can be exploited in organic synthesis.^1^ These reactions include tandem photocatalysis/cross-coupling,^2,3^ reductive cross-electrophile coupling,^4^ and the cross-coupling of phenol derivatives,^5^ aryl fluorides,^6,7^ and amides.^8^ Several issues remain to be resolved before the full potential and impact of nickel catalysis can be realised. We must understand how nickel interacts with the functional groups that are present in target molecules in the fine chemicals, agrochemicals, and pharmaceutical industries to understand the scope and limitations of existing methods and the opportunities and challenges to consider when developing new ones. The underlying reaction mechanisms in nickel catalysis, and how these depend on substrate and ligand structure, remain relatively poorly understood compared to palladium catalysis, and so nickel-catalysed reactions are often treated as a ‘black box’. Correspondingly, reaction design and optimisation rely heavily on empirical observations.

**Fig. 1:**
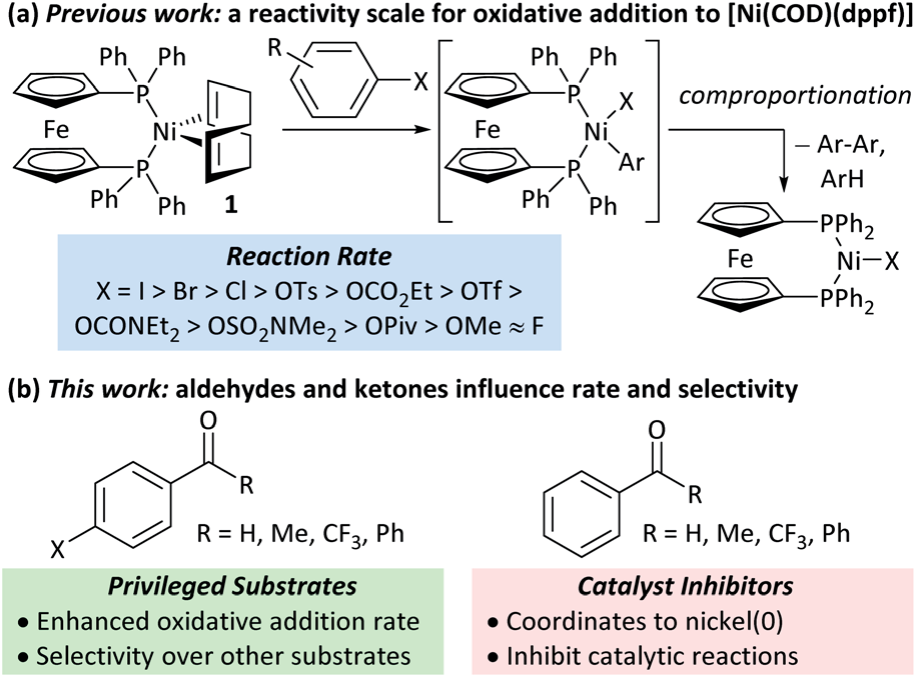
(a) Previous work. (b) This work.

The mechanisms of nickel-catalysed cross-coupling reactions show a complex dependence on ligand and/or substrate. The mechanistic landscape is further complicated by the often-ambiguous role of nickel(i) species in catalysis.^9–12^ [Ni(NHC)_2_] and [Ni(PR_3_)_4_] complexes react with aryl halides by oxidative addition or halide abstraction, depending on the ligand and substrate structure.^13–17^ [Ni(COD)(dppf)] (**1**)^18^ undergoes oxidative addition to aryl halides, followed by rapid comproportionation to form [NiX(dppf)] (Fig. 1(a));^19^ we have established the order of reactivity of a series of aryl (pseudo) halides. It was noted during our previous studies that aryl halides that had aldehyde or ketone substituents underwent unexpectedly fast oxidative addition. Further investigations have revealed that aldehyde and ketone functional groups can act either as directing groups for selective synthesis or as inhibitors of catalytic reactions (Fig. 1(b)). Our initial empirical study showed that various coordinating groups elicit these effects, and that they are considerably more marked for nickel than for palladium.^20^ Here, we examine the specific case of aldehydes and ketones in depth using a range of experimental and computational techniques.

## Results and discussion

### Kinetic studies of oxidative addition

Aldehyde and ketone-substituted aryl chlorides undergo surprisingly rapid oxidative addition, compared to the oxidative addition rates for a set of other substrates. The consumption of **1** in the presence of an excess of aryl halide was monitored using ^31^P NMR spectroscopy and exhibited pseudo-first order behaviour; rate data are recorded in Table 1. The ultimate nickel-containing products are [NiX(dppf)] complexes, as confirmed by EPR and ^1^H NMR spectroscopies.^21^ In contrast to cross-coupling reactions (*vide infra*) where transmetalation and reductive elimination can take place, the only pathway available to the arylnickel(ii) halide intermediates formed during these kinetic experiments is comproportionation with **1** to form Ni^I^ products.

**Fig. 2:**
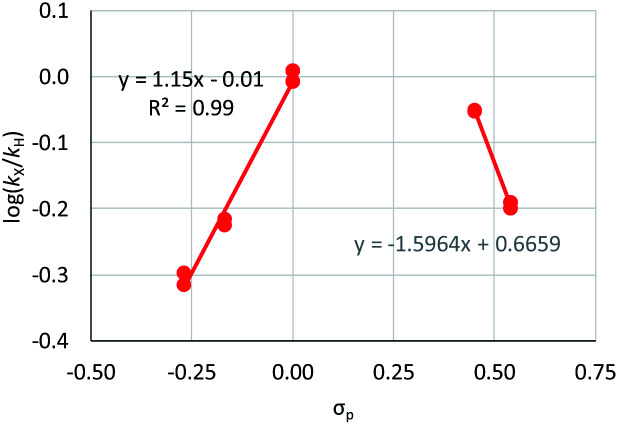
Hammett plot for oxidative addition of aryl chlorides to **1** from reactions at 50 °C in benzene-*d*_6_.^19^

**Table 1:** Psuedo-first order and relative rate constants for the reactions between **1** and substrates **2–6**. All rate constants are the average of two measurements. [nd] = not determined

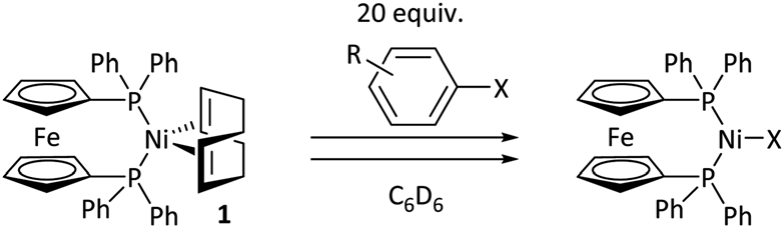			
Substrate	Oxidative addition rate constants		
	*k*_obs_ (20 °C)	*k*_obs_ (50 °C)	*k*_rel_
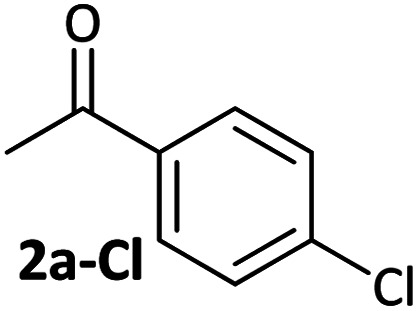	2.5(3) × 10^−4^ s^−1^	[nd]	0.32
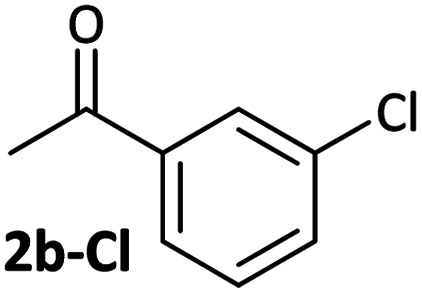	4.2(3) × 10^−4^ s^−1^	[nd]	0.54
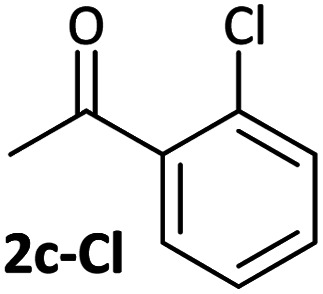	3.0(2) × 10^−4^ s^−1^	[nd]	0.38
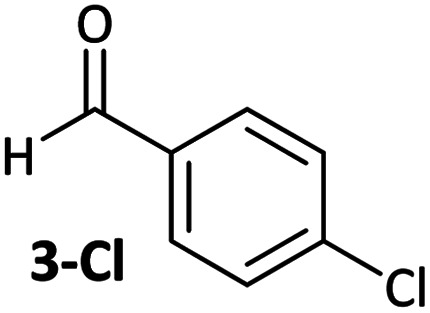	1.41(1) × 10^−4^ s^−1^	[nd]	0.18
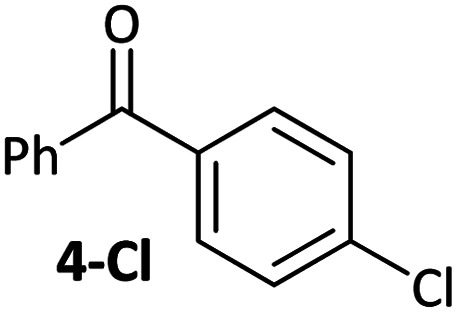	9.2(2) × 10^−5^ s^−1^	[nd]	0.12
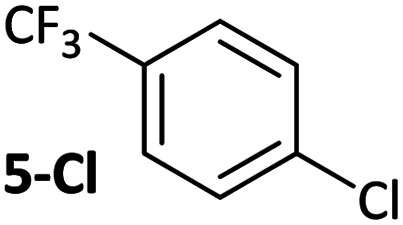	[nd]	2.39(3) × 10^−4^ s^−1^	0.007
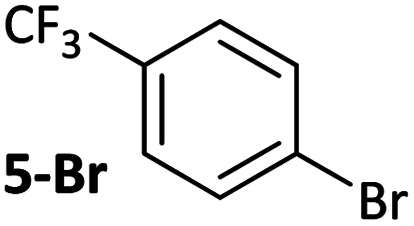	2.38(4) × 10^−5^ s^−1^	1.02(1) × 10^−3^ s^−1^	0.031
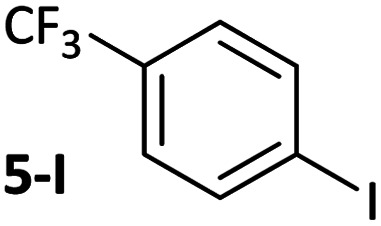	7.8(1) × 10^−4^ s^−1^	[nd]	1.0
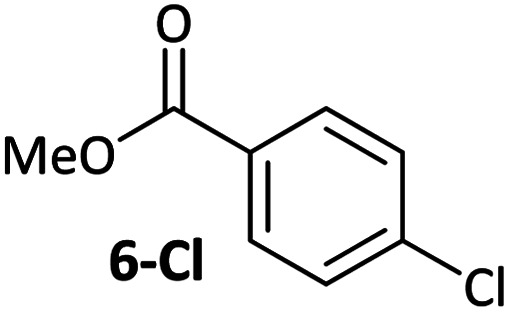	[nd]	1.85(4) × 10^−4^ s^−1^	0.005

Substrates **2-Cl** to **4-Cl** undergo oxidative addition much more rapidly than electron-deficient aryl chloride **5-Cl** or bromide **5-Br**. These large rate differences cannot be attributed simply to inductive or mesomeric electronic effects; Hammett *σ*_p_ is 0.4–0.5 for ketones, aldehydes and esters and 0.54 for trifluoromethyl.^22^ The Hammett plot for oxidative addition of aryl chlorides to **1** is shown in Fig. 2.^19^

Ligand exchange, where COD is replaced with the aryl halide, and the oxidative addition event that follows cannot be deconvoluted; we propose that the favourable coordination of aldehydes and ketones to Ni^0^ (*vide infra*) improves the equilibrium position of the ligand exchange (COD *versus* aryl halide), leading to a reaction that is more rapid overall. Consistent with this, signals that are tentatively assigned to an η^2^(CO) complex are observed during the oxidative addition reactions of **3-Cl**; various complexes of this type are known,^23–30^ and have been implicated in nickel-mediated reactions.^31^ The observed signals in the ^31^P{^1^H} NMR spectrum are consistent with a square planar complex (two doublets with ^2^*J*_PP_ of 31 Hz). This geometry is a result of electron donation from the bisphosphine ligand into the 3d(*x*^2^–*y*^2^) orbital, which is also engaged in d to π* back-bonding.^32,33^ The measured *K*_eq_ for the displacement of COD from **1** using benzaldehyde (>20), benzophenone (0.65), and acetophenone (0.02) are sufficiently large that under catalytic conditions – *i.e.* in the presence of a large excess (*ca.* 10–100 equiv.) of each cross-coupling partner – that this coordination would be expected to occur to the extent that between 2 and 100% of the Ni^0^ present would be coordinated to a carbonyl group. The lack of a similar effect for esters (*e.g.***6-Cl**) is attributed to n to 
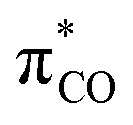
 resonance effects between the oxygen lone pair and the ester carbonyl group.

### Cross-coupling selectivity

**Fig. 3:**
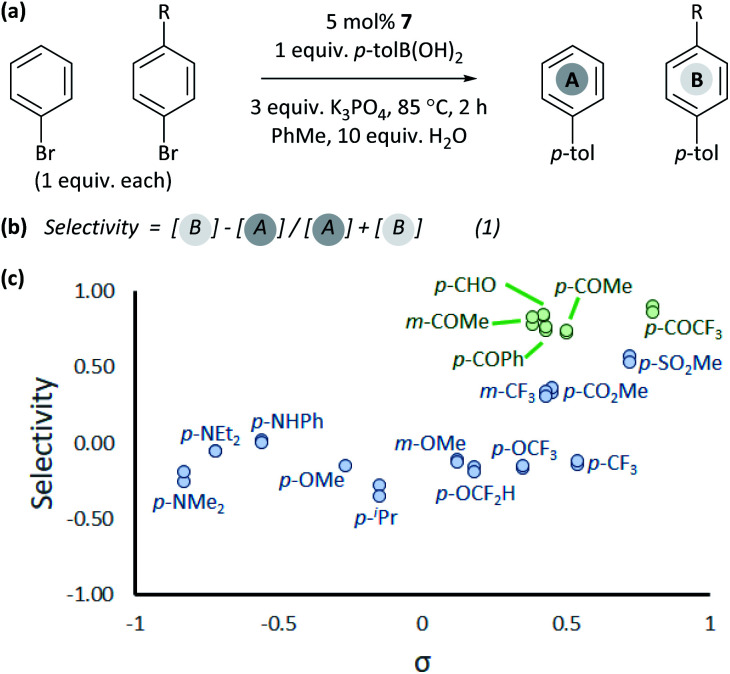
Competition experiments: (a) reaction conditions; (b) quantification of selectivity; and (c) reactions in toluene with 10 equiv. water. Each individual competition experiment is plotted as a separate point.

The coordination of aldehydes and ketones to Ni^0^ can be leveraged to achieve selective cross-coupling. Optimised cross-coupling conditions were developed for the prototypical cross-coupling of **2a-Br** with *p*-tolylboronic acid, catalysed by well-defined [NiCl(*o*-tol)(dppf)] pre-catalyst **7**,^34^ using factorial experimental design.§ To dissect the contributions of electronic and coordination effects, experiments were performed in which bromobenzene and a functionalized aryl bromide competed for a limiting amount of boronic acid (Fig. 3(a)).**Reaction outcomes were analysed using calibrated GC-FID analyses. In some competition experiments, total yield sometimes slightly exceeded 100% which is likely to be due to errors in weighing out exactly one equivalent of boronic acid. Data were interpreted by quantifying selectivity using eqn (1) (Fig. 3(b)); this has values between −1 and +1 which represent complete selectivity for bromobenzene and functionalised aryl bromide, respectively. The reaction selectivity was plotted *versus σ* (Fig. 3(c)).^35^ These data show that selectivity is typically insensitive to the electronic properties of the aryl halide, but significant selectivity is achieved when the aryl halide has a ketone or aldehyde functional group.^36^ This selectivity trend is observed for both *meta*- and *para*-substituted aryl bromides. These data confirm that aldehyde and ketone substituents can be used to induce selective cross-coupling at one substrate present within the reaction.

### Computational modelling of ring walking and oxidative addition

**Fig. 4:**
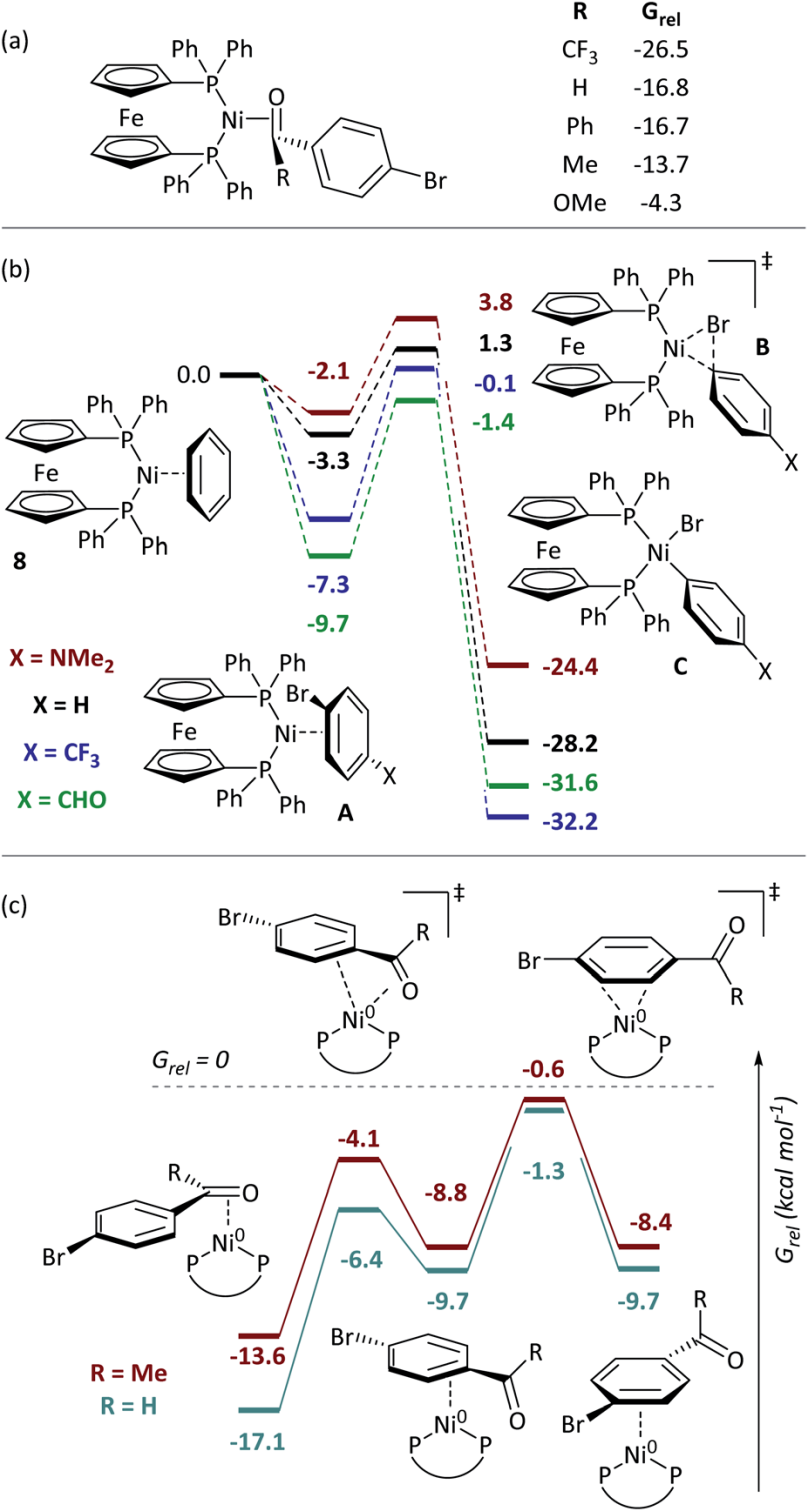
(a) Coordination to aldehydes, ketones, and esters. (b) Free energy profile for oxidative addition. (c) Calculations on the ring-walking process. All energies are free energies in toluene solution and are quoted in kcal mol^−1^.

DFT calculations give further insights into these reactions.††††Calculations were carried out using Gaussian09 Rev. D01.^54^ Geometry optimisations were carried out without symmetry constraints using the B3LYP functional with Grimme's D3 dispersion corrections, the LANL2DZ(dp) basis set on Br and I, the LANL2TZ(f) basis set on Ni and Fe, and the 6-31G(d) basis set on all other atoms. The nature of stationary points was confirmed using frequency calculations. Energies were refined using single point calculations in which the 6-31G(d) basis set was exchanged for 6-311+G(d,p). All calculations were carried out in toluene solvent using the SMD solvent model. [Ni(dppf)(η^2^-benzene)] (**8**) was assigned *G*_rel_ = 0. Consistent with experiment, aldehydes and ketones coordinate [Ni(dppf)] exergonically *via* the carbonyl group (Fig. 4(a)). Free energy profiles were calculated for the oxidative addition reactions of selected aryl bromides (Fig. 4(b));^37^ reactions proceed *via* transition state B to irreversibly form Ni^II^ products C. The aldehyde substrate can also form the η^2^(CO) complex.

Further calculations were undertaken to model the ring-walking^38–42^ step, using **2a-Br** and **3-Br** as substrates. In each case, three η^2^-complexes are linked by two transition states, leading from the carbonyl group to the halide site (Fig. 4(c)). The transition states for these ring-walking steps are all lower in energy than **8** and so this process is more energetically favourable than the dissociation of the aryl halide and coordination of a new aryl halide; once an aldehyde- or ketone-containing substrate coordinates the nickel centre *via* the carbonyl ligand, it will undergo oxidative addition after ring walking, rather than ligand exchange for an alternative substrate. Substrate **2c-Br** undergoes a similar ring-walking process, again with no intermediates or transition states that have *G*_rel_ > 0.§ These data are consistent with two key experimental observations: (i) the enhanced rate of oxidative addition, because η^2^(CO) coordination renders aldehyde- and ketone-containing aryl halides competitive ligands (*versus* COD); and (ii) selective cross-coupling, because aldehyde- and ketone-containing aryl halides are much better ligands for Ni^0^ than aryl halides without such strongly coordinating groups.

### Catalyst inhibition by aldehydes and ketones

**Fig. 5:**
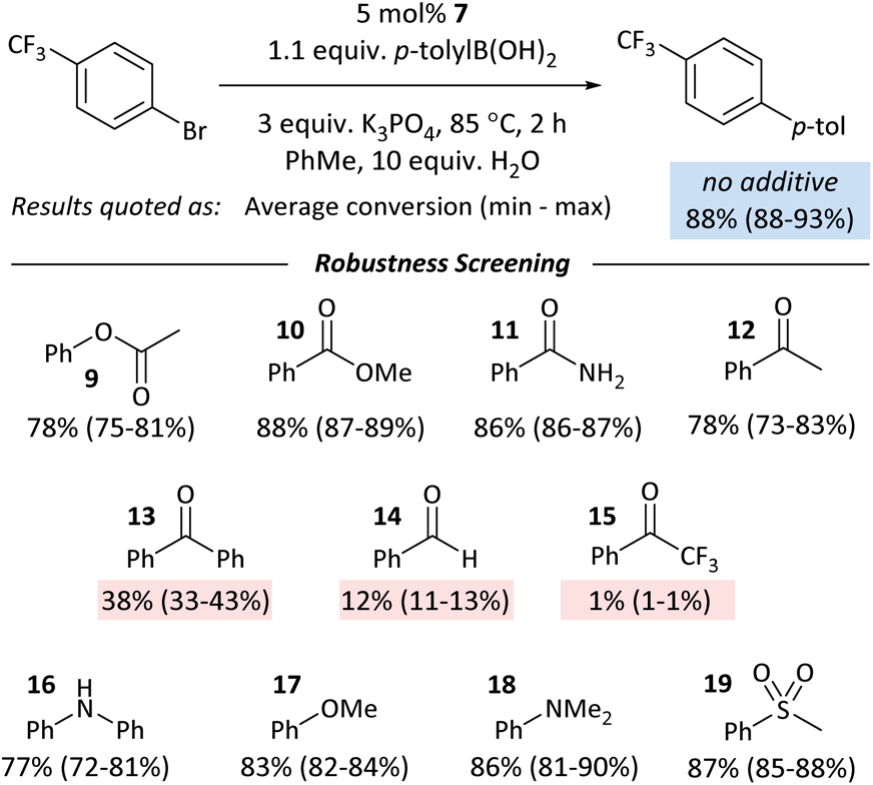
Results from the robustness screen.

The coordination of ketones and aldehydes to Ni^0^ might be expected to have a detrimental effect on the performance of cross-coupling reactions if this behaviour sequesters the active catalyst. A ‘robustness screen’^43,44^ was used, in which a model reaction was carried out in the presence of 1 equiv. of each of a series of additives (**9–19**).* This provides a rapid and quantitative measure of the effect of each additive. A palette of additives was examined (Fig. 5).

Most additives had little effect on the reaction conversion. Aldehyde and ketone additives – except for acetophenone (**12**) – had a significant and detrimental effect, with the reaction almost completely ceasing when 1 equiv. of 2,2,2-trifluoroacetophenone (**15**) was present. The degree of inhibition of the reaction is correlated to *K*_eq_ for the displacement of COD from **1** (*vide supra*); it may be necessary to protect aldehydes and ketones in some reactions to prevent their coordination to Ni^0^. If catalyst decomposition was responsible for the decrease in yield then we would expect that at the end of the reaction ≥5% of the additive would have been consumed;^45^ in the case of **13** and **14**, ≤ 2% of the additive is unaccounted for, while **15** hydrates in the presence of water, resulting in the absence of 12–15% of **15** at the end of the reaction. There is no correlation between the degree of inhibition and the amount of additive unaccounted for.

Control experiments where one equivalent of benzaldehyde was added to the cross-coupling of **4-Cl** and *p*-tolylboronic acid showed complete conversion to the cross-coupling product. Aldehyde and ketone additives therefore have less of an effect on the cross-coupling reactions of aldehyde- and ketone-containing aryl halides than they do on the cross-coupling of aryl halides that do not contain these functional groups.

The robustness screening data are consistent with coordination of an exogenous additive to the Ni^0^ catalyst inhibiting the reaction. No ring-walking is possible from such intermediates to a site for oxidative addition or other exergonic onward reaction.

### On the generality of these effects

**Fig. 6:**
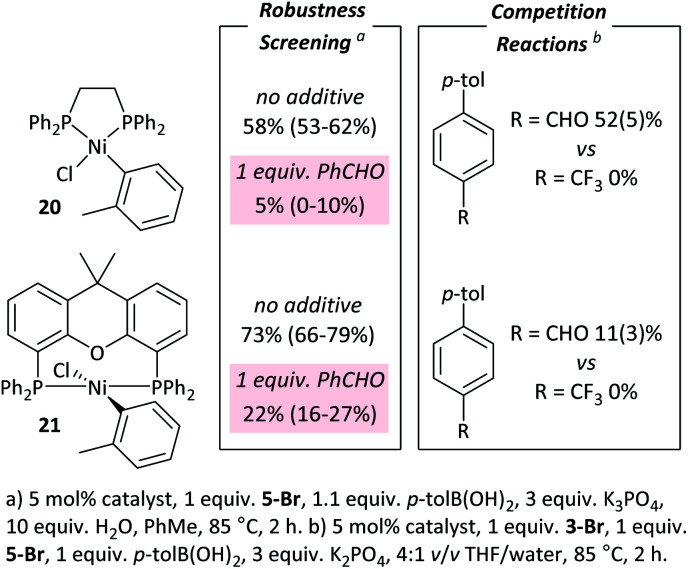
Results with dppe and Xantphos catalysts.

These observations are not limited to dppf-based (pre)catalysts. [NiCl(*o*-tol)(L)_*n*_] (L = dppe (**20**), Xantphos (**21**))^34^ were applied in: (i) competition reactions between **5-Br** and **3-Br**, in which **3-Br** reacts exclusively; and (ii) robustness screening reactions using **5-Br** as the substrate and benzaldehyde as the additive, showing significant reaction inhibition (Fig. 6).†† Complexes **20** and **21** perform significantly less well than **7** but may yield better results if further reaction optimisation was carried out. The results in Fig. 6 illustrate that the effect is general to nickel phosphine complexes, and not limited to dppf-based catalysts. Palladium, specifically [PdCl_2_(dppf)], shows a far reduced propensity to undergo selective cross-coupling reactions, but has far better functional group tolerance.^20^

### Opportunities for exploitation

**Fig. 7:**
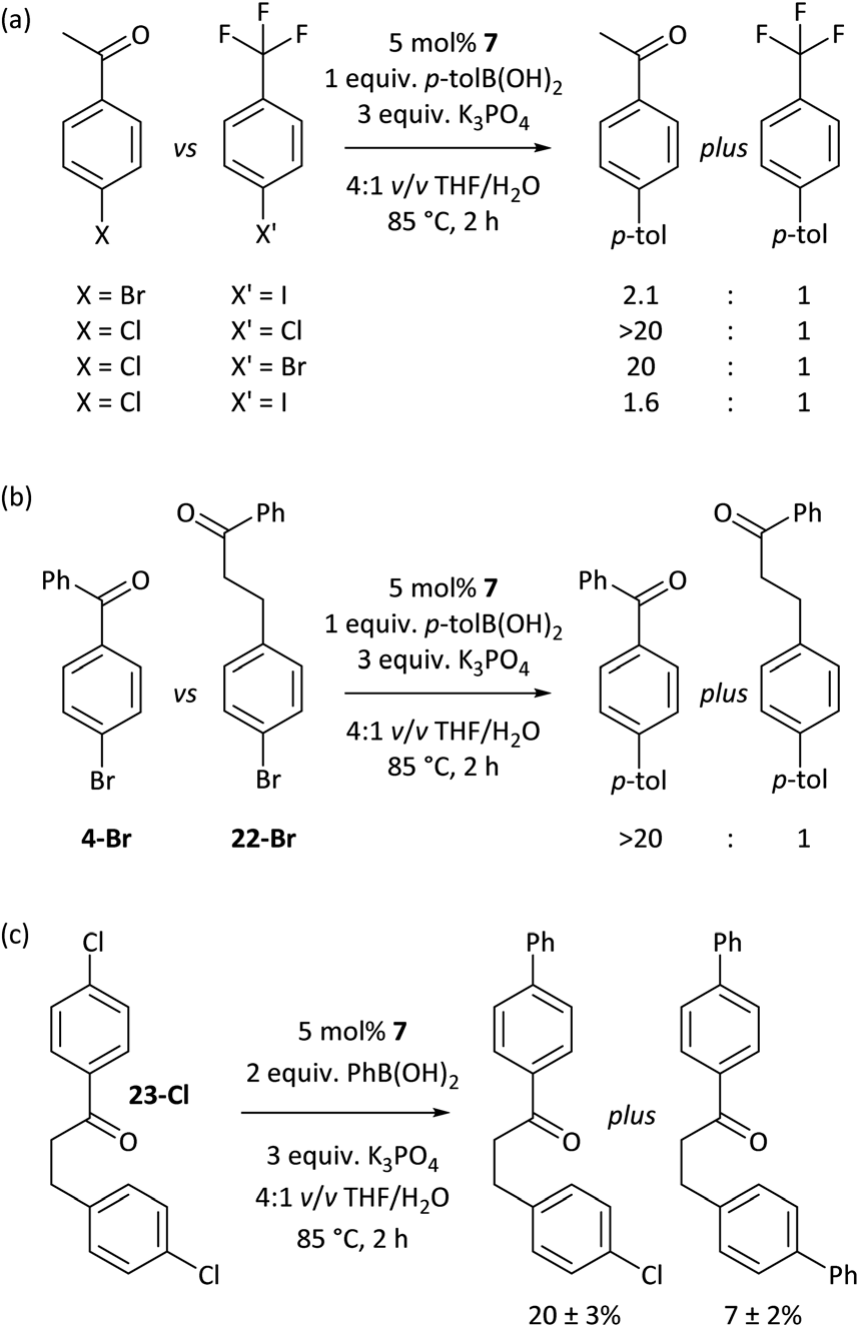
(a) Overriding the intrinsic reactivity differences between aryl halides. (b) Selectivity between different carbonyl-containing substrates. (c) Site-selectivity within a molecule, enforced by carbonyl coordination.

The carbonyl coordination effect was leveraged to achieve site selectivity in cross-coupling reactions, both intermolecularly and intramolecularly (Fig. 7). The aldehyde/ketone effect allows the normal reactivity order of aryl halides (I > Br > Cl)^19^ to be entirely overridden, to the extent that an aryl chloride (**2a-Cl**) undergoes selective coupling in the presence of an electron-deficient aryl bromide (**5-Br**) and even in the presence of an aryl iodide (**5-I**) (Fig. 7(a)). Reactions are selective for aldehydes over ketones, as judged from further competition experiments.§ Ketones that are not in conjugation with the aryl halide π-system are not selectively coupled (*e.g.* 22-Br) (Fig. 7(b)). Intramolecular competition experiments were carried out using **23-Cl**, which has two aryl chloride sites that are available for cross-coupling but only one is in conjugation with a ketone (Fig. 7(c)); reactions with **23-Cl** establish that selective cross-coupling occurs at the site that is in conjugation with the ketone. Intrinsic differences in the reactivities of aryl chlorides, bromides, and iodides have been leveraged in the past for selective cross-coupling reactions,^46^ but this work shows that the substitution pattern of the aryl halide can significantly change the order of reactivity, opening new avenues for creative organic synthesis using nickel-catalysed cross-coupling.

## Conclusions

This work establishes how aldehydes and ketones can have positive or negative effects on nickel-catalysed reactions, depending on their location. The formation of η^2^-complexes has been implicated in some nickel-catalysed reactions^30,31,39,47,48^ but this work shows that coordination effects can influence even simple and ubiquitous Suzuki–Miyaura reactions.

• Aryl halides with aldehyde and ketone functional groups undergo rapid oxidative addition because of favourable ligand exchange and ring-walking processes.

• Selectivity is achieved for aldehyde- or ketone-containing aryl halides over other aryl halide substrates because of this favourable coordination.

• Aldehydes and ketones that are present in cross-coupling reactions but are not in conjugation with the aryl halide act as inhibitors of catalysis.

• Aldehydes and ketone-containing aryl halides undergo successful cross-coupling.

**Fig. 8:**
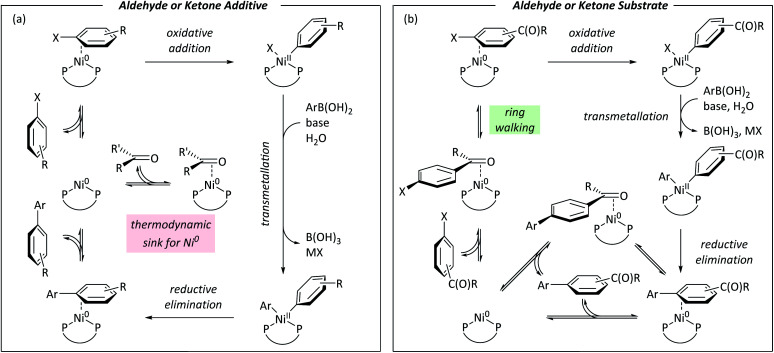
(a) Catalytic cycle for nickel-catalysed Suzuki–Miyaura reactions in the presence of exogenous aldehyde or ketone. (b) Catalytic cycle for nickel-catalysed Suzuki–Miyaura reactions of aldehyde- and ketone-containing aryl halides.

Fig. 8 summarises the conclusions of this study using Ni^0^/Ni^II^ cycles based on literature evidence from studies of nickel/dppf-catalysed reactions.^18,19,49–53^Fig. 8(a) shows a cycle for a reaction in which an aldehyde or ketone is present as an additive; a low energy η^2^(CO) complex sequesters the active catalyst and slows the reaction because the η^2^-complex with the aryl halide is higher in energy. Fig. 8(b) shows a cycle for an aryl halide with an aldehyde or ketone substituent.

We are currently pursuing further work within our laboratories to understand the effects of a wider range of functional groups on nickel-catalysed reactions and their fundamental steps using kinetic and mechanistic studies.

## Conflicts of interest

There are no conflicts to declare.

## Supplementary Material

SC-011-C9SC05444H-s001

SC-011-C9SC05444H-s002
