# Supplementary material for: Aldehydes and ketones influence reactivity and selectivity in nickel-catalysed Suzuki–Miyaura reactions
Source: Chem Sci. 2020 Jan 6;11(7):1905–11. doi: 10.1039/c9sc05444h (PMC8148322; doi:10.1039/c9sc05444h)
Supplement: SC-011-C9SC05444H-s002 [file SC-011-C9SC05444H-s002.pdf]

Electronic Supplementary Material (ESI) for Chemical Science.
This journal is © The Royal Society of Chemistry 2020

SUPPORTING INFORMATION FOR

Aldehydes and Ketones Influence Reactivity and Selectivity in Nickel-Catalysed Suzuki-Miyaura Reactions

Alasdair K. Cooper,[a] David K. Leonard,[a] Sonia Bajo,[a] Paul M. Burton,[b] and David J. Nelson*[a]

[a] WestCHEM Department of Pure and Applied Chemistry, University of Strathclyde, 295 Cathedral Street, Glasgow, UK, G1 1XL (david.nelson@strath.ac.uk)
[b] Syngenta, Jealott's Hill Research Centre, Bracknell, Berkshire, UK, RG42 6EX

CONTENTS

General experimental details … S2

Synthesis of organic compounds … S2

GC-FID calibration … S11

Design of experiments data for reaction optimisation … S12

Data from competitive cross-coupling reactions … S15

Data from robustness screening reactions … S18

Equilibrium constants for the binding of aldehydes and ketones to nickel(0) … S19

Kinetic data for oxidative addition to nickel(0) … S20

Energies from DFT calculations … S27

Coordinates from DFT calculations are supplied in a separate XYZ file

NMR Spectra … S36

References … S62

GENERAL EXPERIMENTAL DETAILS

General. The manipulations of air-sensitive nickel complexes and the execution of cross-coupling reactions were carried out under an atmosphere of argon or nitrogen using Schlenk techniques or an argon-filled Innovative Technology PureLab HE glovebox.

Reagents and Solvents. Most aryl halides were obtained from commercial sources and used as supplied. The syntheses of **22-Br** and **23-Br** are detailed subsequently. Complexes **1**, **7**, **20**, and **21** were prepared using literature methods.[1, 2] Anhydrous toluene and THF were obtained from an Innovative Technology PureSolv apparatus; regular Karl-Fisher analyses ensured that water content was always below 10 ppm. Anhydrous benzene-d_6 was obtained by drying over 4 Å molecular sieves that had been activated by heating under high vacuum. Distilled water was degassed by sparging with argon or nitrogen before use. Potassium phosphate was obtained commercially and dried overnight under vacuum at 50 °C before use and stored in a desiccator.

Analyses. NMR spectra were obtained using a Bruker AV3-400 instrument with a QNP probe or a liquid nitrogen Prodigy cryoprobe. Kinetic experiments were executed using a Bruker AVII-600 instrument equipped with a BBO-*z*-ATMA probe. ^{1}H NMR spectra are referenced to residual protonated solvent,[3] $^{13}C\{^{1}H\}$ NMR spectra are referenced to solvent signals,[3] ^{19}F NMR spectra are externally referenced to $CFCl_3$, and ^{31}P and $^{31}P\{^{1}H\}$ NMR spectra are externally referenced to H_3PO_4. GC-MS analyses were carried out using an Agilent 7890A gas chromatograph fitted with a RESTEK RXi-5Sil column (30 m x 0.32 mm I.D. x 0.25 µm) and an Agilent 5975C MSD running in EI mode. GC-FID analyses were carried out using an Agilent 7890A gas chromatograph fitted with an Agilent HP5 column (30 m x 0.25 mm I.D. x 0.25 µm).

SYNTHESIS OF ORGANIC COMPOUNDS

General Procedure A. To a microwave vial equipped with a stirrer bar, 4-tolylboronic acid (1.1 eq.), $[PdCl_2(dppf)]$ (1 – 5 mol%), K_3PO_4 (3 eq.) and, if solid, aryl halide (1 mmol, 1 eq.) were added. The vial was capped and purged/backfilled with N_2. Anhydrous toluene (2 mL) was added *via* oven-dried glass syringe. If the aryl halide was a liquid, it was added here. H_2O (10 eq.) was then added. The reaction was stirred for 2 hours at 85 °C. The reaction was then cooled to room temperature. The mixture was filtered through celite and analysed *via* GC-FID. The reaction was then analysed *via* TLC. The mixture was evaporated to dryness *in vacuo* and purified *via* flash column chromatography to furnish the product as a white solid.

4-methyl-4'-(trifluoromethyl)-1,1'-biphenyl

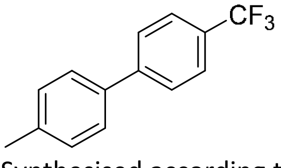

Synthesised according to the General Procedure A using 4-bromobenzotrifluoride (140 µL, 225.0 mg, 1 mmol), 4-tolylboronic acid (149.7 mg, 1.1 mmol), $[PdCl_2(dppf)]$ (36.5 mg, 5 mol %) and K_3PO_4 (634.2 mg, 3 mmol) in 2 mL toluene. The desired product was purified *via* flash column chromatography (eluting with hexane) to yield a white solid (163.7 mg, 69%).

^{1}H NMR (400 MHz, $CDCl_3$): δ_H 7.70 (s, 4H, 4 x ArH), 7.53 (d, 2H, 2 x ArH, J = 8.3 Hz), 7.31 (d, 2H, 2 x ArH, J = 7.9 Hz), 2.44 (s, 3H, CH_3). **^{13}C{^{1}H} NMR** (101 MHz, $CDCl_3$): δ_C 144.2, 137.7, 136.4, 129.2 (2C), 128.5 (q, $^{2}J_{C\text{-}F}$ = 32.5 Hz), 126.7 (2C), 126.6 (2C), 125.2 (q, 2C, $^{3}J_{C\text{-}F}$ = 3.7 Hz), 123.9 (q, $^{1}J_{C\text{-}F}$ = 271.9 Hz), 20.6. **^{19}F NMR** (376 MHz, $CDCl_3$): δ_F -62.4 (s, 3F, CF_3). **m/z** (GCMS EI): 236.1 (M^+). NMR data are consistent with the literature.[4]

methyl 4'-methyl-[1,1'-biphenyl]-4-carboxylate

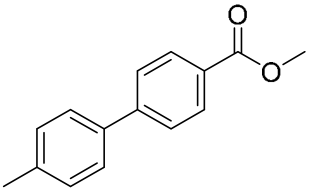

Synthesised according to the General Procedure A using methyl 4-bromobenzoate (214.6 mg, 1 mmol), 4-tolylboronic acid (150.1 mg, 1.1 mmol), [$PdCl_2$(dppf)] (36.4 mg, 5 mol %) and K_3PO_4 (634.0 mg, 3 mmol) in 2 mL toluene. The desired product was purified *via* flash column chromatography (eluting with 0 – 5 % EtOAc in hexane) to yield a white solid (209.1 mg, 92%).
^{1}H NMR (400 MHz, $CDCl_3$): δ_H 8.11 (d, 2H, 2 x ArH, *J* = 8.6 Hz), 7.67 (d, 2H, 2 x ArH, *J* = 8.5 Hz), 7.55 (d, 2H, 2 x ArH, *J* = 8.1 Hz), 7.31 (s, 2H, 2 x ArH), 3.96 (s, 3H, CO_2CH_3), 2.43 (s, 3H, CH_3). **^{13}C{^{1}H} NMR** (101 MHz, $CDCl_3$): δ_C 166.6, 145.1, 137.6, 136.6, 129.6 (2C), 129.2 (2C), 128.1, 126.6 (2C), 126.3 (2C), 51.6, 20.7. **ν_{max}** (neat): 3024, 2943, 2845, 1701, 1597, 1491 cm^{-1}. **m/z** (GC-MS EI) m/z: 226.1 (M^+). NMR data are consistent with the literature.[5]

***N*,*N*,4'-trimethyl-[1,1'-biphenyl]-4-amine**

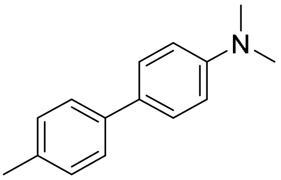

Synthesised according to the General Procedure A using 4-bromo-*N,N*-dimethylaniline (200.9 mg, 1 mmol), 4-tolylboronic acid (149.6 mg, 1.1 mmol), [$PdCl_2$(dppf)] (36.5 mg, 5 mol %) and K_3PO_4 (634.4 mg, 3 mmol) in 2 mL toluene. The desired product was purified *via* flash column chromatography (eluting with 0 – 10 % EtOAc in hexane) to yield a white solid (174.8 mg, 83 %).
^{1}H NMR (400 MHz, $CDCl_3$): δ_H 7.52 – 7.46 (m, 4H, 4 x ArH, *J* = 22.1 Hz), 7.23 (d, 2H, 2 x ArH, *J* = 8.0 Hz), 6.83 (d, 2H, 2 x ArH, *J* = 8.9 Hz), 3.02 (s, 6H, $N(CH_3)_2$), 2.40 (s, 3H, CH_3). **^{13}C{^{1}H} NMR** (101 MHz, $CDCl_3$): δ_C 149.3, 137.9, 135.1, 128.9 (2C), 127.0 (2C), 125.7 (2C), 112.4 (2C), 40.2 (2C), 20.5. **m/z** (GCMS EI): 211.1 (M^+). NMR data are consistent with the literature.[6]

4-methoxy-4'-methyl-1,1'-biphenyl

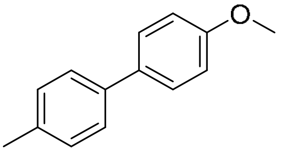

Synthesised according to the General Procedure A using 4-bromoanisole (125 µL, 186.8 mg, 1 mmol), 4-tolylboronic acid (149.3 mg, 1.1 mmol), [$PdCl_2$(dppf)] (36.7 mg, 5 mol %) and K_3PO_4 (634.4 mg, 3 mmol) in 2 mL toluene. The desired product was purified *via* flash column chromatography (eluting with 0 – 10 % EtOAc in hexane) to yield a white solid (99.6 mg, 50 %).
^{1}H NMR (400 MHz, $CDCl_3$): δ_H 7.53 (d, 2H, 2 x ArH, *J* = 9.0 Hz), 7.47 (d, 2H, 2 x ArH, *J* = 8.3 Hz), 7.25 (d, 2H, 2 x ArH, *J* = 7.8 Hz), 6.99 (d, 2H, 2 x ArH, *J* = 8.8 Hz), 3.88 (s, 3H, OCH_3), 2.41 (s, 3H, CH_3). **^{13}C{^{1}H} NMR** (101 MHz, $CDCl_3$): δ 158.4, 137.5, 135.9, 133.3, 128.9 (2C), 127.5 (2C), 126.1 (2C), 113.7 (2C), 54.9, 20.5. **m/z** (GCMS EI): 198.1 (M^+). NMR data are consistent with the literature.[4]

4'-methyl-[1,1'-biphenyl]-4-carbaldehyde

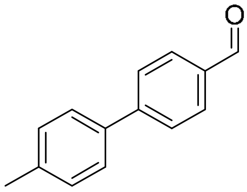

Synthesised according to the General Procedure A using 4-bromobenzaldehyde (186.2 mg, 1 mmol), 4-tolylboronic acid (150.1 mg, 1.1 mmol), [$PdCl_2$(dppf)] (37.0 mg, 5 mol %) and K_3PO_4 (634.4 mg, 3 mmol) in 2 mL toluene. The desired product was purified *via* flash column chromatography (eluting with 0 – 5 % EtOAc in hexane) to yield a white solid (115.2 mg, 59 %).
^{1}H NMR (400 MHz, $CDCl_3$): δ_H 10.08 (s, 1H, C(O)H), 7.96 (d, 2H, 2 x ArH, *J* = 8.6 Hz), 7.77 (d, 2H, 2 x ArH, *J* = 8.3 Hz), 7.57 (d, 2H, 2 x ArH, *J* = 8.1 Hz), 7.32 (d, 2H, 2 x ArH, *J* = 7.9 Hz), 2.45 (s, 3H, CH_3). **^{13}C{^{1}H} NMR** (101 MHz, $CDCl_3$): δ_C 191.4, 146.7, 138.0, 136.3, 134.5, 129.8 (2C), 129.3 (2C), 126.9 (2C), 126.7 (2C), 20.7. ***ν_{max}*** (neat): 3022, 2845, 1694, 1597, 1493 cm^{-1}. **m/z** (GCMS EI): 196.1 (M^+). NMR data are consistent with the literature.[7]

1-(4'-methyl-[1,1'-biphenyl]-4-yl)ethan-1-one

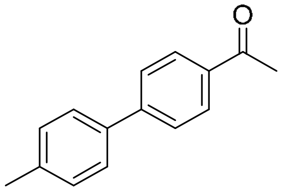

Synthesised according to the General Procedure A using 4'-bromoacetophenone (201.4 mg, 1 mmol), 4-tolylboronic acid (150.2 mg, 1.1 mmol), [$PdCl_2$(dppf)] (36.4 mg, 5 mol %) and K_3PO_4 (634.7 mg, 3 mmol) in 2 mL toluene. The desired product was purified *via* flash column chromatography (eluting with 0 – 5 % EtOAc in hexane) to yield a white solid (160.8 mg, 77 %).
^{1}H NMR (400 MHz, $CDCl_3$): δ_H 8.05 (d, 2H, 2 x ArH, *J* = 8.7 Hz), 7.70 (d, 2H, 2 x ArH, *J* = 8.7 Hz), 7.56 (d, 2H, 2 x ArH, *J* = 8.2 Hz), 7.31 (d, 2H, 2 x ArH, *J* = 8.2 Hz), 2.66 (s, 3H, C(O)CH_3), 2.44 (s, 3H, CH_3). **^{13}C{^{1}H} NMR** (101 MHz, $CDCl_3$): δ_C 197.3, 145.3, 137.8, 136.5, 135.1, 129.2 (2C), 128.4 (2C), 126.6 (2C), 126.5 (2C), 26.1, 20.7. ***ν_{max}*** (neat): 3026, 2928, 1674, 1597, 1522, 1491, 1420 cm^{-1}. **m/z** (GCMS EI): 210.1 (M^+). NMR data are consistent with the literature.[7]

(4'-methyl-[1,1'-biphenyl]-4-yl)(phenyl)methanone

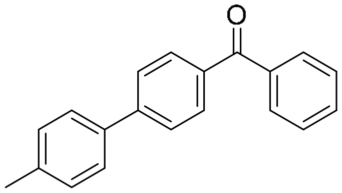

Synthesised according to the General Procedure A using 4-bromobenzophenone (261.1 mg, 1 mmol), 4-tolylboronic acid (150.3 mg, 1.1 mmol), [$PdCl_2$(dppf)] (36.7 mg, 5 mol %) and K_3PO_4 (634.9 mg, 3 mmol) in 2 mL toluene. The desired product was purified *via* flash column chromatography (eluting with 0 – 5 % EtOAc in hexane) to yield a white solid (136.3 mg, 50 %).
^{1}H NMR (400 MHz, $CDCl_3$): δ_H 7.91 (d, 2H, 2 x ArH, *J* = 8.5 Hz), 7.86 (d, 2H, 2 x ArH, *J* = 7.1 Hz), 7.72 (d, 2H, 2 x ArH, *J* = 8.6 Hz), 7.63 (t, 1H, 1 x ArH, *J* = 7.3 Hz), 7.58 (d, 2H, 2 x ArH, *J* = 8.3 Hz), 7.53 (t, 2H, 2 x ArH, *J* = 7.8 Hz), 7.32 (d, 2H, 2 x ArH, *J* = 8.1 Hz), 2.45 (s, 3H, CH_3). **^{13}C{^{1}H} NMR** (101 MHz, $CDCl_3$): δ_C 195.9, 144.7, 137.7, 137.4, 136.6, 135.5, 131.8, 130.2 (2C), 129.5 (2C), 129.2 (2C), 127.8 (2C), 126.6 (2C), 126.2 (2C), 20.7. ***ν_{max}*** (neat): 3022, 2911, 2853, 1643, 1595, 1528, 1491, 1443 cm^{-1}. **m/z** (GCMS EI): 272.1 (M^+). NMR data are consistent with the literature.[8]

4-fluoro-4'-methyl-1,1'-biphenyl

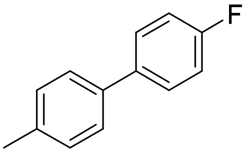

Synthesised according to the General Procedure A using 4-bromofluorobenzene (110 µL, 175.2 mg 1 mmol), 4-tolylboronic acid (135.6 mg, 1 mmol), [$PdCl_2$(dppf)] (7.5 mg, 1 mol %) and K_3PO_4 (633.3 mg, 3 mmol) in 2 mL toluene. The desired product was purified *via* flash column chromatography (eluting with hexane) to yield a white solid (160.1 mg, 86 %).

^{1}H NMR (400 MHz, $CDCl_3$): δ_H 7.57 – 7.53 (dd, 2H, 2 x ArH, $^3J_{H\text{-}H}$ = 8.8 Hz, $^4J_{H\text{-}F}$ = 5.3 Hz), 7.46 (d, 2H, 2 x ArH, J = 8.1 Hz), 7.27 (d, 2H, 2 x ArH, J = 8.0 Hz), 7.13 (t, 2H, 2 x ArH, J = 8.8 Hz), 2.42 (s, 3H, CH_3). **^{13}C{^{1}H} NMR** (101 MHz, $CDCl_3$): δ_C 161.8 (d, $^1J_{C\text{-}F}$ = 245.6 Hz), 136.9, 136.8 (d, $^4J_{C\text{-}F}$ = 3.3 Hz), 136.5, 129.0 (2C), 128.0 (d, 2C, $^3J_{C\text{-}F}$ = 7.7 Hz), 126.4 (2C), 115.1 (d, 2C, $^2J_{C\text{-}F}$ = 21.2 Hz), 20.6. **^{19}F NMR** (376 MHz, $CDCl_3$): δ_F -116.3 (tt, 1F, 1 x ArF, $^3J_{F\text{-}H}$ = 8.7 Hz, $^4J_{F\text{-}H}$ = 5.3 Hz). **^{19}F{^{1}H} NMR** (376 MHz, $CDCl_3$): δ_F -116.3 (s, 1F, 1 x ArF). **m/z** (GCMS EI): 186.1 (M^+). NMR data are consistent with the literature.[4]

4-(difluoromethoxy)-4'-methyl-1,1'-biphenyl

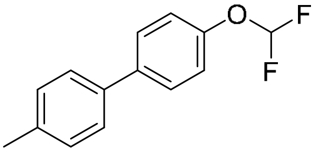

Synthesised according to the General Procedure A using 1-bromo-4-(difluoromethoxy)benzene (137 µL, 223.4 mg, 1 mmol), 4-tolylboronic acid (135.4 mg, 1 mmol), [$PdCl_2$(dppf)] (6.8 mg, 1 mol %) and K_3PO_4 (637.5 mg, 3 mmol) in 2 mL toluene. The desired product was purified *via* flash column chromatography (eluting with hexane) to yield a white solid (182.7 mg, 78 %).

^{1}H NMR (400 MHz, $CDCl_3$): δ_H 7.58 (d, 2H, 2 x ArH, J = 8.8 Hz), 7.48 (d, 2H, 2 x ArH, J = 8.2 Hz), 7.28 (d, 2H, 2 x ArH, J = 8.0 Hz), 7.20 (d, 2H, 2 x ArH, J = 8.5 Hz), 6.56 (t, 1H, CF_2H, J = 74.2 Hz), 2.42 (s, 3H, CH_3). **^{13}C{^{1}H} NMR** (101 MHz, $CDCl_3$): δ_C 149.9, 138.1, 136.8, 136.7, 129.1 (2C), 127.8 (2C), 126.4 (2C), 119.3 (2C), 115.5 (t, $^1J_{C\text{-}F}$ = 259.7 Hz), 20.6. **^{19}F NMR** (376 MHz, $CDCl_3$): δ_F -80.6 (d, 2F, CHF_2, J = 73.8 Hz). **m/z** (GCMS EI): 234.1 (M^+).

4-methyl-4'-(trifluoromethoxy)-1,1'-biphenyl

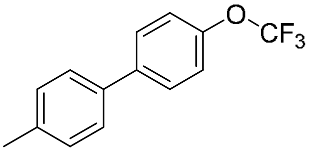

Synthesised according to the General Procedure A using 1-bromo-4-(trifluoromethoxy)benzene (149 µL, 241.7 mg, 1 mmol), 4-tolylboronic acid (136.7 mg, 1 mmol), [$PdCl_2$(dppf)] (7.1 mg, 1 mol %) and K_3PO_4 (641.9 mg, 3 mmol) in 2 mL toluene. The desired product was purified *via* flash column chromatography (eluting with hexane) to yield a white solid (180.1 mg, 71 %).

^{1}H NMR (400 MHz, $CDCl_3$): δ_H 7.60 (d, 2H, 2 x ArH, J = 8.2 Hz), 7.48 (d, 2H, 2 x ArH, J = 8.2 Hz), 2.42 (s, 3H, CH_3). **^{13}C{^{1}H} NMR** (101 MHz, $CDCl_3$): δ_C 148.0, 139.4, 137.0, 136.5, 129.1 (2C), 127.7 (2C), 126.4 (2C), 120.7 (2C), 119.6 (q, $^1J_{C\text{-}F}$ = 256.9 Hz), 20.6. **^{19}F NMR** (376 MHz, $CDCl_3$): δ_F -57.8 (s, 3F, OCF_3). **m/z** (GCMS EI): 252.1 (M^+). NMR data are consistent with the literature.[9]

4'-methyl-4-ispropyl-1,1'-biphenyl

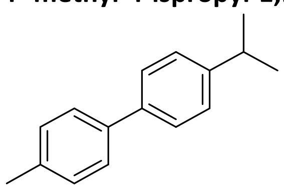

Synthesised according to the General Procedure A using 1-bromo-4-isopropylbenzene (155 µL, 199.3 mg, 1 mmol), 4-tolylboronic acid (135.7 mg, 1 mmol), [$PdCl_2$(dppf)] (7.3 mg, 1 mol %) and K_3PO_4 (633.3 mg, 3 mmol) in 2 mL toluene. The desired product was purified *via* flash column chromatography (eluting with hexane) to yield a white solid (155.0 mg, 74 %).

^{1}H NMR (400 MHz, $CDCl_3$): δ_H 7.55 – 7.50 (m, 4H, 4 x ArH, *J* = 20.4 Hz), 7.31 (d, 2H, 2 x ArH, *J* = 7.9 Hz), 7.26 (d, 2H, 2 x ArH, *J* = 7.9 Hz), 2.97 (h, 1H, C(CH_3)$_2$H, *J* = 7.1 Hz), 2.41 (s, 3H, CH_3), 1.31 (d, 6H, (CH_3)$_2$, *J* = 6.9 Hz). **^{13}C{^{1}H} NMR** (101 MHz, $CDCl_3$): δ_C 147.2, 138.2, 137.8, 136.2, 128.9 (2C), 126.4 (2C), 126.4 (2C), 126.3 (2C), 33.3, 23.5 (2C), 20.6. **m/z** (GCMS EI): 210.1 (M^+). NMR data are consistent with the literature.[10]

N,N-diethyl-4'-methyl-[1,1'-biphenyl]-4-amine

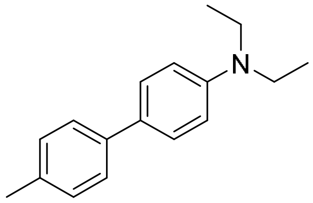

Synthesised according to the General Procedure A using 4-bromo-N,N-diethylaniline (228.2 mg, 1 mmol), 4-tolylboronic acid (135.5 mg, 1 mmol), [$PdCl_2$(dppf)] (7.2 mg, 1 mol %) and K_3PO_4 (635.3 mg, 3 mmol) in 2 mL toluene. The desired product was purified *via* flash column chromatography (eluting with 0 – 5 % EtOAc in hexane) to yield a white solid (214.6 mg, 90 %).

^{1}H NMR (400 MHz, $CDCl_3$): δ_H 7.51 – 7.47 (m, 4H, 4 x ArH, *J* = 14.1 Hz), 7.23 (d, 2H, 2 x ArH, *J* = 8.0 Hz), 6.78 (d, 2H, 2 x ArH, *J* = 8.5 Hz), 3.43 (q, 4H, (C**H**$_2$CH$_3$)$_2$, *J* = 7.1 Hz), 2.41 (s, 3H, CH_3), 1.23 (t, 6H, (CH$_2$C**H**$_3$)$_2$, *J* = 7.1 Hz). **^{13}C{^{1}H} NMR** (101 MHz, $CDCl_3$): δ_C 146.5, 138.0, 134.9, 128.9 (2C), 127.7, 127.3 (2C), 125.6 (2C), 111.5 (2C), 43.9 (2C), 20.6, 12.2 (2C). **m/z** (GCMS EI): 239.4 (M^+).

2,2,2-trifluoro-1-(4'-methyl-[1,1'-biphenyl]-4-yl)ethan-1-one

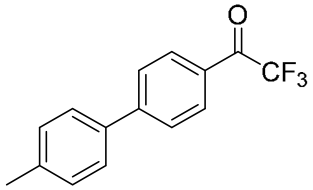

Synthesised according to the General Procedure A using 1-(4-bromophenyl)-2,2,2-trifluoroethan-1-one (253.0 mg, 1 mmol), 4-tolylboronic acid (134.8 mg, 1 mmol), [$PdCl_2$(dppf)] (7.1 mg, 1 mol %) and K_3PO_4 (638.2 mg, 3 mmol) in 2 mL toluene. The desired product was purified *via* flash column chromatography (eluting with 0 – 5 % EtOAc in hexane) to yield a white solid (178.6 mg, 68 %).

^{1}H NMR (400 MHz, $CDCl_3$): δ_H 8.17 (d, 2H, 2 x ArH, *J* = 7.6 Hz), 7.79 (d, 2H, 2 x ArH, *J* = 8.7 Hz), 7.59 (d, 2H, 2 x ArH, *J* = 8.2 Hz), 7.34 (d, 2H, 2 x ArH, *J* = 8.0 Hz), 2.46 (s, 3H, CH_3). **^{13}C{^{1}H} NMR** (101 MHz, $CDCl_3$): δ_C 179.6 (q, $^2J_{C\text{-}F}$ = 34.5 Hz), 147.7, 138.6, 135.7, 130.3 (2C), 129.4 (2C), 127.8 (2C), 126.8, 126.7 (2C), 116.4 (q, $^1J_{C\text{-}F}$ = 291.1 Hz), 20.7. **^{19}F NMR** (376 MHz, $CDCl_3$): δ_F -71.3 (s, 3F, CF_3). **m/z** (GCMS EI): 264.3 (M^+). NMR data are consistent with the literature.[11]

4'-methyl-N-phenyl-[1,1'-biphenyl]-4-amine

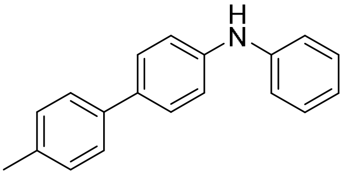

Synthesised according to the General Procedure A using 4-bromo-N-phenylaniline (248.1 mg, 1 mmol), 4-tolylboronic acid (135.5 mg, 1 mmol), [$PdCl_2$(dppf)] (7.4 mg, 1 mol %) and K_3PO_4 (637.4 mg, 3 mmol) in 2 mL toluene. The desired product was purified *via* flash column chromatography (eluting with 0 – 5 % EtOAc in hexane) to yield a white solid (216.0 mg, 83 %).

^{1}H NMR (400 MHz, $CDCl_3$): δ_H 7.52 (t, 5H, 5 x ArH, *J* = 8.4 Hz), 7.32 (t, 3H, 3 x ArH, *J* = 6.9 Hz), 7.26 (d, 3H, 3 x ArH, *J* = 7.7 Hz), 7.17 (bs, 4H, 4 x ArH), 6.99 (bs, 1H, 1 x ArH), 2.42 (s, 3H, CH_3). **$^{13}C\{^1H\}$ NMR** (101 MHz, $CDCl_3$): δ_C 142.5, 141.7, 137.5, 135.8 (2C), 133.4, 129.0 (2C), 128.9 (2C), 127.3 (2C), 125.9 (2C), 120.7 (2C), 117.5 (2C), 20.6 . **m/z** (GCMS EI): 259.4 (M^+).

4-methyl-4'-(methylsulfonyl)-1,1'-biphenyl

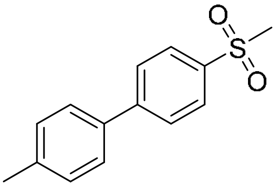

Synthesised according to the General Procedure A using 1-bromo-4-(methylsulfonyl)benzene (235.4 mg, 1 mmol), 4-tolylboronic acid (135.2 mg, 1 mmol), [$PdCl_2$(dppf)] (7.0 mg, 1 mol %) and K_3PO_4 (635.4 mg, 3 mmol) in 2 mL toluene. The desired product was purified *via* flash column chromatography (eluting with 0 – 10 % EtOAc in hexane) to yield a white solid (174.1 mg, 71 %).

^{1}H NMR (400 MHz, $CDCl_3$): δ_H 8.02 (d, 2H, 2 x ArH, *J* = 7.8 Hz), 7.79 (d, 2H, 2 x ArH, *J* = 7.4 Hz), 7.55 (d, 2H, 2 x ArH, *J* = 7.4 Hz), 7.33 (d, 2H, 2 x ArH, *J* = 7.8 Hz), 3.12 (s, SO_2CH_3), 2.46 (s, 3H, CH_3). **$^{13}C\{^1H\}$ NMR** (101 MHz, $CDCl_3$): δ_C 146.2, 138.3 (2C), 135.7, 129.3 (2C), 127.4 (2C), 127.2 (2C), 126.7 (2C), 44.2, 20.7. **m/z** (GCMS EI): 246.3 (M^+). NMR data are consistent with the literature.[12]

3-methoxy-4’-methyl-1,1’-biphenyl

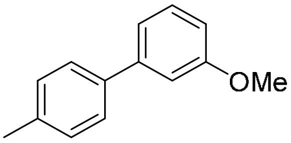

Synthesised according to the General Procedure A using 3-bromoanisole (180 µL, 266 mg, 1.4 mmol), 4-tolylboronic acid (247.0 mg, 1.8 mmol), [$PdCl_2$(dppf)] (41.9 mg, 4.4 mol%), and K_3PO_4 (902.0 mg, 4.2 mmol) in 5 mL toluene. The desired product was purified *via* flash column chromatography (eluting with hexane) to yield a pale oil (120.5 mg, 43%).

^{1}H NMR (400 MHz, $CDCl_3$): δ_H 7.58 (d, 2H, *J* = 8.1 Hz, Ar CH), 7.43 (t, 1H, *J* = 8.1 Hz, Ar CH), 7.33 (d, 2H, *J* = 7.9 Hz, Ar CH), 7.26 (dt, 1H, *J* = 7.9, 1.4 Hz, Ar CH), 7.22 (t, 1H, *J* = 2.2 Hz, Ar CH), 6.97 (ddd, 1H, *J* = 8.3, 2.5, 0.7 Hz, Ar CH), 3.93 (s, 3H, OMe), 2.48 (s, 3H, Me). **$^{13}C\{^1H\}$ NMR** (101 MHz, $CDCl_3$): δ_C 160.1, 142.8, 138.3, 137.3, 129.8, 129.6, 127.1, 119.6, 112.8, 112.5, 55.4, 21.2. **m/z** (GCMS EI): 198.2 (M^+). NMR data are consistent with the literature.[13]

4-methyl-3-(trifluoromethyl)-1,1'-biphenyl

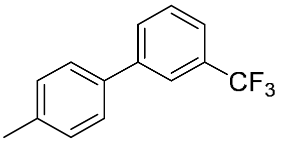

Synthesised according to the General Procedure A using 3-bromobenzotrifluoride (180 µL, 290 mg, 1.3 mmol), 4-tolylboronic acid (206.1 mg, 1.5 mmol), [$PdCl_2$(dppf)] (40.1 mg, 4.2 mol%), and K_3PO_4 (808.9 mg, 3.8 mmol) in 5 mL toluene. The desired product was purified *via* flash column chromatography (eluting with hexane) to yield a colourless oil that solidified upon drying under high vacuum (252.8 mg, 83%).

^{1}H NMR (400 MHz, $CDCl_3$): δ_H 7.90 (s, 1H, Ar CH), 7.81 (d, 1H, *J* = 7.7 Hz, Ar CH), 7.68 – 7.53 (m, 4H, Ar CH), 7.34 (d, 2H, *J* = 8.1 Hz, Ar CH), 2.48 (s, 3H, Me). **^{13}C{^{1}H} NMR** (101 MHz, $CDCl_3$): δ_C 142.1, 138.1, 137.0, 131.3 (q, *J* = 33.0 Hz), 130.8, 130.3, 129.9, 192.3, 127.1, 124.4 (q, *J* = 272.0 Hz), 123.8 (q, *J* = 3.8 Hz), 21.2. **m/z** (GCMS EI): 236.1 (M^+). NMR data are consistent with the literature.[6]

1-(4'-methyl-[1,1'-biphenyl]-3-yl)ethan-1-one

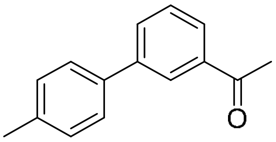

Synthesised according to the General Procedure A using 3'-bromoacetophenone (170 µL, 256 mg, 1.3 mmol), 4-tolylboronic acid (175.7 mg, 1.3 mmol), [$PdCl_2$(dppf)] (42.0 mg, 4.4 mol%), and K_3PO_4 (902.0 mg, 4.2 mmol) in 5 mL toluene. The desired product was purified *via* flash column chromatography (eluting with 0 – 2% Et_2O in hexane) to yield an oil that solidified upon drying under high vacuum (172.4 mg, 64%).

^{1}H NMR (400 MHz, $CDCl_3$): δ_H 8.20 (t, 1H, *J* = 1.7 Hz, Ar CH), 7.94 (dt, 1H, *J* = 7.7, 1.2 Hz, Ar CH), 7.82 – 7.78 (m, 1H, Ar CH), 7.59 – 7.53 (m, 3H, Ar CH), 7.31 (d, 2H, *J* = 8.2 Hz, Ar CH), 2.68 (s, 3H, COMe), 2.44 (s, 3H, Me). **^{13}C{^{1}H} NMR** (101 MHz, $CDCl_3$): δ_C 198.2, 141.7, 137.8, 137.7, 137.4, 131.6, 129.8, 129.1, 127.1, 127.0, 126.8, 26.8, 21.2. **m/z** (GCMS EI): 210.1 (M^+). NMR data are consistent with the literature.[14]

General Procedure B: Synthesis of Chalcone Derivatives

Chalcone. In a round bottom flask equipped with a stirrer bar, aldehyde (1 eq.) and ketone (1 eq.) were dissolved in EtOH (14 mL) or a mixture of EtOH (25 mL) and THF (25 mL). Once dissolved, 10 % w/v solution of NaOH (6 mL) was added. The mixture was stirred for 5-15 minutes at room temperature and the resulting chalcone was filtered and washed with EtOH (3 x 5 mL) to give a white or off-white solid. GC-MS was carried out to check conversion and crude material was carried through to the next step.

Allylic Alcohol. In a round bottom flask equipped with a stirrer bar, chalcone (1 eq.) and $CeCl_3.7H_2O$ (1 eq.) were dissolved in MeOH (10 mL) and THF (50 mL). Once dissolved, the mixture was cooled to 0 ^oC with an ice bath. $NaBH_4$ (1.5 eq.) was slowly added in portions. Once all of the $NaBH_4$ was added, the mixture was warmed to room temperature and stirred for 15 minutes. The reaction was neutralised to pH 7 with 1 M HCl and distilled water (100 mL) was added. The mixture was extracted 3 times with Et_2O (3 x 50 mL). The combined organic phases were dried over $MgSO_4$, which was filtered and the Et_2O removed under vacuum to furnish the allylic alcohol as a white oil. NMR was carried out to check conversion and crude material was carried through to the next step.

Rearrangement to Saturated Chalcone. In a microwave vial equipped with a stirrer bar, allylic alcohol (1 eq.), [IrCl(IPr)(COD)] (0.1 mol%) and KOH (10 mol%) were dissolved (KOH suspended) in THF (2 – 4 mL). The reaction was heated in a Biotage Initiator Microwave Synthesiser at 150 ^oC for 2 – 4 hours. The resulting mixture was filtered through celite and the solvent removed under reduced pressure.^{1}H NMR was carried out to check conversion. The desired compound was either: purified *via* flash column

chromatography, eluting with hexane to yield a white or off-white solid; or recrystallised from hot hexane and filtered to yield a white or off-white solid

1,3-bis(4-chlorophenyl)propan-1-one

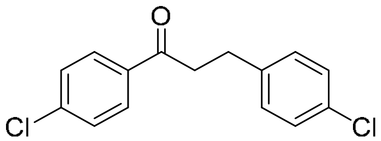

Chalcone. Using 4'-chloroacetophenone (1.1162 g, 1.20 mL, 7.22 mmol) and 4-chlorobenzaldehyde (1.0141 g, 7.22 mmol) to give an off-white solid (1.9724 g, 99 %).
Allylic Alcohol. Using chalcone (1.9724 g, 7.12 mmol), $CeCl_37.H_2O$ (2.6516 g, 7.12 mmol) and $NaBH_4$ (0.40410 g, 10.68 mmol) to yield a white oil (0.5384 g, 27 %).
Saturated Chalcone. Using allylic alcohol (0.5384 g, 1.93 mmol), [IrCl(IPr)(COD)] (0.0014 g, 0.1 mol%) and KOH (0.0081 g, 10 mol%). The product was purified *via* flash column chromatography, eluting with hexane to yield a white solid (286.3 mg, 53 %).
^{1}H NMR (400 MHz, $CDCl_3$): δ_H 7.89 (d, 2H, 2 x ArH, *J* = 8.0 Hz), 7.44 (d, 2H, 2 x ArH, *J* = 8.0 Hz), 7.27 (d, 2H, 2 x ArH, *J* = 8.0 Hz), 7.18 (d, 2H, 2 x ArH, *J* = 8.4 Hz), 3.25 (t, 2H, CH_2, *J* = 7.5 Hz), 3.04 (t, 2H, CH_2, *J* = 7.5 Hz). **^{13}C{^{1}H} NMR** (101 MHz, $CDCl_3$): δ_C 197.1, 139.1, 139.0, 134.6, 131.5, 129.3 (2C), 128.9 (2C), 128.5 (2C), 128.1 (2C), 39.6, 28.8. **m/z** (GCMS EI): 278.0 (M^+). NMR data are consistent with the literature.[15]

1-([1,1'-biphenyl]-4-yl)-3-(4-chlorophenyl)propan-1-one

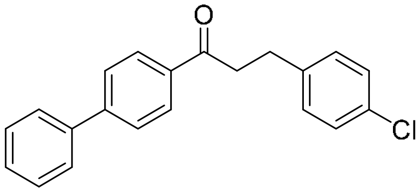

Chalcone. Using 1-([1,1'-biphenyl]-4-yl)ethan-1-one (1.2324 g, 6.27 mmol) and 4-chlorobenzaldehyde (0.8814 g, 6.27 mmol) to give an off-white solid (1.9347 g, 97 %).
Allylic Alcohol. Using chalcone (1.9347 g, 6.07 mmol), $CeCl_37.H_2O$ (2.2805 g, 6.07 mmol) and $NaBH_4$ (0.4731 g, 9.11 mmol) to yield a white oil (0.7032 g, 36 %).
Saturated Chalcone. Using allylic alcohol (0.7032 g, 2.19 mmol), [IrCl(IPr)(COD)] (0.0014 g, 0.1 mol%) and KOH (0.0089 g, 10 mol%). The product was purified *via* recrystallisation from hexane to yield a white solid (469.9 mg, 67 %).
^{1}H NMR (400 MHz, $CDCl_3$): δ_H 8.06 (d, 2H, 2 x ArH, *J* = 8.0 Hz), 7.71 (d, 2H, 2 x ArH, *J* = 8.0 Hz), 7.65 (d, 2H, 2 x ArH, *J* = 7.2 Hz), 7.50 (t, 2H, 2 x ArH, *J* = 8.0 Hz), 7.43 (t, 1H, 1 x ArH, *J* = 8.0 Hz), 7.30 (d, 2H, 2 x ArH, *J* = 8.0 Hz), 7.23 (d, 2H, 2 x ArH, *J* = 8.0 Hz), 3.34 (t, 2H, CH_2, *J* = 7.4 Hz), 3.10 (t, 2H, CH_2, *J* = 7.8 Hz). **^{13}C{^{1}H} NMR** (101 MHz, $CDCl_3$): δ_C 197.9, 145.4, 139.3, 139.3, 135.0, 131.4, 129.3 (2C), 128.5 (2C), 128.1 (4C), 127.8 (2C), 126.8 (3C), 39.7, 29.0. **m/z** (GCMS EI): 320.1 (M^+). NMR data are consistent with the literature.

1,3-di([1,1'-biphenyl]-4-yl)propan-1-one

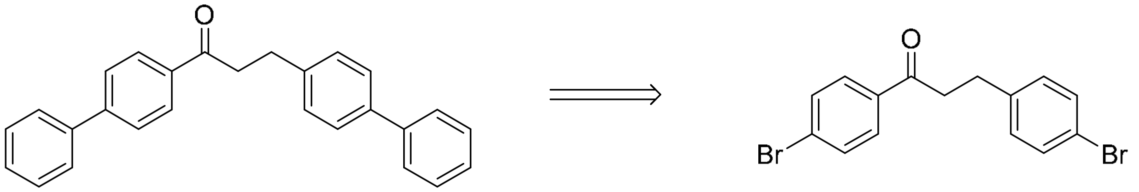

Chalcone (dibromo). Using 4'-bromoacetophenone (1.092 g, 5.46 mmol) and 4-bromobenzaldehyde (1.017 g, 5.46 mmol) to give an off-white solid (1.7970 g, 90 %).
Allylic Alcohol (dibromo). Using chalcone (1.7970 g, 4.88 mmol), $CeCl_37.H_2O$ (1.8192 g, 4.88 mmol) and $NaBH_4$ (0.3773 g, 7.98 mmol) to yield a white oil (1.1037 g, 61 %).

Saturated Chalcone (dibromo). Using allylic alcohol (1.1037 g, 3.00 mmol), [IrCl(IPr)(COD)] (0.0022 g, 0.1 mol%) and KOH (0.0126 g, 10 mol%). The product was purified *via* recrystallisation from hexane to yield a white solid (734.3 mg, 67 %).

Saturated Chalcone (diphenyl). Using dibromo saturated chalcone (734.3 mg, 2.00 mmol), phenyl boronic acid (487.7 mg, 4.00 mmol), [$PdCl_2$(dppf)] (14.9 mg, 1 mol%) and K_3PO_4 (1.3013 g, 6.00 mmol) in 4 mL 4:1 THF:H_2O. The desired product was purified *via* flash column chromatography, eluting with hexane to give a white solid (502.9 mg, 69 %). **^{1}H NMR** (400 MHz, $CDCl_3$): δ_H 8.09 (d, 2H, 2 x ArH, *J* = 8.5 Hz), 7.72 (d, 2H, 2 x ArH, *J* = 8.5 Hz), 7.66 (d, 2H, 2 x ArH, *J* = 7.4 Hz), .61 (d, 2H, 2 x ArH, *J* = 7.4 Hz), 7.58 (d, 2H, 2 x ArH, *J* = 8.1 Hz), 7.52 – 7.43 (m, 5H, 5 x ArH, *J* = 35.9 Hz), 7.39 – 7.37 (m, 3H, 3 x ArH, *J* = 8.1 Hz), 3.41 (t, 2H, CH_2, *J* = 8.0 Hz), 3.18 (t, 2H, CH_2, *J* = 7.6 Hz). **^{13}C{^{1}H} NMR** (101 MHz, $CDCl_3$): δ_C 198.3, 145.3, 140.5, 139.9, 139.4, 138.7, 135.1, 128.5 (2C), 128.4 (2C), 128.3 (2C), 128.2 (2C), 127.7 (2C), 126.8 (4C), 126.6 (2C), 126.5 (2C), 39.9, 29.3. **m/z** (GCMS EI): 362.2 (M^+).

GC-FID CALIBRATION

The GC-FID apparatus was calibrated for each analyte using a series of standards, accurately prepared, containing varying ratios of internal standard and analyte. In each case, a plot of the relative peak areas *versus* the molar ratio gave a straight line, and the slope of this line was used as the response factor.

**Substrate**	**Internal Standard**	**Response Factor**
4-methyl-1,1’-biphenyl	n-dodecane	0.9947
4,4’-dimethyl-1,1’-biphenyl	n-dodecane	0.9516
4-methyl-4’-(trifluoromethyl)-1,1’-biphenyl	n-dodecane	1.0982
methyl 4’-methyl-[1,1’-biphenyl]-4-carboxylate	n-dodecane	0.7552
N,N,4’-trimethyl-[1,1’-biphenyl]-4-amine	n-dodecane	0.2497
4-methoxy-4’-methyl-1,1’-biphenyl	n-dodecane	0.8421
1-(4’-methyl-[1,1’-biphenyl]-4-yl)ethan-1-one	n-dodecane	0.7493
(4’-methyl-[1,1’-biphenyl]-4-yl)(phenyl)methanone	n-dodecane	1.1964
4’-methyl-[1,1’-biphenyl]-4-carbaldehyde	n-dodecane	0.7491
4-fluoro-4'-methyl-1,1'-biphenyl	n-dodecane	0.9598
4-(difluoromethoxy)-4'-methyl-1,1'-biphenyl	n-dodecane	1.1663
4-methyl-4'-(trifluoromethoxy)-1,1'-biphenyl	n-dodecane	1.1355
4’-methyl-4-ispropyl-1,1’-biphenyl	n-dodecane	1.0987
4’-methyl-N-phenyl-[1,1’-biphenyl]-4-amine	n-dodecane	0.7828
N,N-diethyl-4'-methyl-[1,1'-biphenyl]-4-amine	n-dodecane	0.8638
2,2,2-trifluoro-1-(4'-methyl-[1,1'-biphenyl]-4-yl)ethan-1-one	n-dodecane	1.0027
4-methyl-4'-(methylsulfonyl)-1,1'-biphenyl	n-dodecane	0.7081
3-methoxy-4’-methyl-1,1’-biphenyl	n-dodecane	0.9447
4-methyl-3-(trifluoromethyl)-1,1’-biphenyl	n-dodecane	1.0034
1-(4’-methyl-[1,1’-biphenyl]-3-yl)ethan-1-one	n-dodecane	0.9589

DESIGN OF EXPERIMENTS DATA FOR REACTION OPTIMISATION

For each reaction, solid components were loaded into a microwave tube equipped with a stir bar, sealed with a septum-fitted crimp-cap, and evacuated and backfilled with argon or nitrogen several times. The liquid reagents and the reaction solvent were added *via* syringe through the septum. The reactions were then heated, with stirring, for 18 h. After this time, the reaction was cooled to room temperature, and an accurately-known mass of dodecane or tetradecane was added. A sample of the solution was then diluted in chloroform for analysis by GC-FID. The DoE study was initially conducted using the dppe ligand, but comparable results are obtained using dppf under the same conditions. All other work was conducted with dppf as the model nickel(0) complex for kinetic studies and ligand binding studies used a dppf ligand.

Initial Screen

Run	T (°C)	Cat. Loading (mol%)	Boronic Acid equiv.	Base equiv.	Water equiv.	Conversion (%)
1	30	10	1	1	0	0
2	110	1	1	1	0	62
3	110	10	2	1	0	96
4	110	1	2	1	20	0
5	30	1	1	5	0	2
6	110	10	1	5	0	69
7	110	1	2	5	0	80
8	110	10	1	1	20	75
9	30	1	2	5	20	20
10	70	5.5	1.5	3	10	>99
11	30	10	1	5	20	0
12	110	1	1	5	20	78
13	70	5.5	1.5	3	10	>99
14	30	1	1	1	20	5
15	30	10	2	5	0	18
16	30	1	2	1	0	0
17	70	5.5	1.5	3	10	>99
18	30	10	2	1	20	15
19	70	5.5	1.5	3	10	>99
20	110	10	2	5	20	97

Data were analysed using DesignExpert 8. From this, it was deduced that the catalyst loading had a positive effect on conversion, though it was not as significant as the temperature effect. Overall, this initial screen gave positive results, since the centre points appeared to proceed to full conversion. In order to further probe the reaction conditions, and potentially reduce factors such as catalyst loading, the experiment design was augmented to narrow in on optimised conditions.

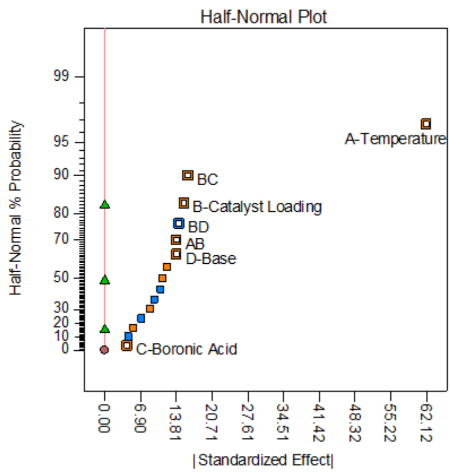

Half-normal plot

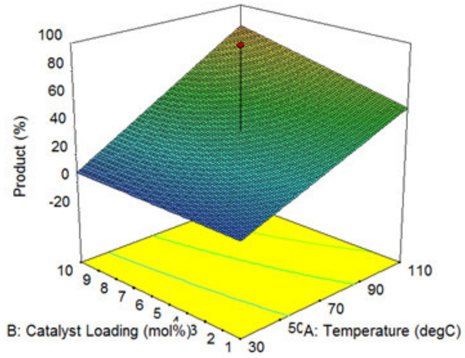

3D response surface

Second Screen

Run	T (°C)	Cat. Loading (mol%)	Boronic Acid eq.	Base eq.	Water eq.	Conversion (%)
21	70	1	1.5	3	10	0
22	70	5.5	1.5	5	10	91
23	70	5.5	1.5	3	0	90
24	70	5.5	1.5	1	10	21
25	110	5.5	1.5	3	10	97
26	70	5.5	1	3	10	81
27	70	5.5	1.5	3	10	98
28	70	5.5	1.5	3	10	97
29	70	5.5	1.5	3	20	58
30	30	5.5	1.5	3	10	0
31	70	5.5	2	3	10	70
32	70	10	1.5	3	10	91

Using these data, the software suggested that the optimum conditions were: 5 mol % catalyst loading; 1.1 equivalents of boronic acid; temperature of 85 °C; 3 equivalents of base; 10 equivalents of water. These conditions were used in triplicate to verify that the reaction was reproducible and tested also on a non-carbonyl substrate.

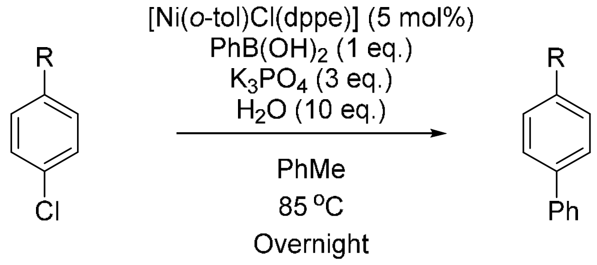

R = COMe, CF_3

Substrate	Conversion (%)
4’chloroacetophenone	92
4’chloroacetophenone	95
4’chloroacetophenone	>99
4-chlorobenzotrifluoride	91

A small time study was conducted, using these optimised conditions, in an effort to reduce the reaction time.

Substrate	Reaction Time (h)	Conversion (%)
4’-chloroacetophenone	2	99*
4’chloroacetophenone	4	99*
4’chloroacetophenone	6	99*
4’chloroacetophenone	8	99*

DATA FROM COMPETITIVE CROSS-COUPLING REACTIONS

Competition Reactions Between Substituted Aryl Bromides and Bromobenzene

Competition reactions carried out between PhBr (1 equiv.) and substrates of the form XC_6H_4Br (1 equiv.). Solid components (catalyst **7**, K_3PO_4, solid substrates) were loaded into a microwave tube equipped with a stir bar, sealed with a septum-fitted crimp-cap, and evacuated and backfilled with Ar or N_2 several times. Liquid reagents (PhBr and the other substrate, if liquid) and the solvent were added *via* syringe through the septum. The reactions were heated and stirred for 2 h, cooled to room temperature, and an accurately-known mass of *n*-dodecane was added. A sample of the solution was diluted in chloroform for analysis by GC-FID.

**Reactions in Toluene Solution**[a]					
**Substrate X =**	**Product from PhBr**	**Product from XC_6H_4Br**	**Total conversion**	**Selectivity**	**σ (X)**
p-NMe_2	59	38	97	-0.23	-0.83
p-NEt_2	60	54	114	-0.05	-0.72
p-NHPh	44	45	99	0.01	-0.56
p-OMe	57	42	99	-0.15	-0.27
p-*i*-Pr	63	33	96	-0.32	-0.15
p-OCF_2H	52	37	89	-0.18	0.18
p-OCF_3	54	40	94	-0.16	0.35
p-CO_2Me	37	76	113	0.35	0.45
p-CF_3	62	48	110	-0.13	0.54
p-SO_2Me	22	78	100	0.56	0.72
p-CHO	8	96	104	0.85	0.42
p-C(O)Ph	11	75	86	0.75	0.43
p-C(O)Me	15	95	110	0.74	0.50
p-$C(O)CF_3$	5	72	77	0.88	0.80
m-CF_3	28	55	83	0.33	0.43
m-OMe	40	31	71	-0.12	0.12
m-Ac	8	78	86	0.81	0.38
**Reactions in THF/Water Solution**[a] [16]					
**Substrate X =**	**Product from PhBr**	**Product from *p*-XC_6H_4Br**	**Total conversion**	**Selectivity**	**σ_p (X)**
NMe_2	37	58	95	0.22	-0.83
NEt_2	43	20	63	-0.37	-0.72
NHPh	42	24	66	-0.28	-0.56
OMe	38	23	61	-0.25	-0.27
i-Pr	38	16	54	-0.40	-0.15
OCF_2H	52	32	84	-0.24	0.18
OCF_3	50	28	78	-0.33	0.35
CO_2Me	32	38	70	0.09	0.45
CF_3	38	35	73	-0.05	0.54
SO_2Me	40	30	70	-0.15	0.72
CHO	0	108	108	1.00	0.42
C(O)Ph	6	58	64	0.83	0.43
C(O)Me	5	95	100	0.91	0.50
$C(O)CF_3$	*N/A – ketone undergoes hydration*				

a – all values quoted as an average of two replicate reactions

Competition Reactions Between Substituted Aryl Bromides and Bromobenzene with Alternative Catalysts

Values quoted in the manuscript are the average of four replicates

Ligand	Product from *p*-$F_3CC_6H_4Br$	Product from *p*-$OHCC_6H_4Br$	Total Conversion
dppe	0	48	48
	0	47	47
	0	55	55
	0	57	57
Xantphos	0	14	14
	0	13	13
	0	8	8
	0	8	8

Competition Reactions with Boronic Acids

Reaction of 1 equiv. *p*-bromotoluene with 1 equiv. of each of two boronic acids. Yields of each product determined by calibrated GC-FID analysis. Results are quoted as the average of two replicates.

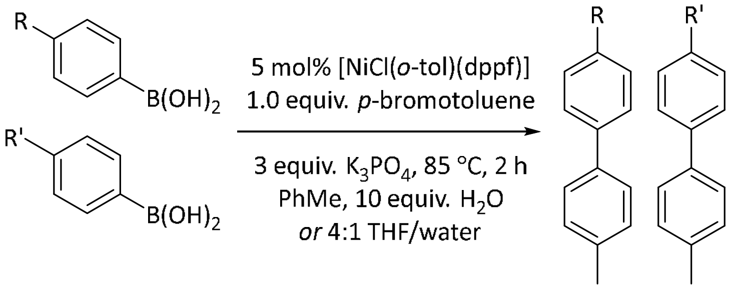

Solvent	R	Yield of Product	R	Yield of Product
THF/water	H	21(1)%	Ac	24(1)%
THF/water	CF_3	16(1)%	Ac	21(1)%
THF/water	H	20(3)%	CF_3	17(2)%
PhMe	H	26(1)%	Ac	7(2)%
PhMe	CF_3	41(3)%	Ac	17(1)%
PhMe	H	33(1)%	CF_3	26(1)%

Competition Reactions Between Different Substituted Aryl Bromides

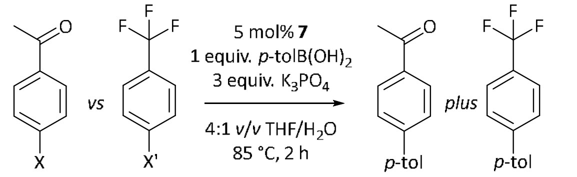

X = Br	X' = Br	91(12)%	:	3%
X = Br	X' = I	71(4)%	:	34(4)%
X = Cl	X' = Cl	104(1)%	:	3(1)%
X = Cl	X' = Br	100(1)%	:	5%
X = Cl	X' = I	62(3)%	:	38(2)%

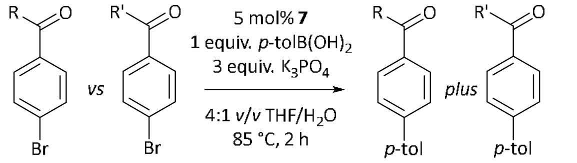

R = Me	R = H	1%	:	80(2)%
R = Me	R = Ph	65(4)%	:	32(1)%
R = H	R = Ph	96(1)%	:	5%
			:	
			:	

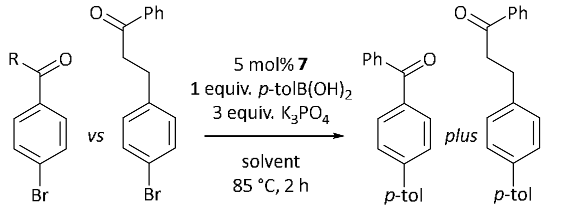

**4-Br** (R = Ph)	**22-Br**	20%	:	<1%
**3-Br** (R = H)	**22-Br**	71(6)%	:	<1%

Intramolecular Competition Reactions

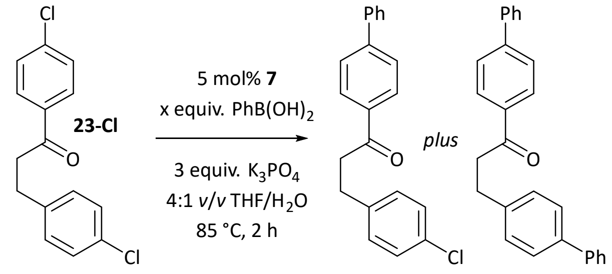

x = 2	20 ± 3%	7 ± 2%
x = 1	14 ± 2%	2 ± 1%
x = 0.5	12 ± 1%	1 ± 1%

DATA FROM ROBUSTNESS SCREENING REACTIONS

A microwave tube equipped with a stir bar was charged with **7** (5 mol%), K_3PO_4 (3 equiv.), *p*-tolB(OH)$_2$ (1.1 equiv.) and the additive (if solid). The tube was sealed with a crimp cap and evacuated and backfilled with nitrogen or argon. 4-(Trifluoromethyl)bromobenzene was added *via* syringe (0.25 mmol, 1 equiv.), followed by the additive (if liquid), anhydrous toluene (1 mL), and degassed distilled water (10 equiv.). The reaction was heated to 85 °C with stirring for 2 h. Upon cooling, the tube was opened, a known mass of tetradecane or dodecane was added, and the mixture was stirred briefly. A sample was withdrawn, diluted with chloroform, and analysed by GC-FID.

Additive	Conversion (%)			Additive Remaining (%)	
	Replicate 1	Replicate 2	Average	Replicate 1	Replicate 2
None	88	93	88	N/A	N/A
Phenyl acetate	75	81	78	60	82
Methyl benzoate	87	89	88	91	98
Benzamide	86	87	86	0 [a]	0 [a]
Benzophenone	33	43	38	100	100
Benzaldehyde	11	13	12	98	100
2,2,2-Trifluoroacetophenone	1	1	1	85 [b]	88 [b]
Acetophenone	73	83	78	92	95
Diphenylamine	72	81	77	100	100
Anisole	82	84	83	100	100
N,*N*-Dimethylaniline	81	90	86	97	100
Methyl phenyl sulfone	85	88	87	7	8

a) Analysis of a control reaction without a nickel catalyst also shows no recovery of the additive.
b) This additive undergoes hydration in the presence of water.

Experiments with Different Catalysts

Ligand	Conversion (%)			
	No additive		1 equiv. PhCHO	
dppe	53	62	10	0
Xantphos	66	79	16	27

Experiment with the Cross-Coupling of 4-Cl

Reaction conducted between **4-Cl** (1 equiv.) and *p*-tolylboronic acid (1.1 equiv.) in the presence of benzaldehyde (1 equiv.) using 5 mol% **7**, 3 equiv. K_3PO_4, and 10 equiv. water in toluene.

Conversion (two replicates): 100% 100%

EQUILIBRIUM CONSTANTS FOR THE BINDING OF ALDEHYDES AND KETONES TO NICKEL(0)

Equilibrium constant determination

$$K_{eq} = \frac{[Ni-L][COD]}{[L][Ni-COD]} = \frac{[Ni-L]([Ni-COD]_0 - [Ni-COD])}{([L]_0 - [Ni-L])[Ni-COD]}$$

L is the aldehyde or ketone, Ni-COD = [Ni(COD)(dppf)], and Ni-L = [Ni(L)(dppf)], with concentrations determined from ^{31}P NMR analyses. A plot of [Ni-L]·([Ni-COD]$_0$ – [Ni-COD]) *versus* ([L]$_0$ – [Ni-L])·[Ni-COD] should yield a straight line of gradient K_{eq}.

Acetophenone

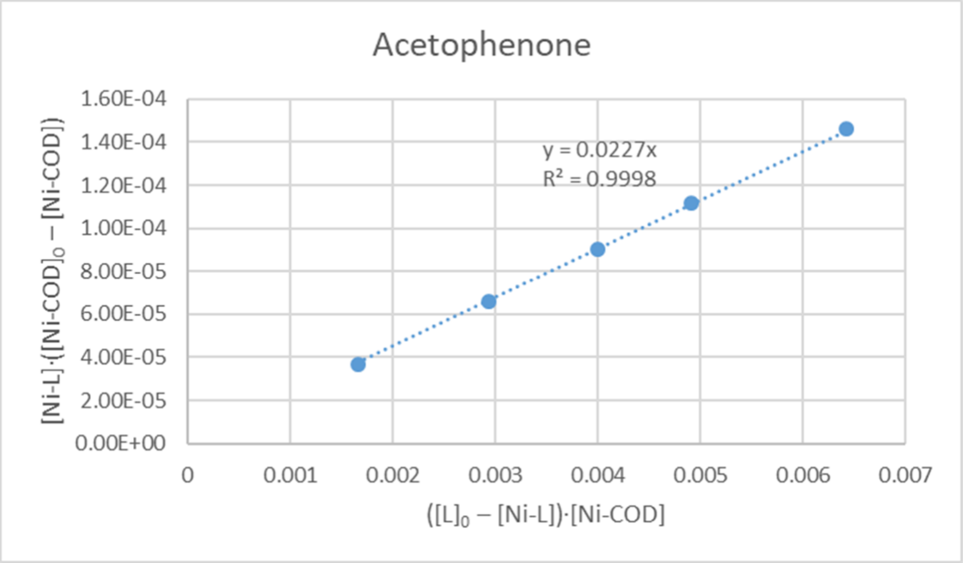

Benzaldehyde

The addition of 1 equiv. benzaldehyde to [Ni(COD)(dppf)] led to complete formation of [Ni(η^2-OHCPh)(dppf)] and so K_{eq} is estimated at > 20.

Benzophenone

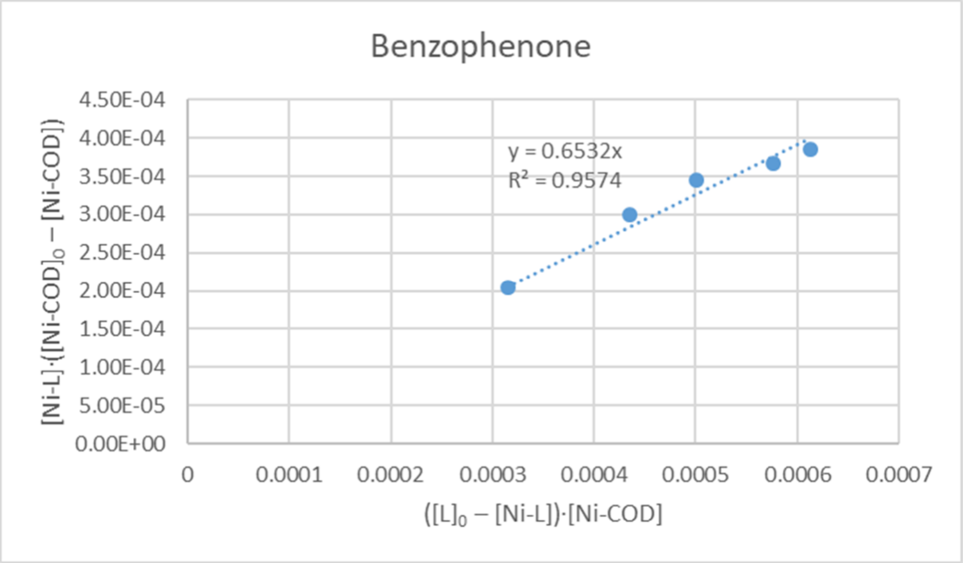

KINETIC DATA FOR OXIDATIVE ADDITION TO NICKEL(0)

Kinetic data were obtained in the same manner as that used for our previous paper.[17] Liquid substrates were added neat to a septum-fitted NMR tube containing a solution of [Ni(COD)(dppf)] (**1**) in benzene-*d*$_6$ that had been equilibrated at 20 °C. For solid substrates, a solution of [Ni(COD)(dppf)] in benzene-*d*$_6$, equilibrated at 20 °C, was added to the solid substrate. ^{31}P NMR spectra were acquired at intervals, with a long D1 (25 seconds) and without ^{1}H decoupling. All kinetic data showed pseudo-first order behaviour in [Ni(COD)(dppf)] and so plots of ln(**1**) *versus* time yielded k_{obs}. Each experiment was performed in duplicate and so rate constants are quoted as the average of these two replicates. Data for **5-Cl**, **5-Br**, **5-I**, and **6-Cl** can be found in our previous manuscript.

Substrate	k_{obs} (rep. 1)	k_{obs} (rep. 2)	k_{obs} (mean)
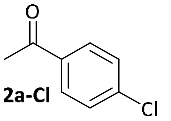 **2a-Cl**	2.20 x 10^{-4} s^{-1}	2.87 x 10^{-4} s^{-1}	2.5(3) x 10^{-4} s^{-1}
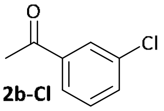 **2b-Cl**	3.89 x 10^{-4} s^{-1}	4.50 x 10^{-4} s^{-1}	4.2(3) x 10^{-4} s^{-1}
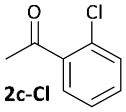 **2c-Cl**	3.25 x 10^{-4} s^{-1}	2.79 x 10^{-4} s^{-1}	3.0(2) x 10^{-4} s^{-1}
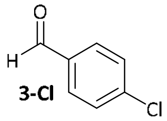 **3-Cl**	1.40 x 10^{-4} s^{-1}	1.42 x 10^{-4} s^{-1}	1.41(1) x 10^{-4} s^{-1}
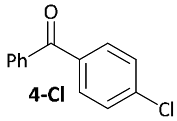 **4-Cl**	9.41 x 10^{-5} s^{-1}	9.06 x 10^{-5} s^{-1}	9.2(2) x 10^{-5} s^{-1}

Kinetic data (integral *versus* time and ln(integral) *versus* time) follow on the subsequent pages. A stack plot is provided for the reaction of **3-Cl** in which signals consistent with an η^2(CO) are observed to appear.

2a-Cl

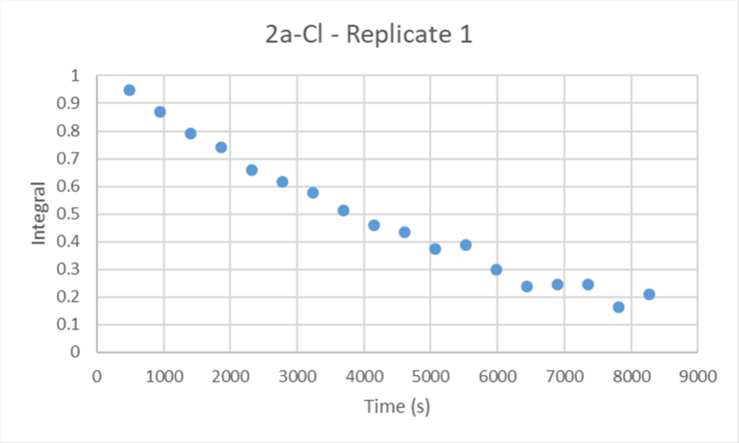
2a-Cl - Replicate 1
Integral
1
0.9
0.8
0.7
0.6
0.5
0.4
0.3
0.2
0.1
0
0
1000
2000
3000
4000
5000
6000
7000
8000
9000
Time (s)

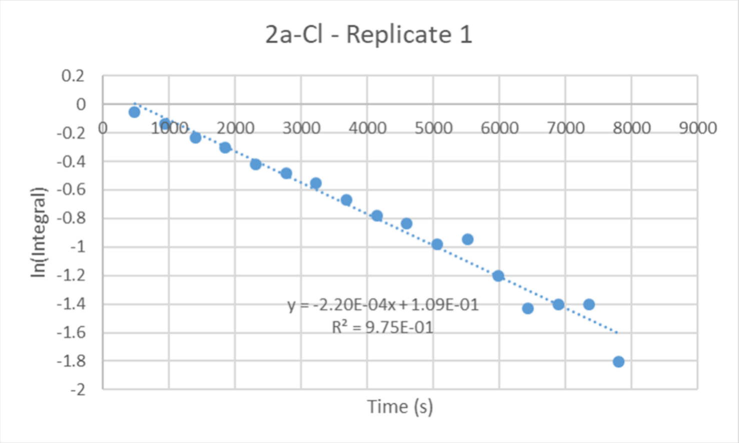
2a-Cl - Replicate 1
ln(Integral)
0.2
0
-0.2
-0.4
-0.6
-0.8
-1
-1.2
-1.4
-1.6
-1.8
-2
0
1000
2000
3000
4000
5000
6000
7000
8000
9000
y = -2.20E-04x + 1.09E-01
R² = 9.75E-01
Time (s)

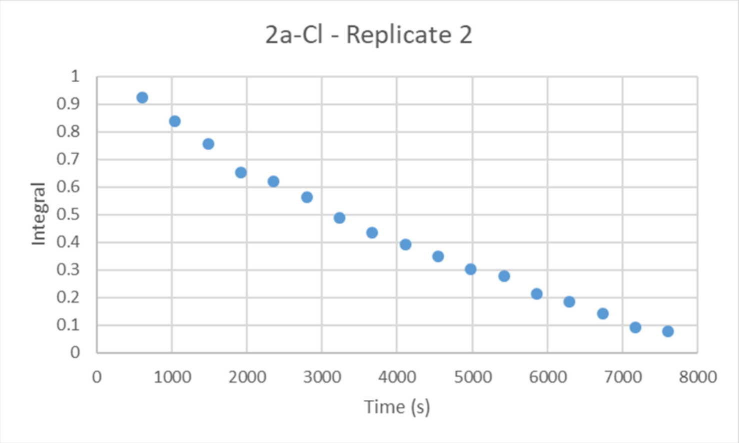
2a-Cl - Replicate 2
Integral
1
0.9
0.8
0.7
0.6
0.5
0.4
0.3
0.2
0.1
0
0
1000
2000
3000
4000
5000
6000
7000
8000
Time (s)

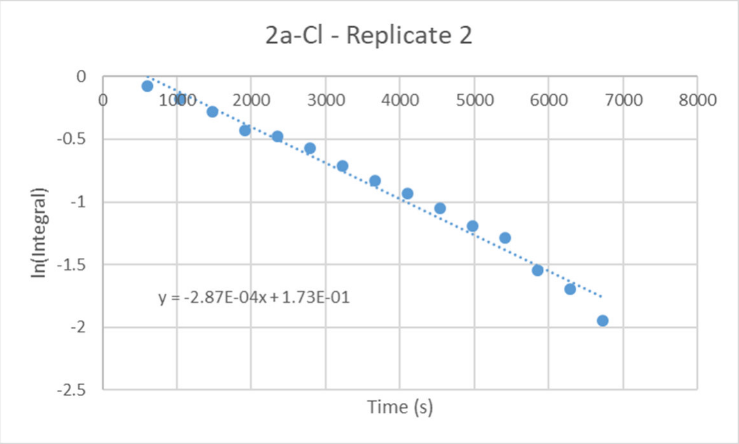
2a-Cl - Replicate 2
ln(Integral)
0
-0.5
-1
-1.5
-2
-2.5
0
1000
2000
3000
4000
5000
6000
7000
8000
y = -2.87E-04x + 1.73E-01
Time (s)

2b-Cl

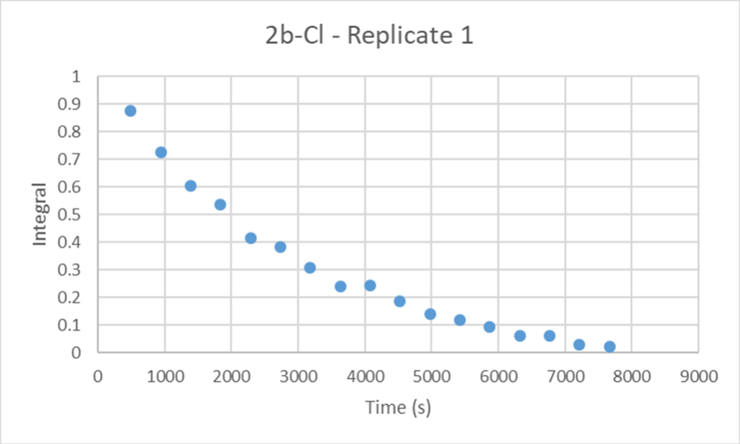
2b-Cl - Replicate 1
Integral
Time (s)

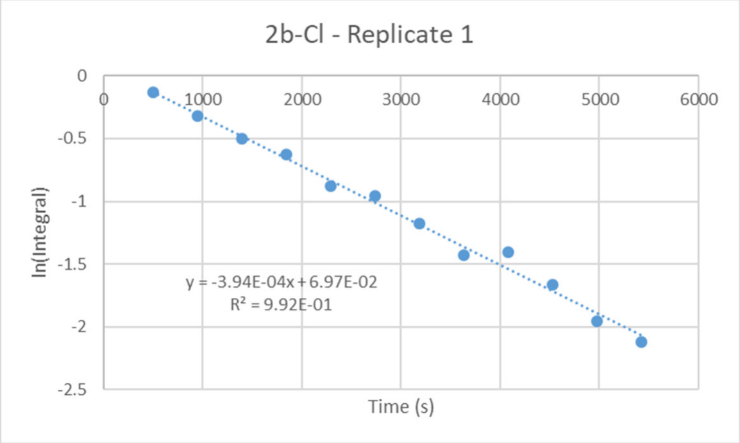
2b-Cl - Replicate 1
ln(Integral)
Time (s)
y = -3.94E-04x + 6.97E-02
R² = 9.92E-01

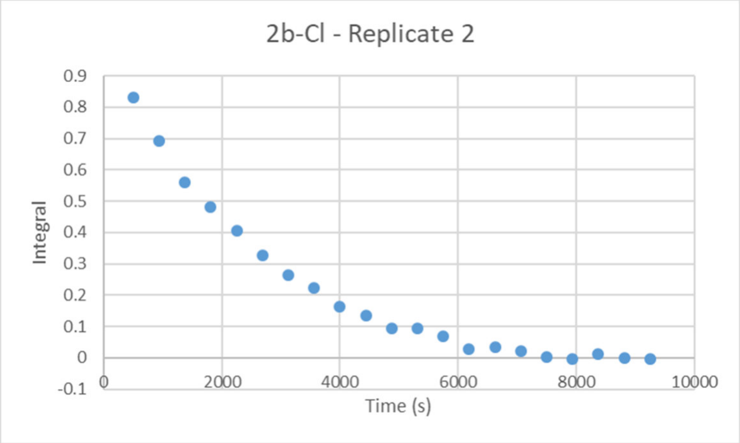
2b-Cl - Replicate 2
Integral
Time (s)

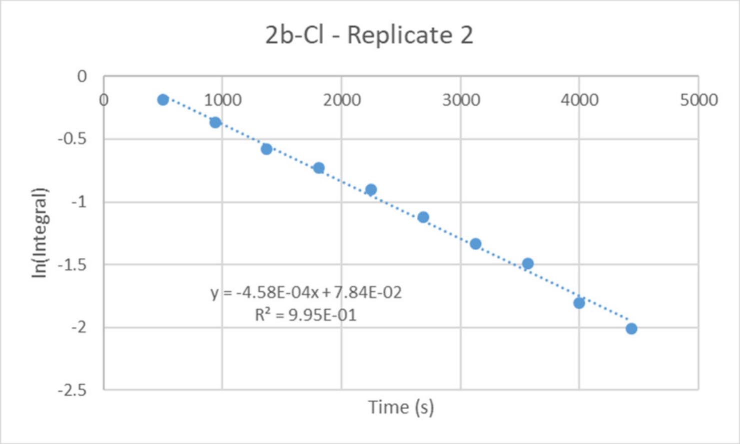
2b-Cl - Replicate 2
ln(Integral)
Time (s)
y = -4.58E-04x + 7.84E-02
R² = 9.95E-01

2c-Cl

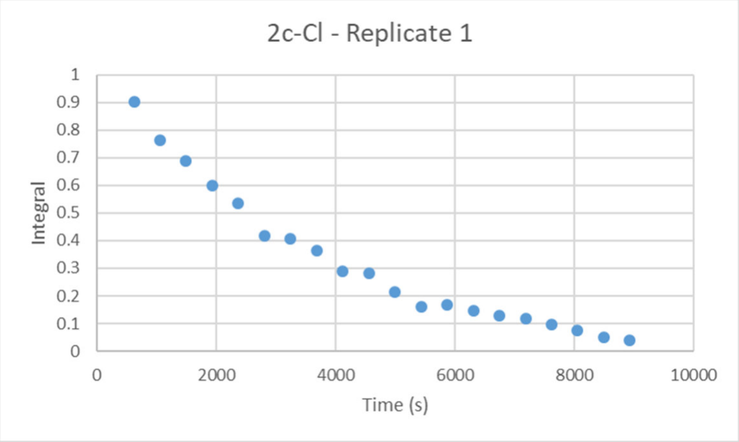
2c-Cl - Replicate 1
Integral
Time (s)
0
0.1
0.2
0.3
0.4
0.5
0.6
0.7
0.8
0.9
1
2000
4000
6000
8000
10000

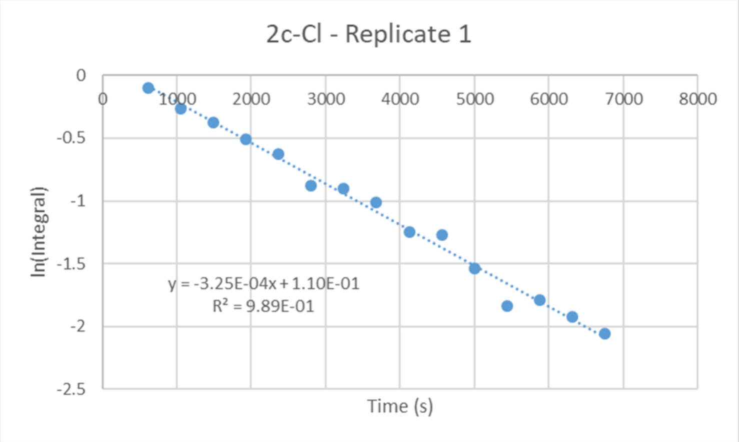
2c-Cl - Replicate 1
ln(Integral)
Time (s)
y = -3.25E-04x + 1.10E-01
R² = 9.89E-01
0
1000
2000
3000
4000
5000
6000
7000
8000
-0.5
-1
-1.5
-2
-2.5

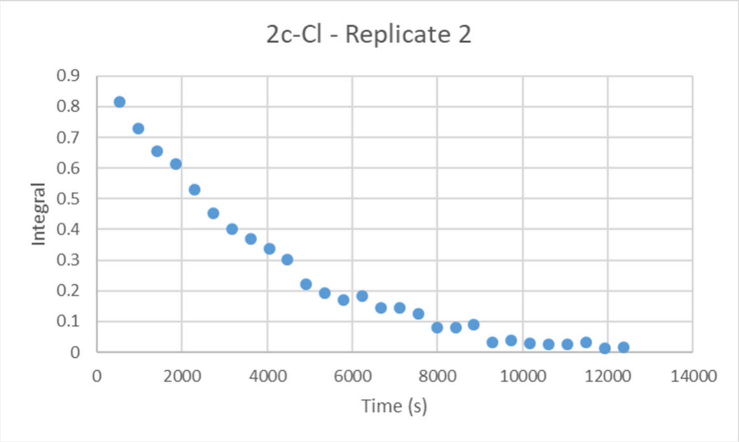
2c-Cl - Replicate 2
Integral
Time (s)
0
0.1
0.2
0.3
0.4
0.5
0.6
0.7
0.8
0.9
2000
4000
6000
8000
10000
12000
14000

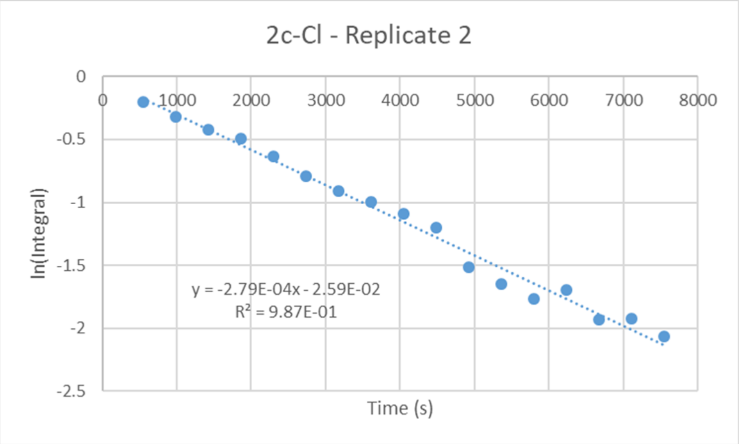
2c-Cl - Replicate 2
ln(Integral)
Time (s)
y = -2.79E-04x - 2.59E-02
R² = 9.87E-01
0
1000
2000
3000
4000
5000
6000
7000
8000
-0.5
-1
-1.5
-2
-2.5

3-Cl

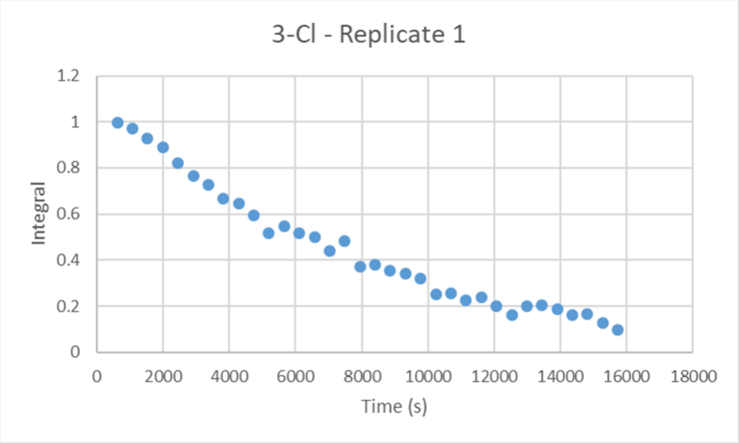

3-Cl - Replicate 1
Integral
Time (s)

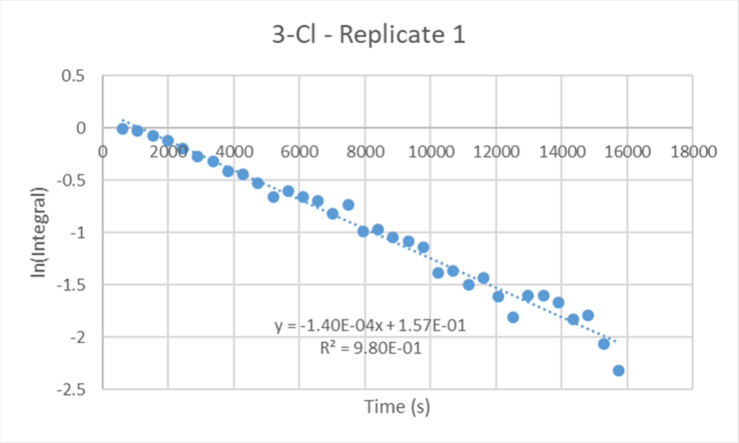

3-Cl - Replicate 1
ln(Integral)
Time (s)
y = -1.40E-04x + 1.57E-01
R² = 9.80E-01

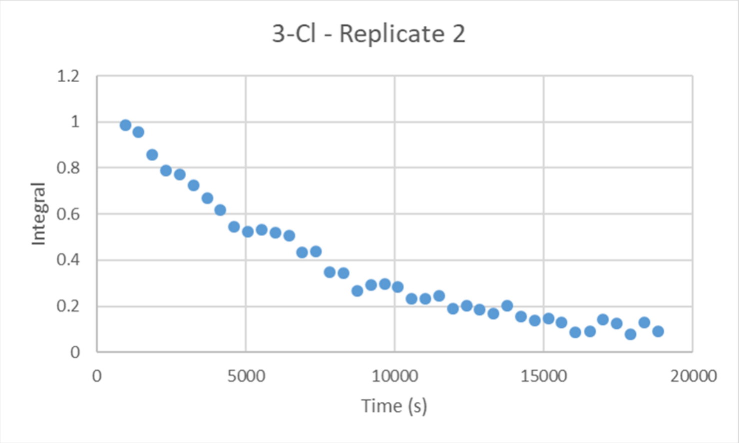

3-Cl - Replicate 2
Integral
Time (s)

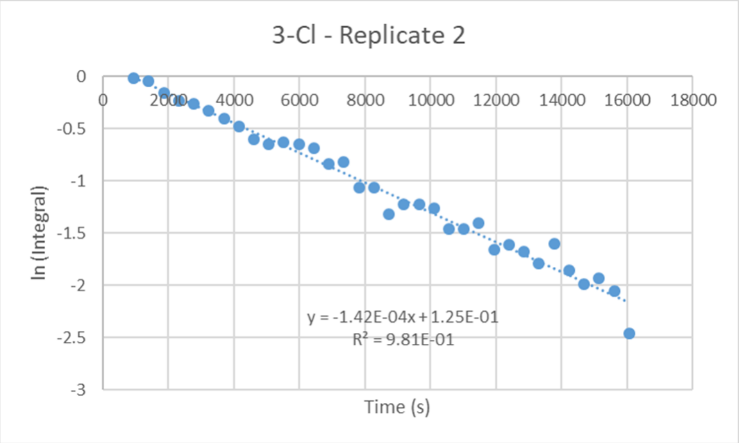

3-Cl - Replicate 2
ln (Integral)
Time (s)
y = -1.42E-04x + 1.25E-01
R² = 9.81E-01

Stack plot for reaction of 1 with 3-Cl (20 equiv.) followed by ^{31}P NMR Spectroscopy (no decoupling) (243 MHz, C_6D_6) at 20 °C.

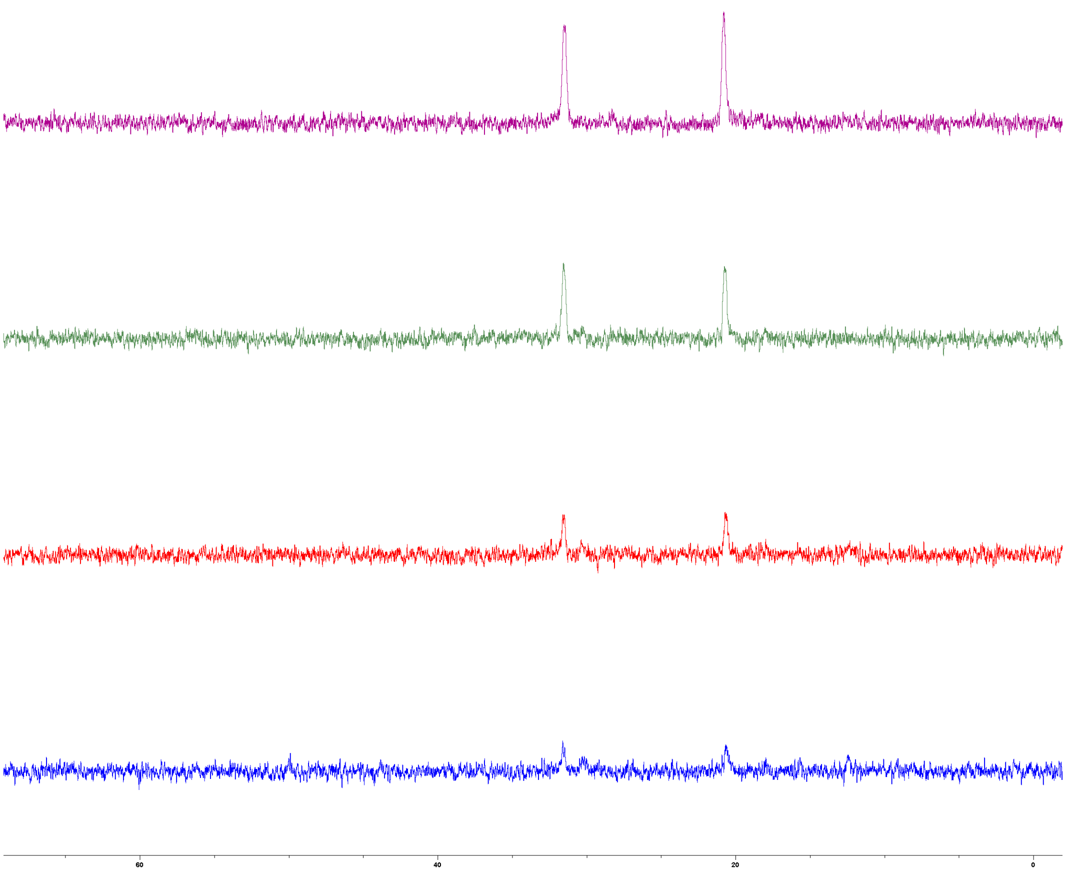

Sample of 1 plus 3-Cl analysed by $^{31}P\{^{1}H\}$ NMR Spectroscopy (162 MHz, C_6D_6) after 5 min.

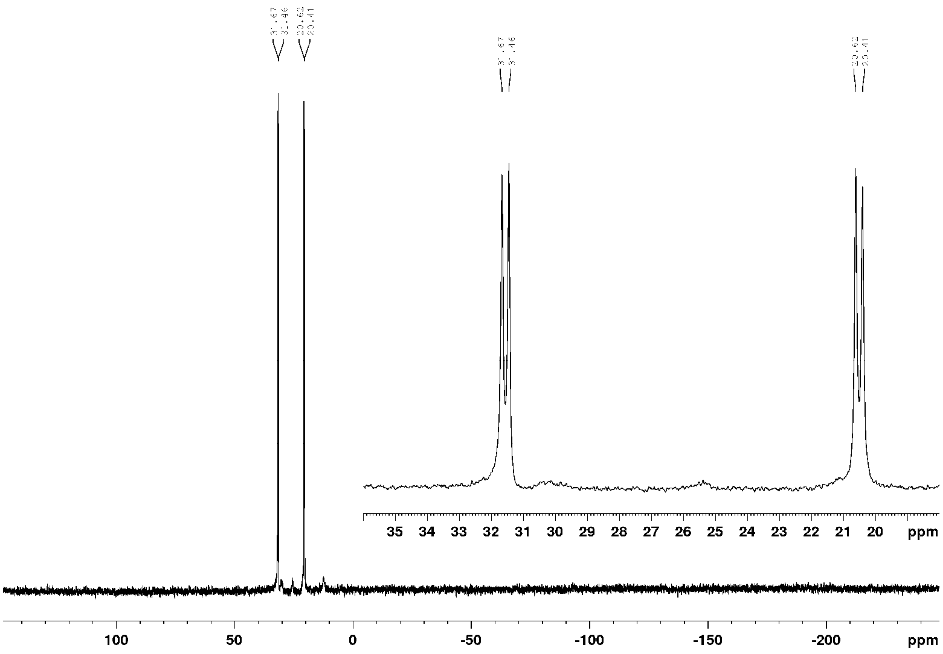

4-Cl

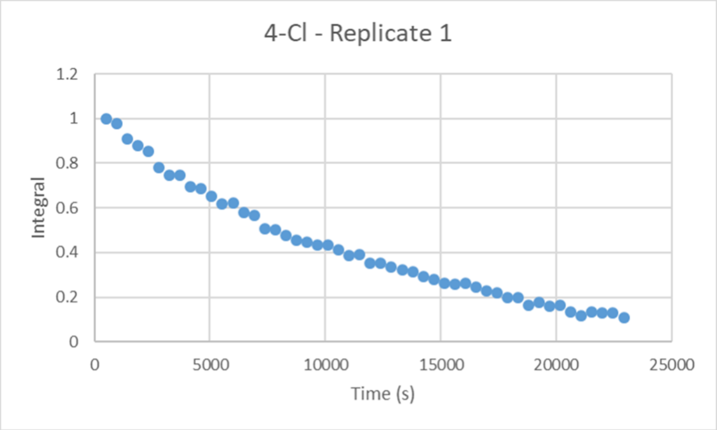
4-Cl - Replicate 1
Integral
Time (s)

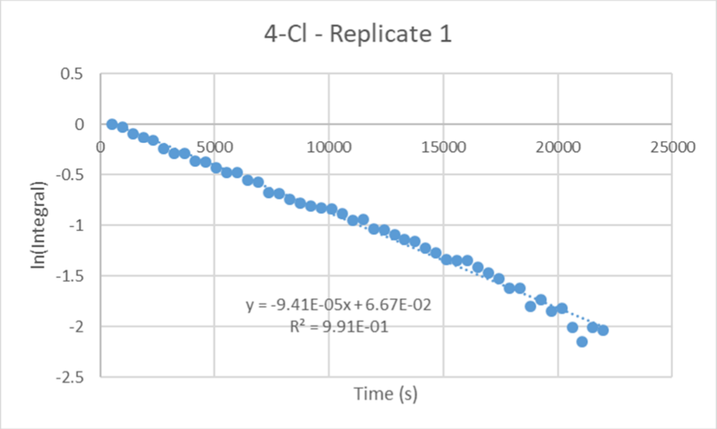
4-Cl - Replicate 1
ln(Integral)
y = -9.41E-05x + 6.67E-02
R² = 9.91E-01
Time (s)

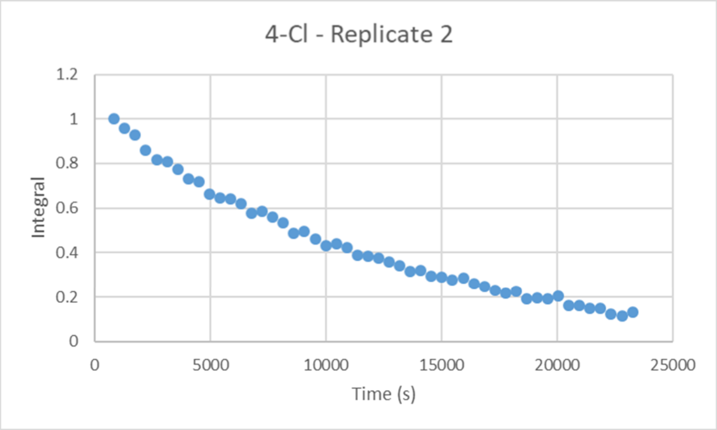
4-Cl - Replicate 2
Integral
Time (s)

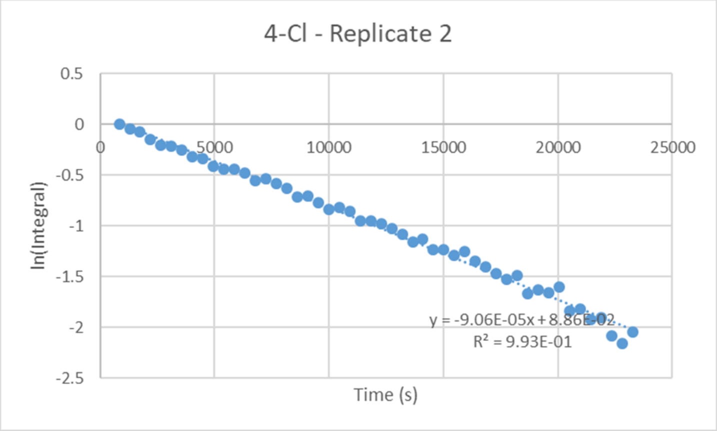
4-Cl - Replicate 2
ln(Integral)
y = -9.06E-05x + 8.86E-02
R² = 9.93E-01
Time (s)

ENERGIES AND COORDINATES FROM DFT CALCULATIONS

Calculations were carried out using Gaussian09 Rev. D01 using desktop machines and the ARCHIE-WeSt High-Performance Computer. The B3LYP functional was used with Grimme's D3 corrections to account for dispersive interactions.[18-20] Solvation (toluene) was treated using the SMD implicit solvation model.[21] The LANL2TZ(f) basis set was used for Ni and Fe, LANL2DZ(d,p) was used for Br, and 6-31G(d) was used for all other atoms for optimisation.[22-25] Geometry optimisation was carried out in solvent, without symmetry constraints. The nature of each stationary point was verified using frequency calculations. Energies were then refined using single point energy calculations with 6-311+G(d,p) for all atoms except Ni, Fe, and Br, which were treated as noted above. Coordinates can be found in a separate Supporting Information file specifically for this content. The energies of intermediates A, B, and C for a series of substrates are tabulated on this page. Energies are tabulated on the following pages in Hartrees. Electronic energies (E), and corrections to enthalpy (Hcorr) and free energy (Gcorr) are reported here with the smaller basis set (6-31G(d) on H/C/N/O/F/P), along with electronic energies using the larger 6-311+G(d,p) basis set on these atoms (denoted E').

***p*-XC_6H_4Br**	**σ_p**	**η^2-complex (A)**	**OA TS (B)**	**[Ni(Ar)Br(dppf)] (C)**	**$\Delta G^{\ddagger\ a}$**
X = SO_2Me	0.72	-8.0	1.1	-25.3	9.1
X = CF_3	0.54	-6.4	1.9	-24.3	8.3
X = Ac	0.50	-6.9	2.2	-23.4	9.1
X = CO_2Me	0.45	-6.0	2.1	-22.6	8.1
X = CHO	0.42	-8.5	0.8	-24.5	9.4
X = OCF_3	0.35	-6.0	3.2	-23.9	9.3
X = H	0	-2.6	4.7	-21.8	7.3
X = OMe	-0.27	-0.5	5.8	-18.6	6.2
X = NHPh	-0.56	-2.9	4.6	-18.8	7.4
X = NMe_2	-0.83	-1.1	6.3	-17.2	7.4

a) Defined as the difference in energy between A and C.

				[Benzene]	SO_2Me	CF_3	COMe	CO_2Me	CHO
			σ_p		0.72	0.54	0.5	0.45	0.42
Arene	Small Basis	Hartrees	E	-232.256720814	-832.727169108	-581.869595304	-397.486124379	-472.717048769	-358.159249082
			H_{corr}	0.106172000	0.140283000	0.105610000	0.138462000	0.145097000	0.108648000
			G_{corr}	0.073387000	0.090432000	0.058212000	0.092200000	0.096084000	0.066092000
			H	-232.150548814	-832.586886108	-581.763985304	-397.347662379	-472.571951769	-358.050601082
			G	-232.183333814	-832.636737108	-581.811383304	-397.393924379	-472.620964769	-358.093157082
		kcal	H	-145676.668775084	-522456.158960929	-365062.412410257	-249339.422614576	-296543.376881719	-224680.144350350
			G	-145697.241673190	-522487.440935717	-365092.155104305	-249368.452457862	-296574.133003568	-224706.848643525
	Large Basis	Hartrees	E	-232.324323739	-832.880895448	-582.049284916	-397.597073193	-472.852496660	-358.259540840
			H	-232.218151739	-832.740612448	-581.943674916	-397.458611193	-472.707399660	-358.150892840
			G	-232.250936739	-832.790463448	-581.991072916	-397.504873193	-472.756412660	-358.193448840
		kcal	H	-145719.090250992	-522552.623695682	-365175.169344166	-249409.044046490	-296628.371716554	-224743.078378659
			G	-145739.663149097	-522583.905670471	-365204.912038215	-249438.073889776	-296659.127838404	-224769.782671834
η^2-(*ipso*/*ortho*)-complex	Small Basis	Hartrees	E	-2520.387200460	-3120.880169300	-2870.018811360	-2685.634914720	-2760.865643300	-2646.308945950
			H_{corr}	0.660970000	0.696137000	0.661200000	0.694285000	0.701034000	0.664514000
			G_{corr}	0.549576000	0.570303000	0.538513000	0.571746000	0.576422000	0.545317000
			H	-2519.726230460	-3120.184032300	-2869.357611360	-2684.940629720	-2760.164609300	-2645.644431950
			G	-2519.837624460	-3120.309866300	-2869.480298360	-2685.063168720	-2760.289221300	-2645.763628950
		kcal	H	-1581152.081499960	-1957945.040891770	-1800549.085422410	-1684825.682276830	-1732029.442135260	-1660166.945883970
			G	-1581221.982290	-1958024.002919	-1800626.072677	-1684902.576660	-1732107.637346	-1660241.743131
	Large Basis	Hartrees	E	-2520.85945707	-3121.43661902	-2870.60010695	-2686.14888294	-2761.40336165	-2646.81309153
			H	-2520.19848707	-3120.74048202	-2869.93890695	-2685.45459794	-2760.70232765	-2646.14857753
			G	-2520.30988107	-3120.86631602	-2870.06159395	-2685.57713694	-2760.82693965	-2646.26777453
		kcal	H	-1581448.42699689	-1958294.21836288	-1800913.85391233	-1685148.20220421	-1732366.86549423	-1660483.30201170
			G	-1581518.32778724	-1958373.18039003	-1800990.84116717	-1685225.09658765	-1732445.06070480	-1660558.09925847
			G_{rel}	**0.0**	**-10.6**	**-7.3**	**-8.4**	**-7.3**	**-9.7**

				OCF$_3$	H	OMe	NHPh	NMe$_2$
			σ_p	0.35	0	-0.27	-0.56	-0.83
Arene	Small Basis	Hartrees	E	-657.087443715	-244.832936134	-359.358545579	-531.249941422	-378.808962646
			H_{corr}	0.110681000	0.097386000	0.132758000	0.201136000	0.175255000
			G_{corr}	0.060866000	0.059788000	0.089051000	0.148319000	0.127395000
			H	-656.976762715	-244.735550134	-359.225787579	-531.048805422	-378.633707646
			G	-657.026577715	-244.773148134	-359.269494579	-531.101622422	-378.681567646
		kcal	H	-412259.142801512	-153573.876333687	-225417.585010934	-333238.156558688	-237596.238723611
			G	-412290.402185960	-153597.469434890	-225445.011567514	-333271.299726576	-237626.271327037
	Large Basis	Hartrees	E	-657.288836630	-244.897180158	-359.458066135	-531.393902653	-378.911583045
			H	-657.178155630	-244.799794158	-359.325308135	-531.192766653	-378.736328045
			G	-657.227970630	-244.837392158	-359.369015135	-531.245583653	-378.784188045
		kcal	H	-412385.518763671	-153614.190067395	-225480.035102682	-333328.493595029	-237660.633996209
			G	-412416.778148119	-153637.783168598	-225507.461659262	-333361.636762917	-237690.666599635
η^2-(*ipso*/*ortho*)-complex	Small Basis	Hartrees	E	-2945.231076650	-2532.971304160	-2647.493273320	-2819.393139210	-2666.948327140
			H_{corr}	0.666165000	0.652631000	0.687881000	0.756907000	0.730644000
			G_{corr}	0.539214000	0.537172000	0.566085000	0.627999000	0.606832000
			H	-2944.564911650	-2532.318673160	-2646.805392320	-2818.636232210	-2666.217683140
			G	-2944.691862650	-2532.434132160	-2646.927188320	-2818.765140210	-2666.341495140
		kcal	H	-1847742.378868350	-1589053.958595010	-1660895.459515090	-1768720.939471440	-1673076.855916680
			G	-1847822.041824	-1589126.410211	-1660971.887659	-1768801.830463	-1673154.549120
	Large Basis	Hartrees	E	-2945.83656905	-2533.43881692	-2647.99621938	-2819.93951176	-2667.453336
			H	-2945.17040405	-2532.78618592	-2647.30833838	-2819.18260476	-2666.722692
			G	-2945.29735505	-2532.90164492	-2647.43013438	-2819.31151276	-2666.846504
		kcal	H	-1848122.33108578	-1589347.32728113	-1661211.06293265	-1769063.79342290	-1673393.753893
			G	-1848201.99404102	-1589419.77889748	-1661287.49107654	-1769144.68441417	-1673471.447096
			G_{rel}	**-6.6**	**-3.3**	**-1.4**	**-4.4**	**-2.1**

				SO_2Me	*CF_3*	*COMe*	*CO_2Me*	*CHO*
			σ_p	0.72	0.54	0.5	0.45	0.42
Oxidative Addition TS	Small Basis	Hartrees	**E**	-3120.86698347	-2870.00637787	-2685.61999239	-2760.85059089	-2646.29413396
			**H_{corr}**	0.69555000	0.66044400	0.69299800	0.69957800	0.66315900
			**G_{corr}**	0.57085800	0.53651200	0.56987800	0.57343600	0.54291600
			**H**	-3120.17143347	-2869.34593387	-2684.92699439	-2760.15101289	-2645.63097496
			**G**	-3120.29612547	-2869.46986587	-2685.05011439	-2760.27715489	-2645.75121796
		kcal	**H**	-1957937.13500659	-1800541.75768680	-1684817.12597807	-1732020.91025917	-1660158.50149526
			**G**	-1958015.38041792	-1800619.52619093	-1684894.38494451	-1732100.06555924	-1660233.95511694
	Large Basis	Hartrees	**E**	-3121.42235647	-2870.58673864	-2686.13383947	-2761.38886448	-2646.79753807
			**H**	-3120.72680647	-2869.92629464	-2685.44084147	-2760.68928648	-2646.13437907
			**G**	-3120.85149847	-2870.05022664	-2685.56396147	-2760.81542848	-2646.25462207
		kcal	**H**	-1958285.63682569	-1800905.93956832	-1685139.56988896	-1732358.68203650	-1660474.39234353
			**G**	-1958363.88223702	-1800983.70807245	-1685216.82885540	-1732437.83733657	-1660549.84596521
			**G_{rel}**	**-1.3**	**-0.1**	**-0.1**	**0.0**	**-1.4**
η2 to TS	Small Basis	kcal	**$\Delta G^{\ddagger}$**	**8.6**	**6.5**	**8.2**	**7.6**	**7.8**
	Large Basis		**$\Delta G^{\ddagger}$**	**9.3**	**7.1**	**8.3**	**7.2**	**8.3**

				OCF_3	*H*	*OMe*	*NHPh*	*NMe_2*
			σ_p	0.35	0	-0.27	-0.56	-0.83
Oxidative Addition TS	Small Basis	Hartrees	E	-2945.22124005	-2532.96360392	-2647.48692276	-2819.38002048	-2666.938074
			H_{corr}	0.66530400	0.65171500	0.68709800	0.75558300	0.729564
			G_{corr}	0.53758300	0.53523500	0.56419900	0.62558500	0.604492
			H	-2944.55593605	-2532.31188892	-2646.79982476	-2818.62443748	-2666.208510
			G	-2944.68365705	-2532.42836892	-2646.92272376	-2818.75443548	-2666.333582
		kcal	H	-1847736.74659431	-1589049.70142014	-1660891.96581844	-1768713.53816662	-1673071.099879
			G	-1847816.89273184	-1589122.79372367	-1660969.08610528	-1768795.11314322	-1673149.583744
	Large Basis	Hartrees	E	-2945.82509057	-2533.42949762	-2647.98827832	-2819.92577764	-2667.441544
			H	-2945.15978657	-2532.77778262	-2647.30118032	-2819.17019464	-2666.711980
			G	-2945.28750757	-2532.89426262	-2647.42407932	-2819.30019264	-2666.837052
		kcal	H	-1848115.66851649	-1589342.05413076	-1661206.57118218	-1769056.00595502	-1673387.031911
			G	-1848195.81465402	-1589415.14643429	-1661283.69146903	-1769137.58093163	-1673465.515776
			G_{rel}	**-0.4**	**1.3**	**2.4**	**2.7**	**3.8**
η2 to TS	Small Basis	kcal	$\Delta G^‡$	**5.1**	**3.6**	**2.8**	**6.7**	**5.0**
	Large Basis		$\Delta G^‡$	**6.2**	**4.6**	**3.8**	**7.1**	**5.9**

				SO$_2$Me	CF$_3$	COMe	CO$_2$Me	CHO
			σ_p	0.72	0.54	0.5	0.45	0.42
Oxidative Addition Product	Small Basis	Hartrees	E	-3120.915108	-2870.056024	-2685.668973	-2760.899051	-2646.344653
			H_{corr}	0.697071	0.662351	0.695401	0.701953	0.665660
			G_{corr}	0.570613	0.537884	0.572841	0.574833	0.546412
			H	-3120.218037	-2869.393673	-2684.973572	-2760.197098	-2645.678993
			G	-3120.344495	-2869.518140	-2685.096132	-2760.324218	-2645.798241
		kcal	H	-1957966.379213	-1800571.714619	-1684846.354121	-1732049.829397	-1660188.633295
			G	-1958045.732806	-1800649.818841	-1684923.261682	-1732129.598402	-1660263.462545
	Large Basis	Hartrees	E	-3121.473028	-2870.639264	-2686.183980	-2761.438771	-2646.849152
			H	-3120.775957	-2869.976913	-2685.488579	-2760.736818	-2646.183492
			G	-3120.902415	-2870.101380	-2685.611139	-2760.863938	-2646.302740
		kcal	H	-1958316.478967	-1800937.703207	-1685169.525767	-1732388.508704	-1660505.211135
			G	-1958395.832560	-1801015.807429	-1685246.433328	-1732468.277708	-1660580.040385
			G_{rel}	**-33.3**	**-32.2**	**-29.7**	**-30.5**	**-31.6**

				COMe	CO$_2$Me	CHO	COCF$_3$	COPh
			σ_p	0.5	0.45	0.42		
Coordination to C=O	Small Basis	Hartrees	E	-2685.648357	-2760.864039	-2646.324142	-2983.374	-2877.397
			H_{corr}	0.694926	0.700567	0.665001	0.673	0.751
			G_{corr}	0.573066	0.576782	0.544992	0.545	0.621
			H	-2684.953431	-2760.163472	-2645.659141	-2982.701	-2876.646
			G	-2685.075291	-2760.287257	-2645.779150	-2982.829	-2876.776
		kcal	H	-1684833.715270	-1732028.728155	-1660176.175984	-1871673.0	-1805122.4
			G	-1684910.183575	-1732106.404415	-1660251.482768	-1871753.6	-1805204.0
	Large Basis	Hartrees	E	-2686.158752	-2761.398943	-2646.824134	-2983.985	-2877.955
			H	-2685.463826	-2760.698376	-2646.159133	-2983.312	-2877.204
			G	-2685.585686	-2760.822161	-2646.279142	-2983.440	-2877.334
		kcal	H	-1685153.992849	-1732364.385495	-1660489.925456	-1872056.3	-1805472.9
			G	-1685230.461154	-1732442.061755	-1660565.232240	-1872136.9	-1805554.5
			G_{rel}	**-13.7**	**-4.3**	**-16.8**	**-26.5**	**-16.7**

				SO_2Me	*NHPh*	*NMe_2*
			σ_p	0.72	-0.56	-0.83
Coordination to Heteroatom	Small Basis	Hartrees	**E**	-3120.854	-2819.384	-2666.939
			**H_{corr}**	0.696	0.758	0.731
			**G_{corr}**	0.567	0.630	0.606
			**H**	-3120.157	-2818.626	-2666.208
			**G**	-3120.286	-2818.754	-2666.334
		kcal	**H**	-1957928.3	-1768714.5	-1673070.9
			**G**	-1958009.1	-1768794.7	-1673149.6
	Large Basis	Hartrees	**E**	-3121.411	-2819.930	-2667.445
			**H**	-3120.714	-2819.172	-2666.714
			**G**	-3120.843	-2819.300	-2666.839
		kcal	**H**	-1958277.9	-1769057.2	-1673388.2
			**G**	-1958358.7	-1769137.4	-1673466.9
			**G_{rel}**	**3.9**	**2.9**	**2.4**

COMe ring walking			**e2-CO**	**e2-CO to meta/para**	**meta/para**	**meta/para to pre-OA**	**pre-OA**
Small Basis	Hartrees	E	-2685.648292	-2685.631633	-2685.635866	-2685.624471	-2685.634915
		H_{corr}	0.69488	0.693142	0.694057	0.693057	0.694285
		G_{corr}	0.57316	0.574201	0.571631	0.572996	0.571746
		H	-2684.953411670	-2684.938491000	-2684.941809030	-2684.931414440	-2684.940629720
		G	-2685.075131670	-2685.057432000	-2685.064235030	-2685.051475440	-2685.063168720
	kcal	H	-1684833.703071550	-1684824.340209760	-1684826.422305020	-1684819.899601320	-1684825.682276830
		G	-1684910.083524720	-1684898.976814110	-1684903.245779890	-1684895.239016280	-1684902.576660260
		G_{rel}	**-16.9**	**-5.8**	**-10.1**	**-2.0**	**-9.4**
Large Basis	Hartrees	E	-2686.158687	-2686.144624	-2686.149403	-2686.137723	-2686.148883
		H	-2685.463806550	-2685.451481560	-2685.455345990	-2685.444666020	-2685.454597940
		G	-2685.585526550	-2685.570422560	-2685.577771990	-2685.564727020	-2685.577136940
	kcal	H	-1685153.980694230	-1685146.246646240	-1685148.671612670	-1685141.969830320	-1685148.202204210
		G	-1685230.361147	-1685220.883251	-1685225.495088	-1685217.309245	-1685225.096588
		G_{rel}	**-13.6**	**-4.1**	**-8.8**	**-0.6**	**-8.4**

CHO ring walking			e2-CO	e2-CO to meta/para	meta/para	meta/para to pre-OA	pre-OA
Small Basis	Hartrees	E	-2646.323997	-2646.307499	-2646.310793	-2646.297353	-2646.308946
		H_{corr}	0.664834	0.663689	0.664837	0.663231	0.664514
		G_{corr}	0.544333	0.547183	0.545867	0.54579	0.545317
		H	-2645.659163460	-2645.643809590	-2645.645955790	-2645.634122360	-2645.644431950
		G	-2645.779664460	-2645.760315590	-2645.764925790	-2645.751563360	-2645.763628950
	kcal	H	-1660176.190046060	-1660166.555347180	-1660167.902108010	-1660160.476518580	-1660166.945883970
		G	-1660251.805565190	-1660239.663965950	-1660242.556910130	-1660234.171858710	-1660241.743130750
		G_{rel}	**-20.2**	**-8.1**	**-11.0**	**-2.6**	**-10.2**
Large Basis	Hartrees	E	-2646.823982	-2646.809849	-2646.813771	-2646.800291	-2646.813092
		H	-2646.159147910	-2646.146160420	-2646.148933640	-2646.137059510	-2646.148577530
		G	-2646.279648910	-2646.262666420	-2646.267903640	-2646.254500510	-2646.267774530
	kcal	H	-1660489.935025290	-1660481.785252270	-1660483.525474100	-1660476.074345030	-1660483.302011700
		G	-1660565.550544	-1660554.893871	-1660558.180276	-1660549.769685	-1660558.099258
		G_{rel}	**-17.1**	**-6.4**	**-9.7**	**-1.3**	**-9.7**

COMe ring walking (ortho)			ArBr	e2-CO	e2-CO to pre-OA	pre-OA	OATS	Product
Small Basis	Hartrees	E	-397.4757668	-2685.635267	-2685.616497	-2685.630319	-2685.622045	-2685.6679
		H_{corr}	0.138272	0.694611	0.693318	0.694288	0.693567	0.696046
		G_{corr}	0.091899	0.572708	0.576046	0.573221	0.571945	0.572541
		H	-397.3374948	-2684.940656	-2684.923179	-2684.936031	-2684.928478	-2684.971854
		G	-397.3838678	-2685.062559	-2685.040451	-2685.057098	-2685.0501	-2685.095359
	kcal	H	-249333.0423	-1684825.699	-1684814.732	-1684822.796	-1684818.057	-1684845.276
		G	-249362.1418	-1684902.194	-1684888.321	-1684898.767	-1684894.376	-1684922.776
		G_{rel}		-15.3	-1.4	-11.9	-7.5	-35.9
Large Basis	Hartrees	E	-397.5867525	-2686.146268	-2686.134689	-2686.141308	-2686.130224	-2686.175759
		H	-397.4484805	-2685.451657	-2685.441371	-2685.44702	-2685.436657	-2685.479713
		G	-397.4948535	-2685.57356	-2685.558643	-2685.568087	-2685.558279	-2685.603218
	kcal	H	-249402.6869	-1685146.357	-1685139.902	-1685143.447	-1685136.944	-1685163.962
		G	-249431.7864	-1685222.852	-1685213.492	-1685219.418	-1685213.263	-1685241.462
		G_{rel}		-12.4	-3.0	-9.0	-2.8	-31.0

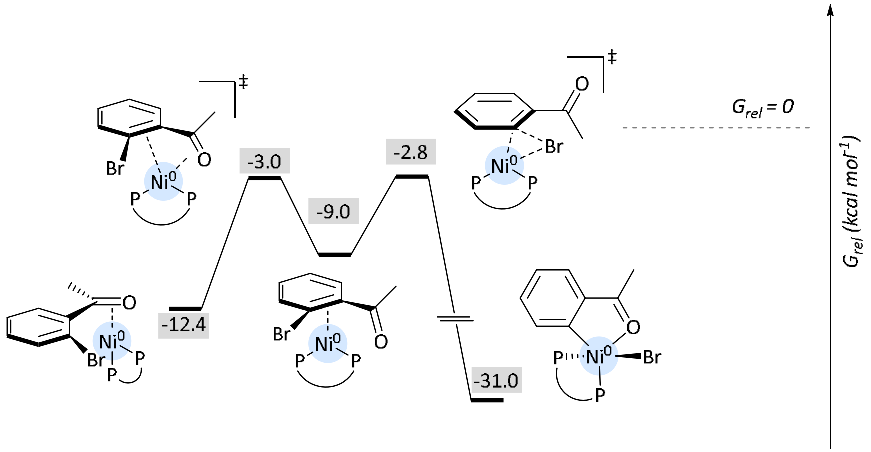

NMR SPECTRA

4-methyl-4'-(trifluoromethyl)-1,1'-biphenyl

^{1}H NMR (400 MHz, $CDCl_3$)

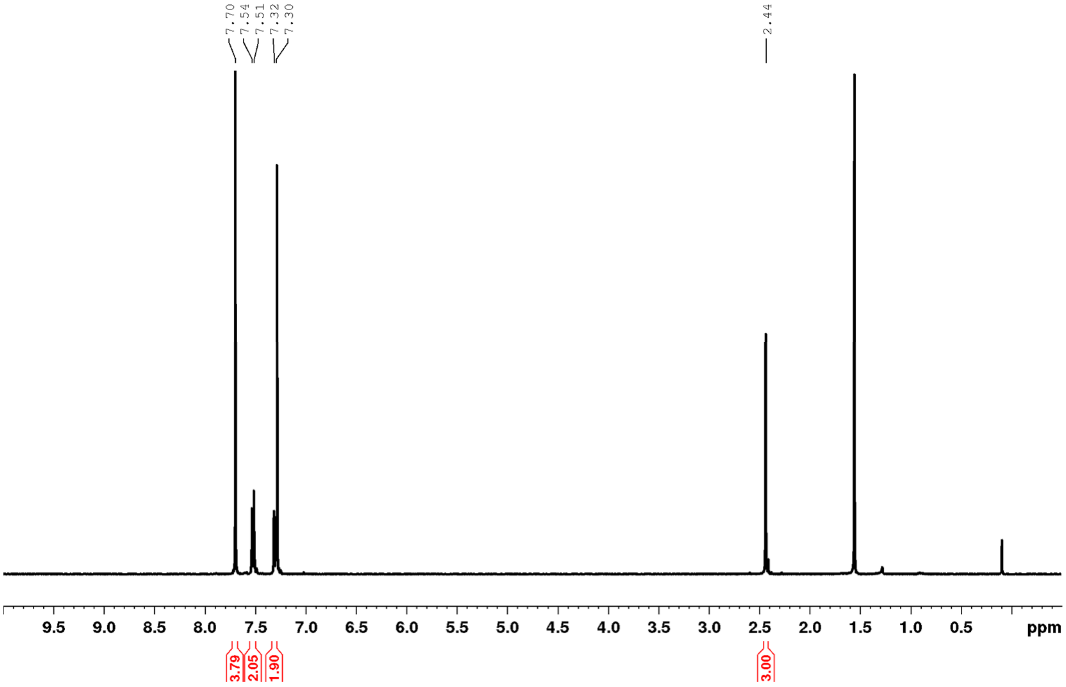

$^{13}C\{^1H\}$ NMR (101 MHz, $CDCl_3$)

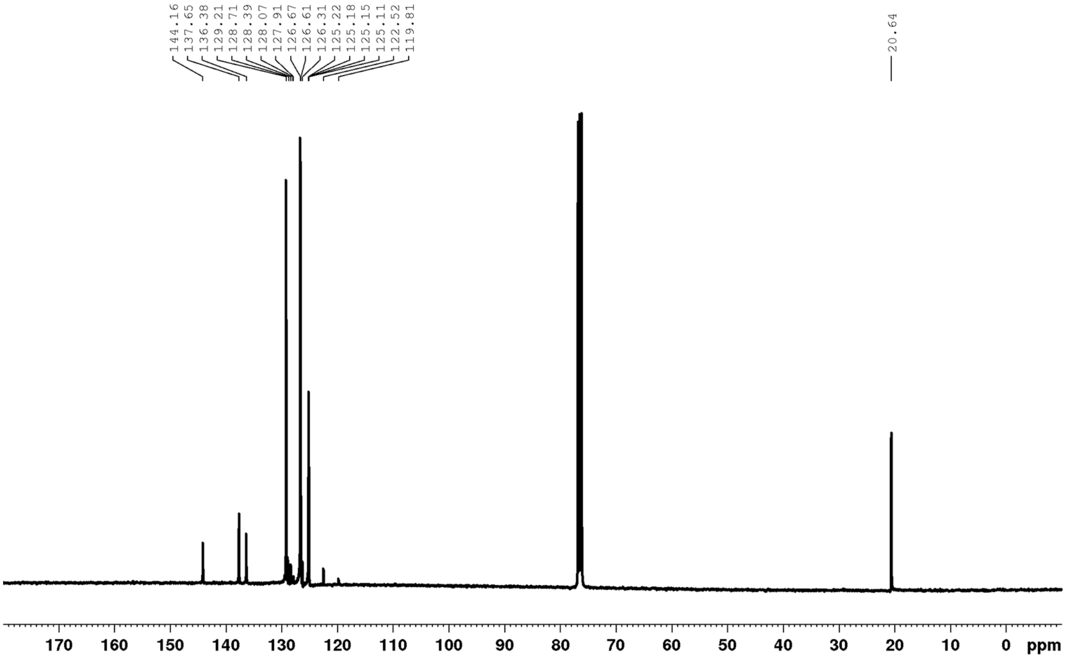

^{19}F NMR (376 MHz, $CDCl_3$)

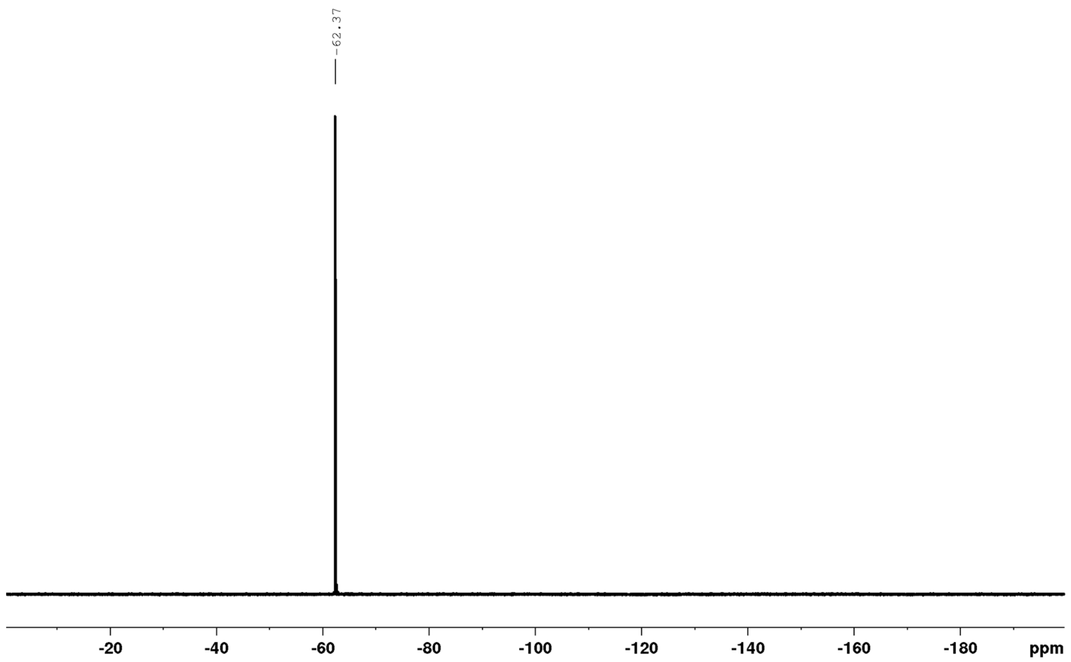

Methyl 4’-methyl-[1,1’-biphenyl]-4-carboxylate

^{1}H NMR (400 MHz, $CDCl_3$)

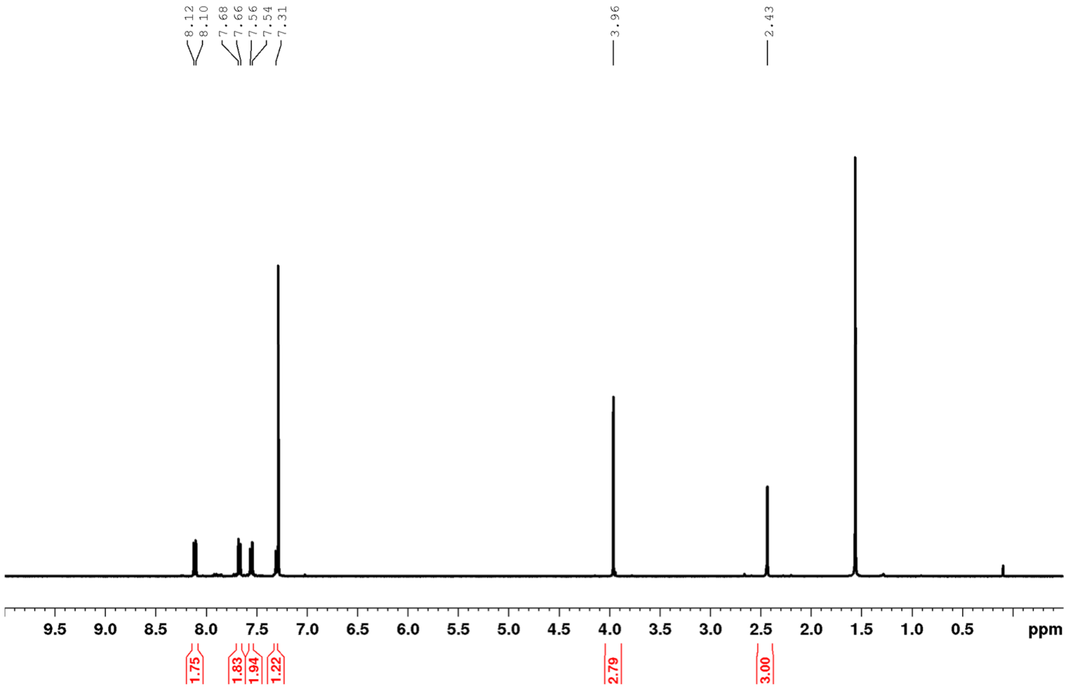

$^{13}C\{^{1}H\}$ NMR (101 MHz, $CDCl_3$)

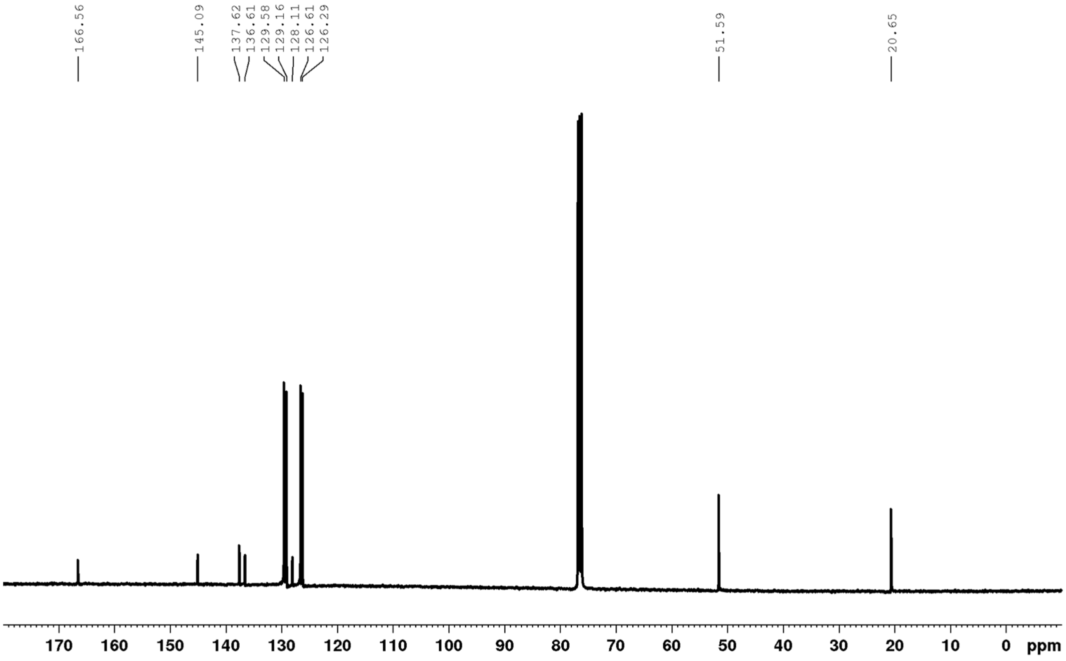

***N,N*,4’-trimethyl-[1,1’-biphenyl]-4-amine**

^{1}H NMR (400 MHz, $CDCl_3$)

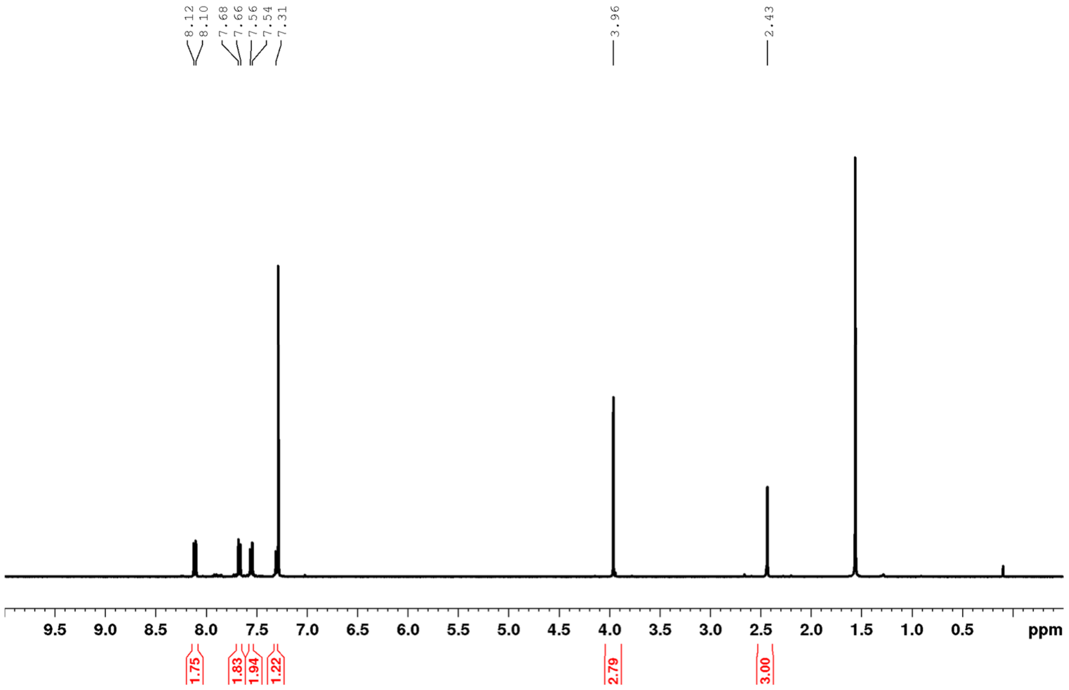

$^{13}C\{^{1}H\}$ NMR (101 MHz, $CDCl_3$)

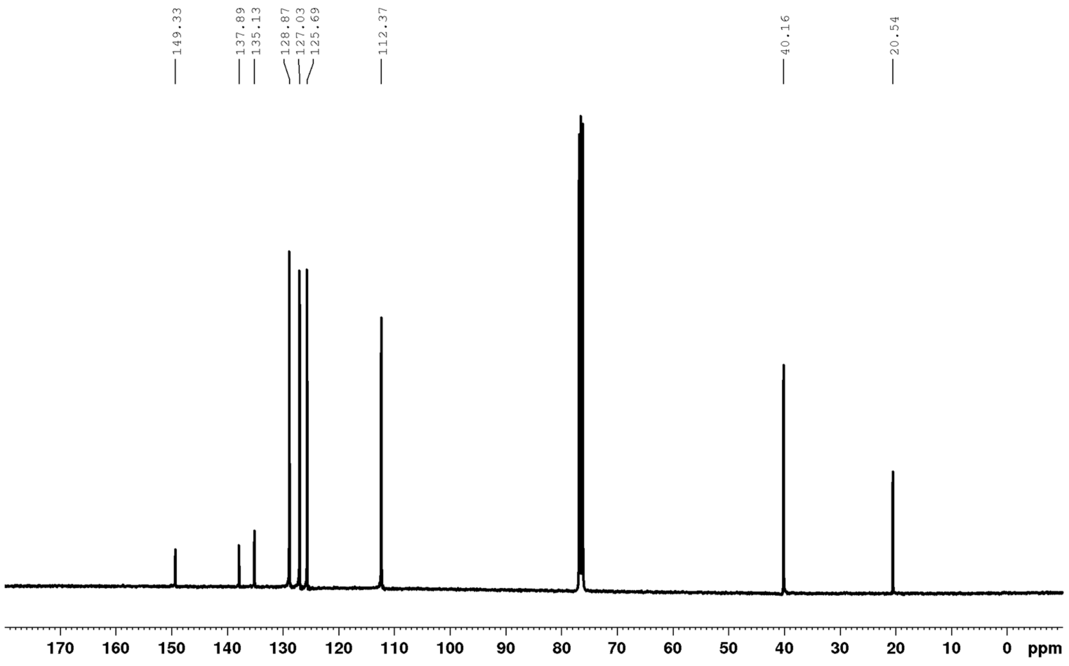

4-methoxy-4'-methyl-1,1'-biphenyl

^{1}H NMR (400 MHz, $CDCl_3$)

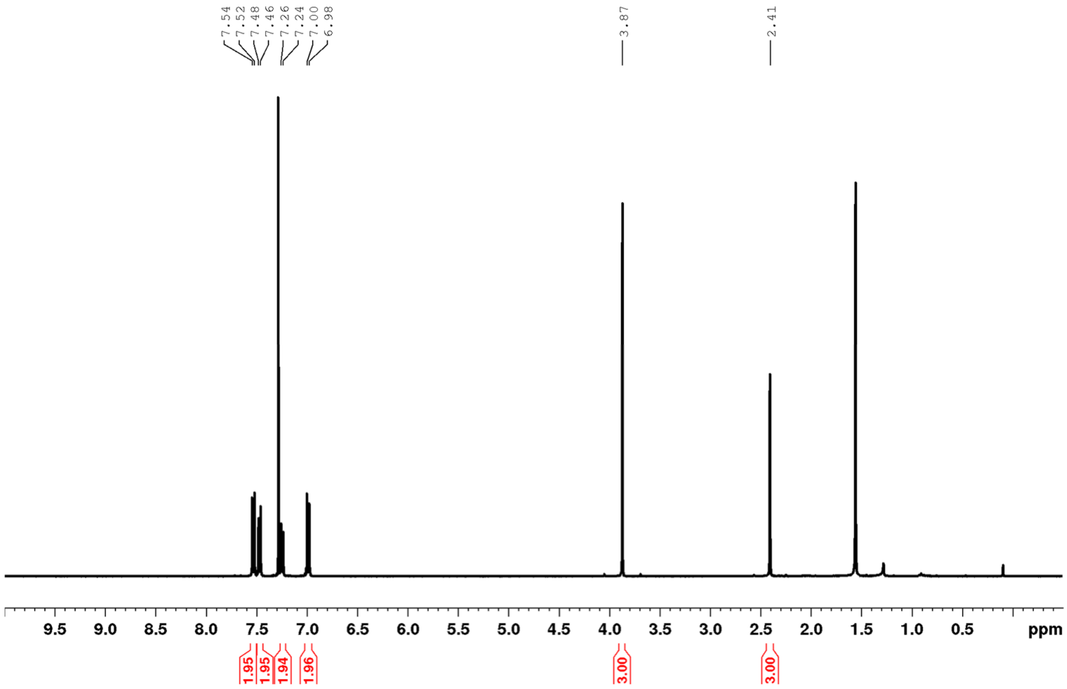

^{13}C{^{1}H} NMR (101 MHz, $CDCl_3$)

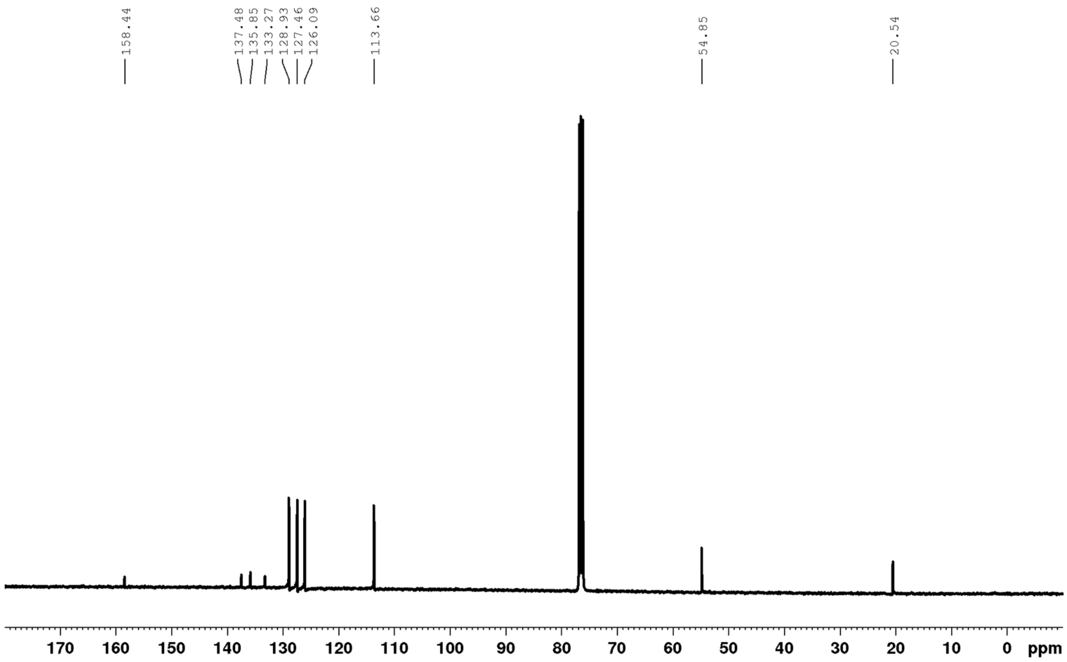

4’-methyl-[1,1’-biphenyl]-4-carbaldehyde

^{1}H NMR (400 MHz, $CDCl_3$)

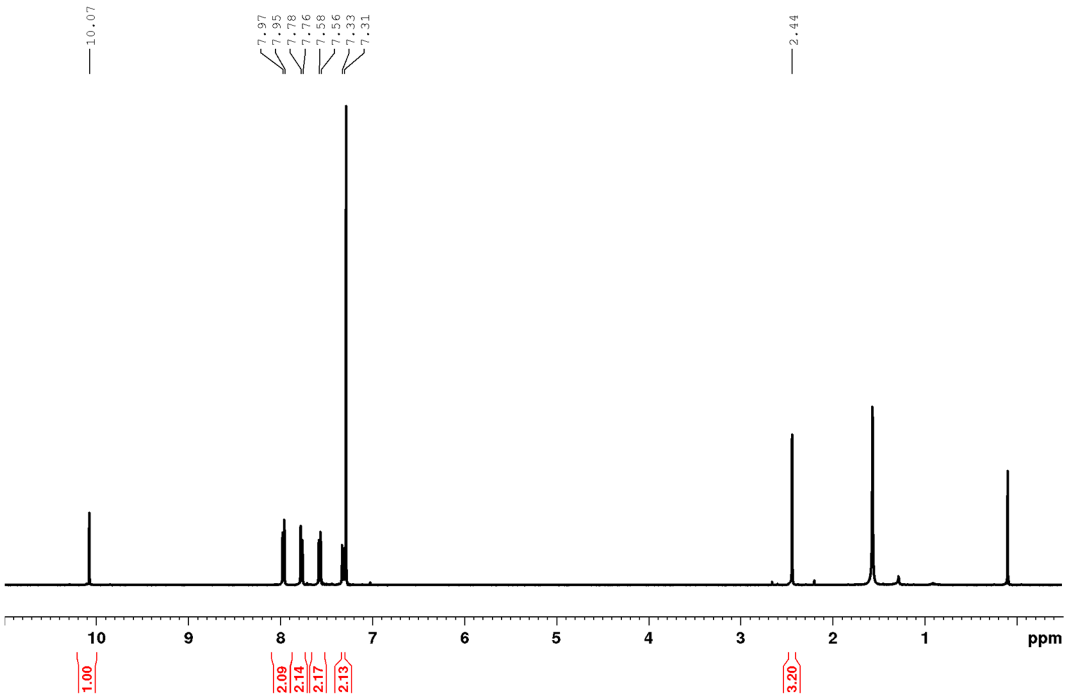

$^{13}C\{^{1}H\}$ NMR (101 MHz, $CDCl_3$)

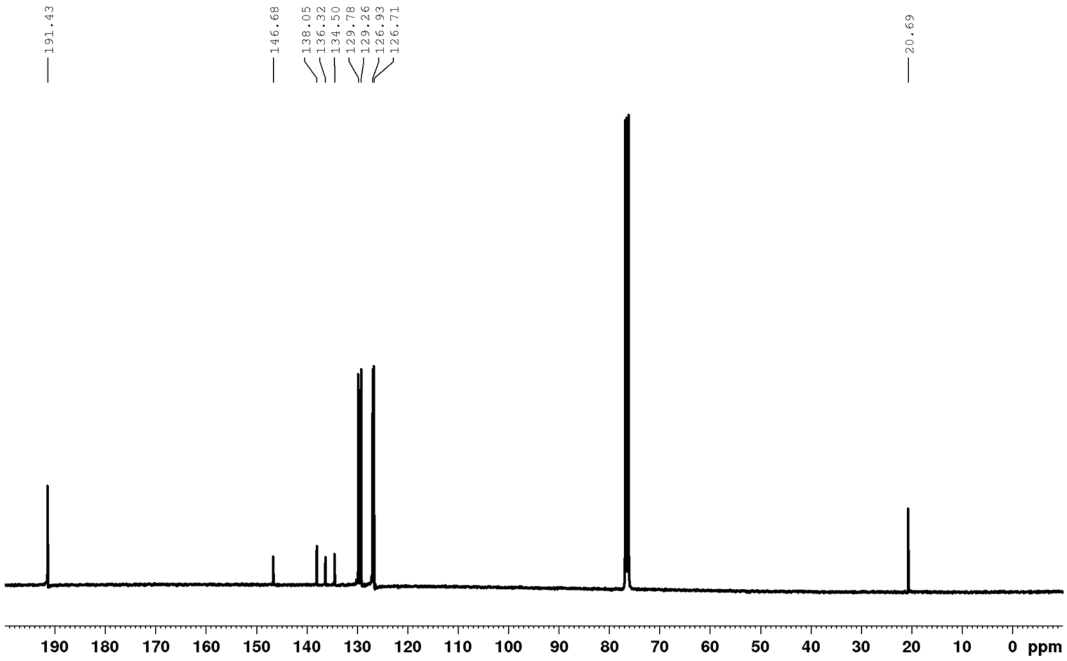

1-(4'-methyl-[1,1'-biphenyl]-4-yl)ethan-1-one

^{1}H NMR (400 MHz, $CDCl_3$)

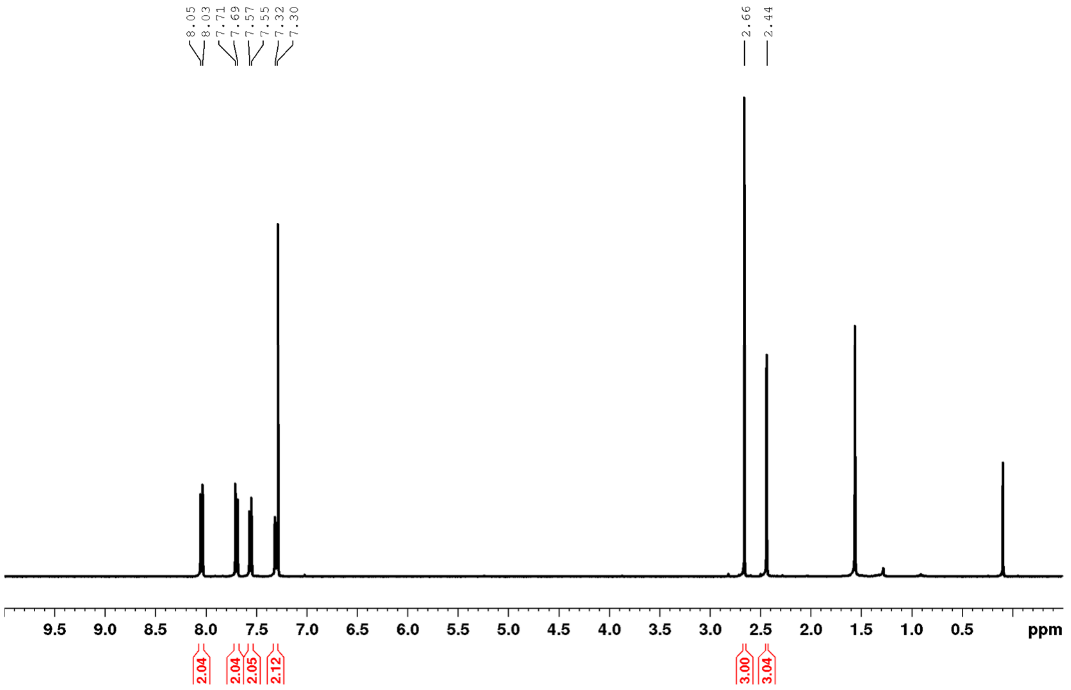

$^{13}C\{^{1}H\}$ NMR (101 MHz, $CDCl_3$)

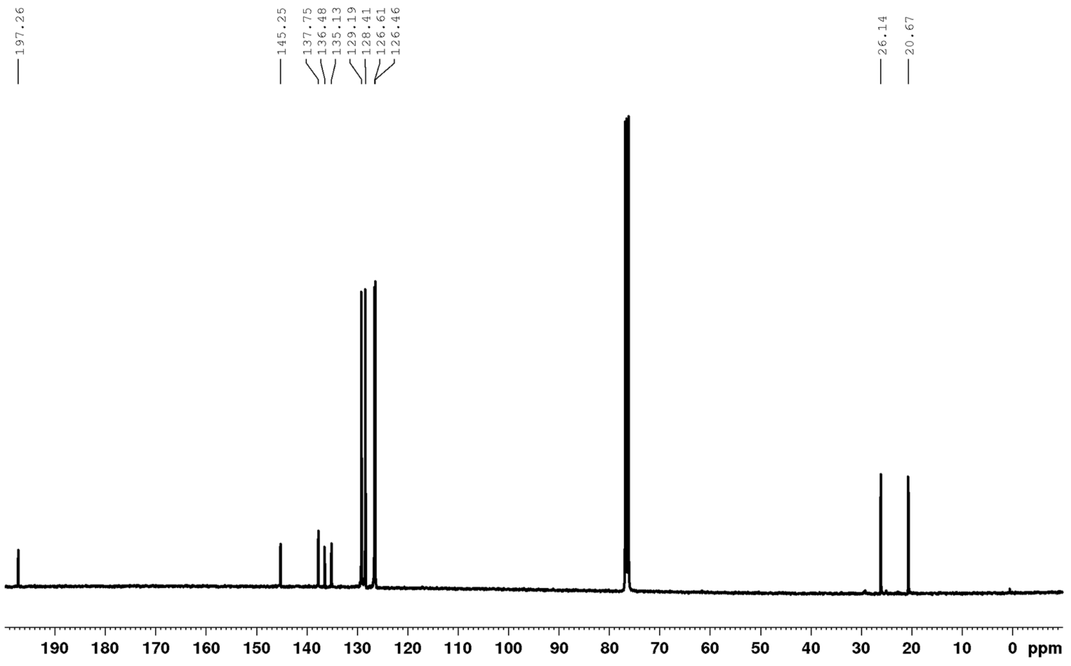

(4’-methyl-[1,1’-biphenyl]-4-yl)(phenyl)methanone

^{1}H NMR (400 MHz, $CDCl_3$)

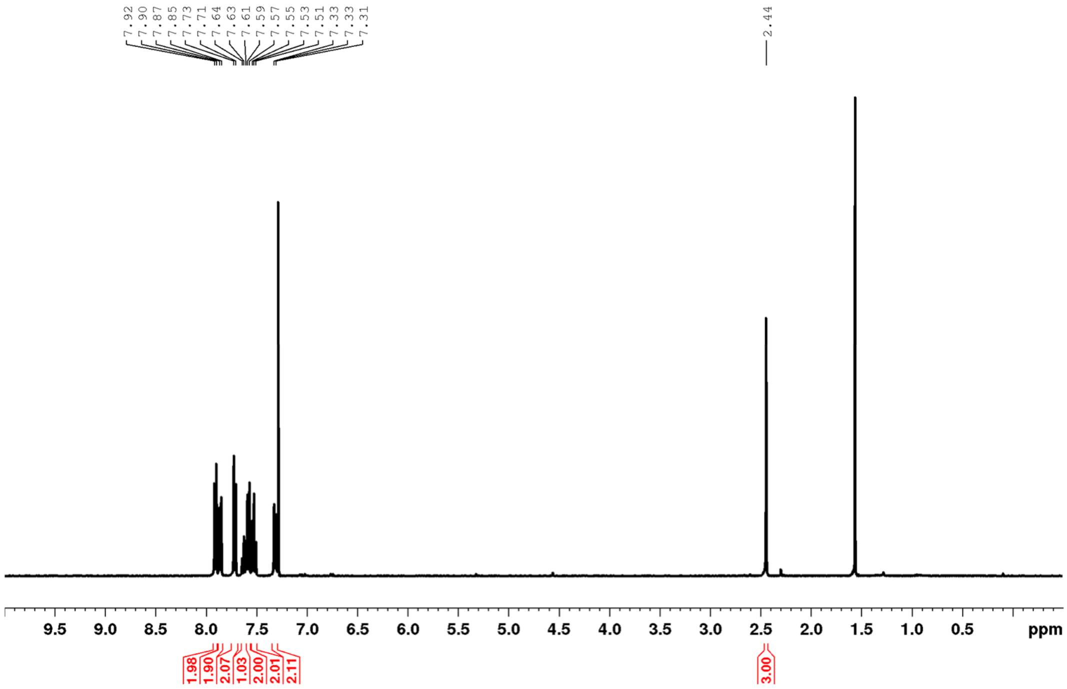

$^{13}C\{^{1}H\}$ NMR (101 MHz, $CDCl_3$)

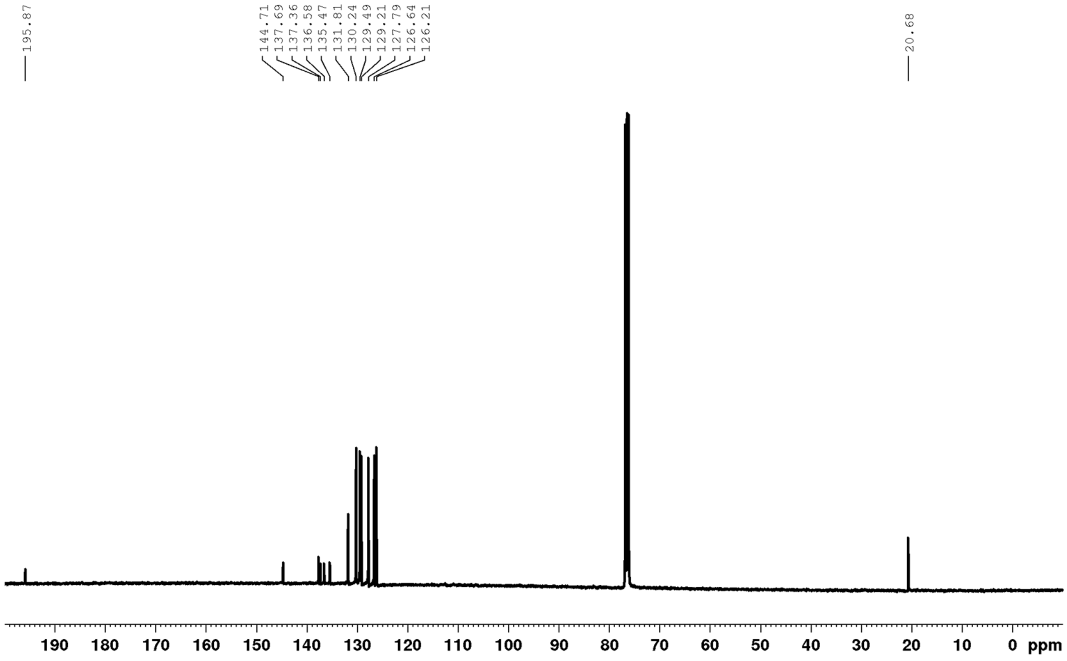

4-fluoro-4'-methyl-1,1'-biphenyl

^{1}H NMR (400 MHz, $CDCl_3$)

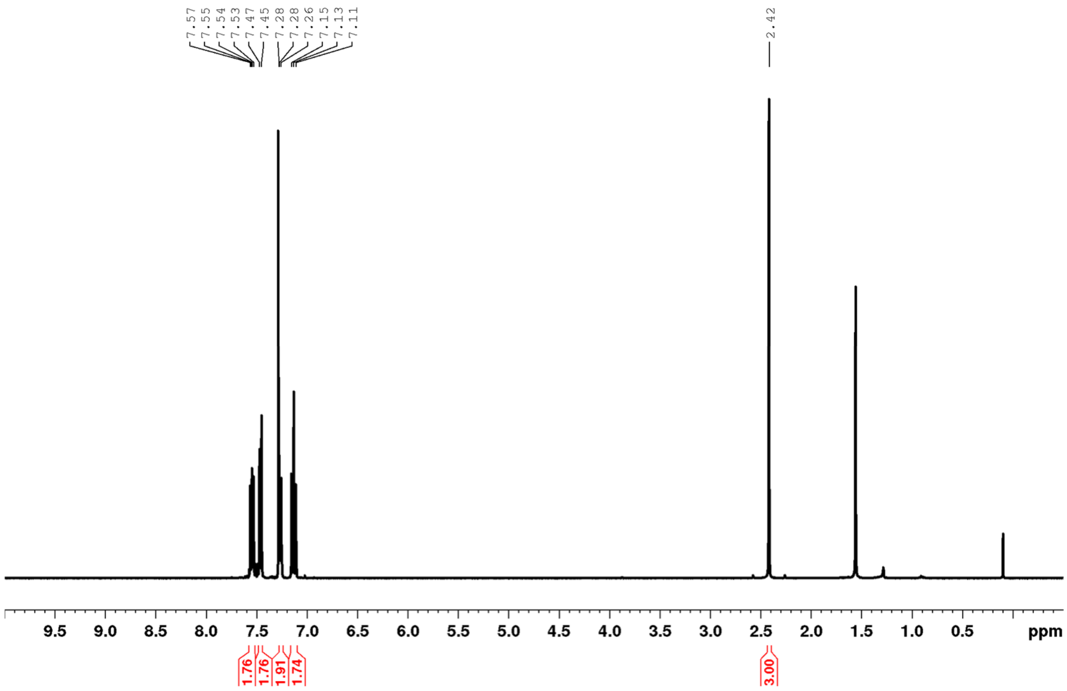

$^{13}C\{^{1}H\}$ NMR (101 MHz, $CDCl_3$)

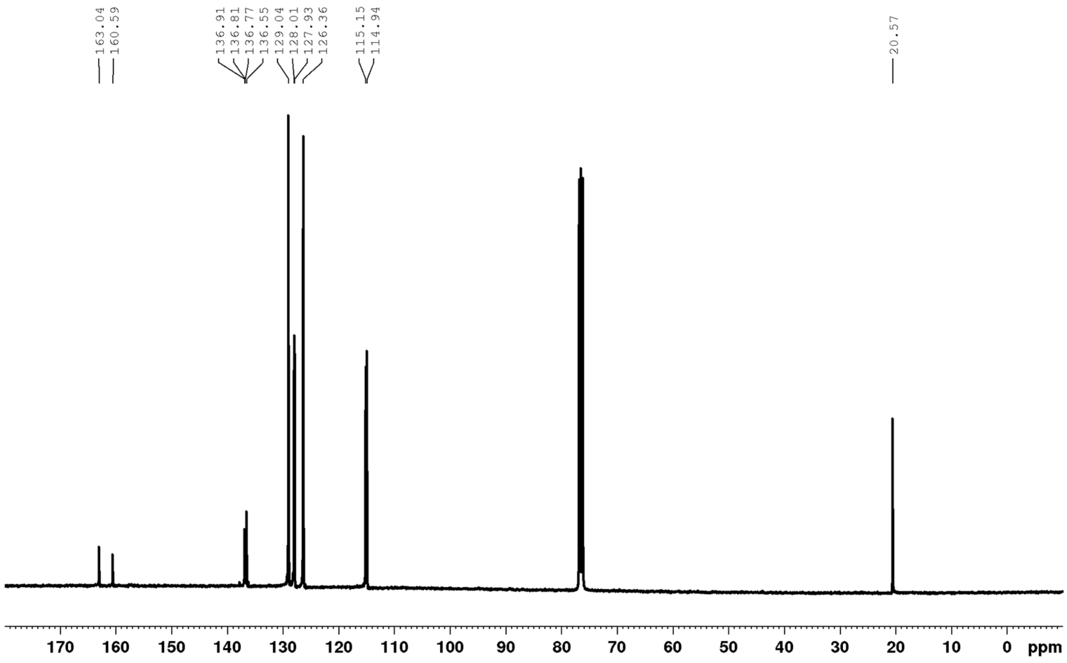

^{19}F NMR (376 MHz, $CDCl_3$)

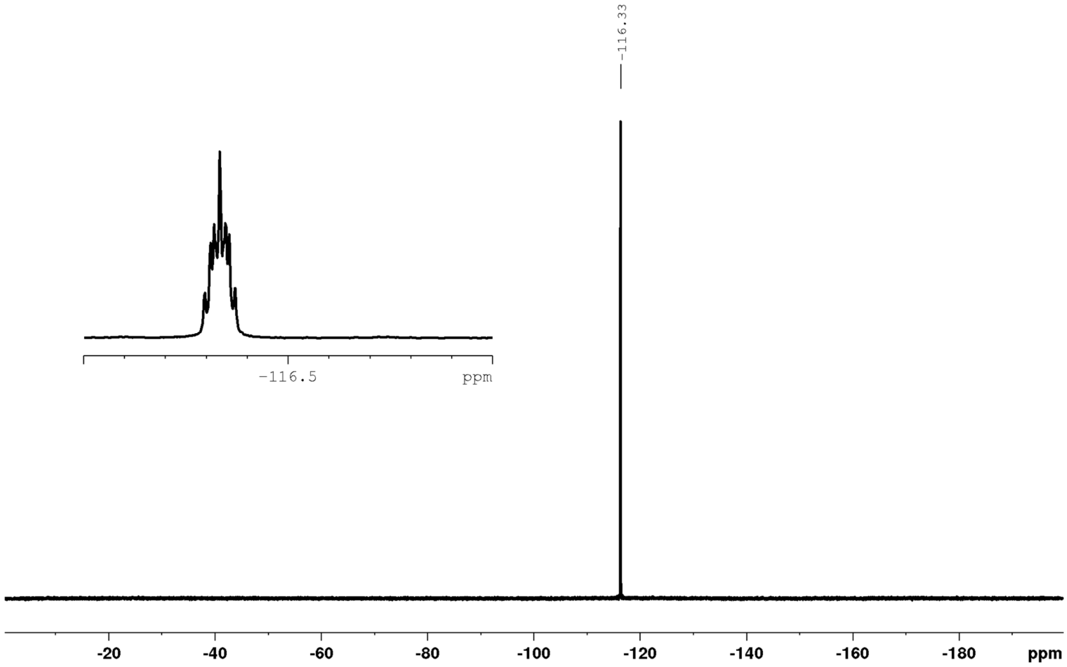

$^{19}F\{^1H\}$ NMR (376 MHz, $CDCl_3$)

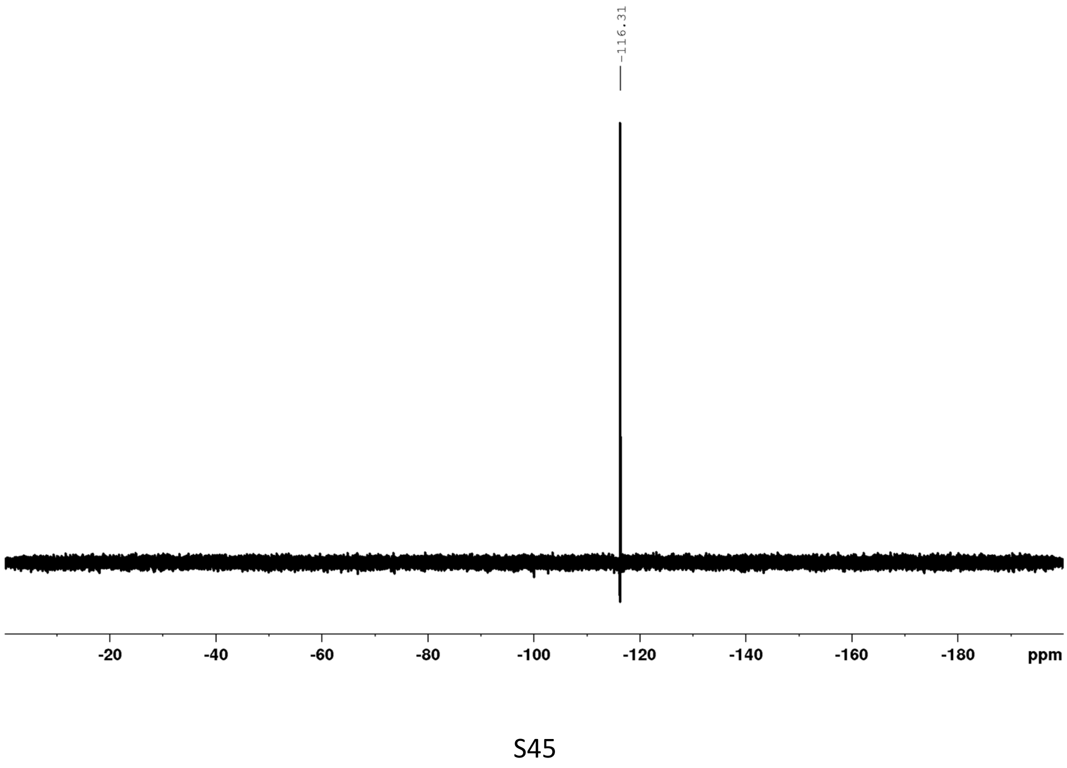

4-(difluoromethoxy)-4'-methyl-1,1'-biphenyl

^{1}H NMR (400 MHz, $CDCl_3$)

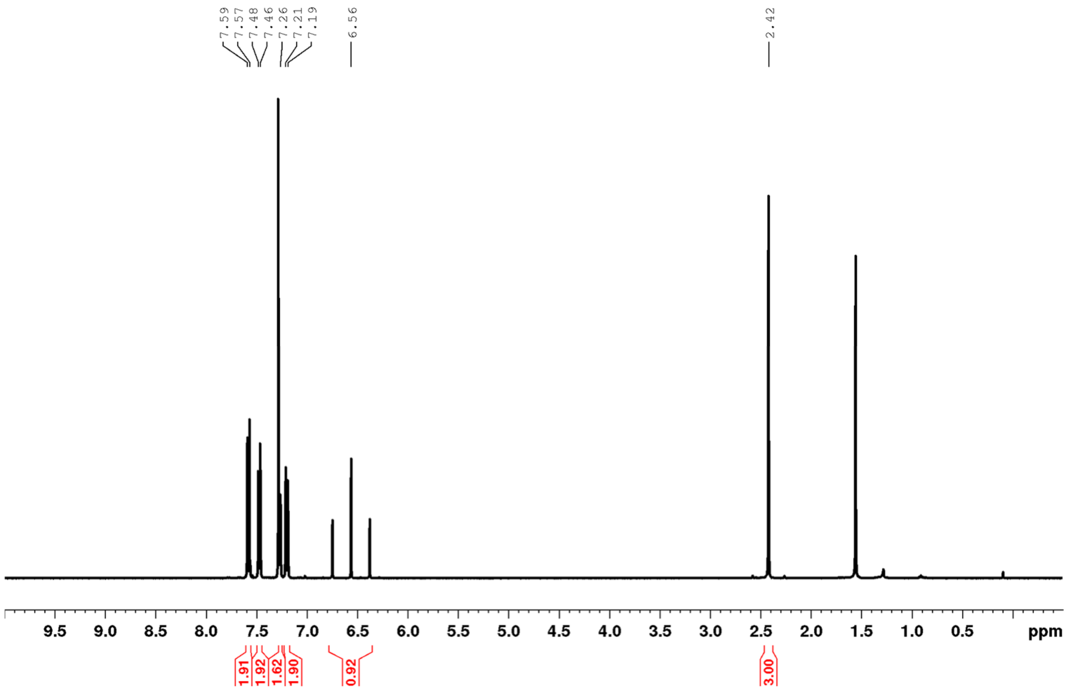

$^{13}C\{^{1}H\}$ NMR (101 MHz, $CDCl_3$)

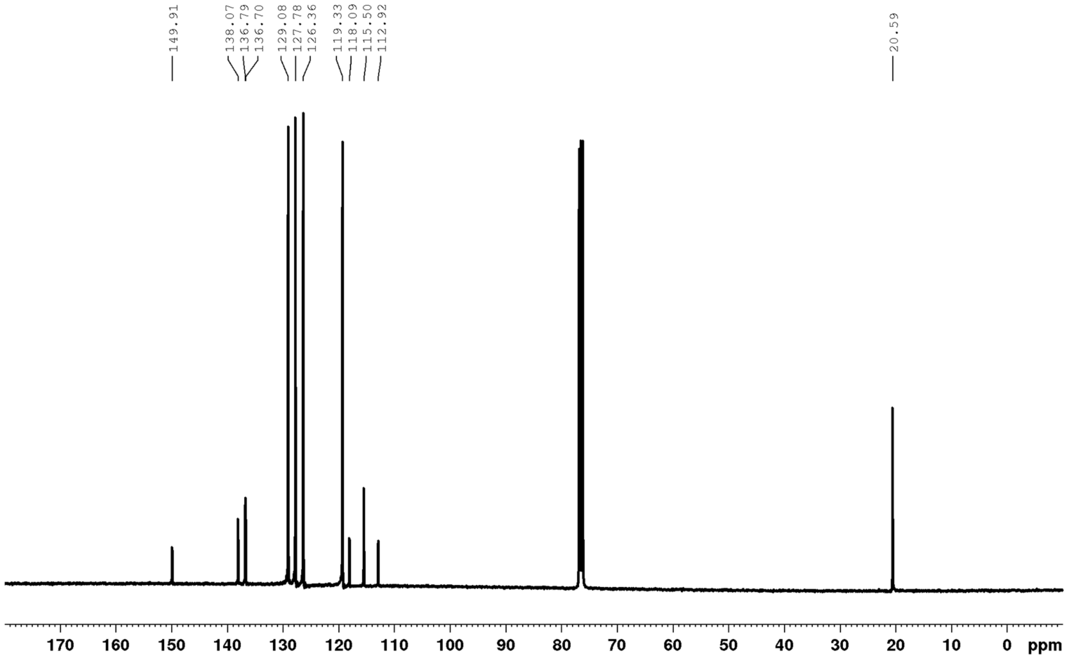

^{19}F NMR (376 MHz, $CDCl_3$)

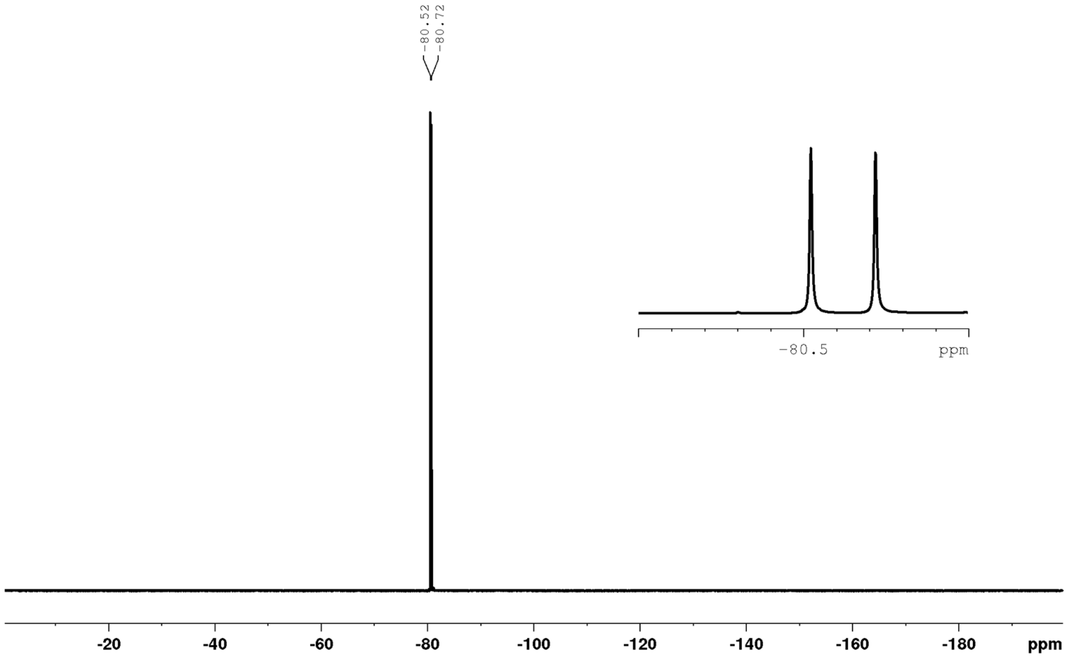

4-methyl-4'-(trifluoromethoxy)-1,1'-biphenyl

^{1}H NMR (400 MHz, $CDCl_3$)

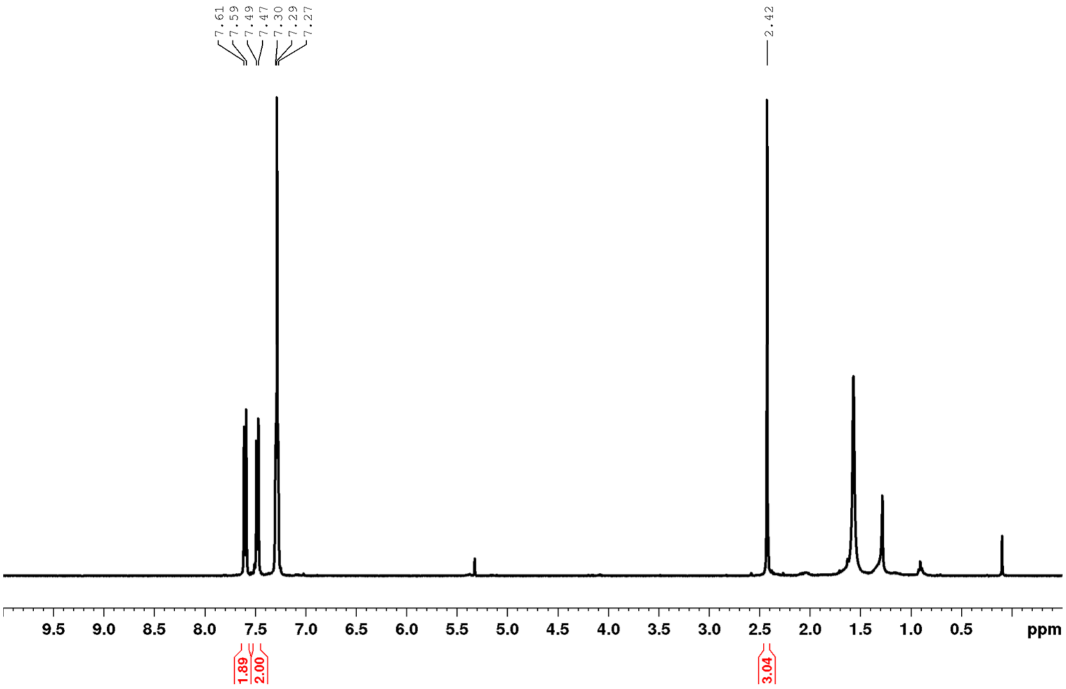

$^{13}C\{^{1}H\}$ NMR (101 MHz, $CDCl_3$)

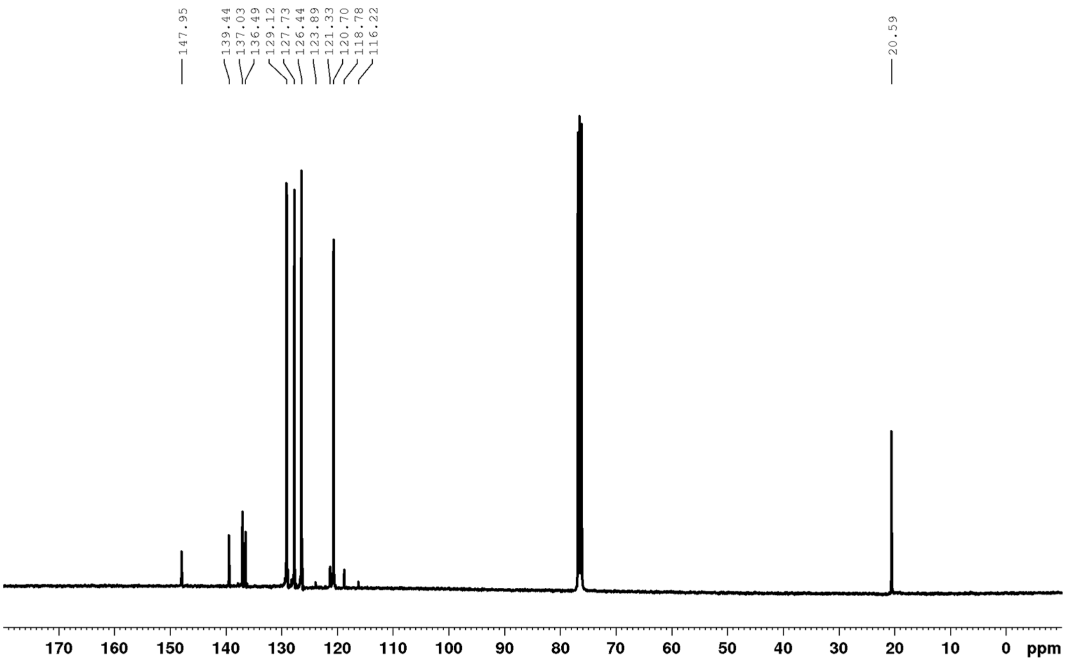

^{19}F NMR (376 MHz, $CDCl_3$)

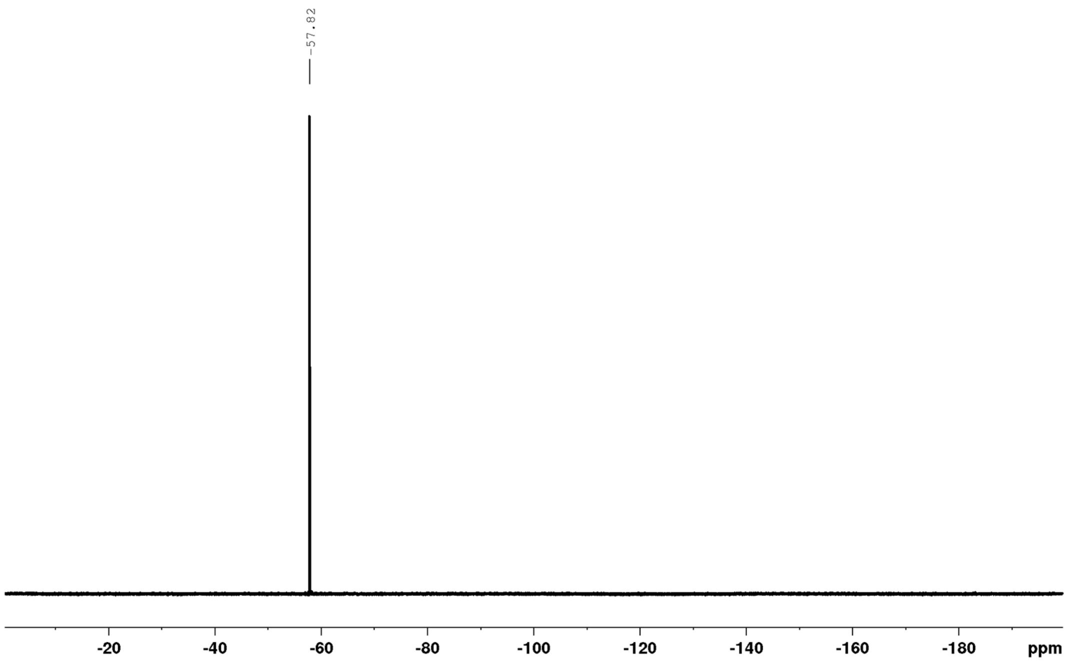

4’-methyl-4-isopropyl-1,1’-biphenyl

^{1}H NMR (400 MHz, $CDCl_3$)

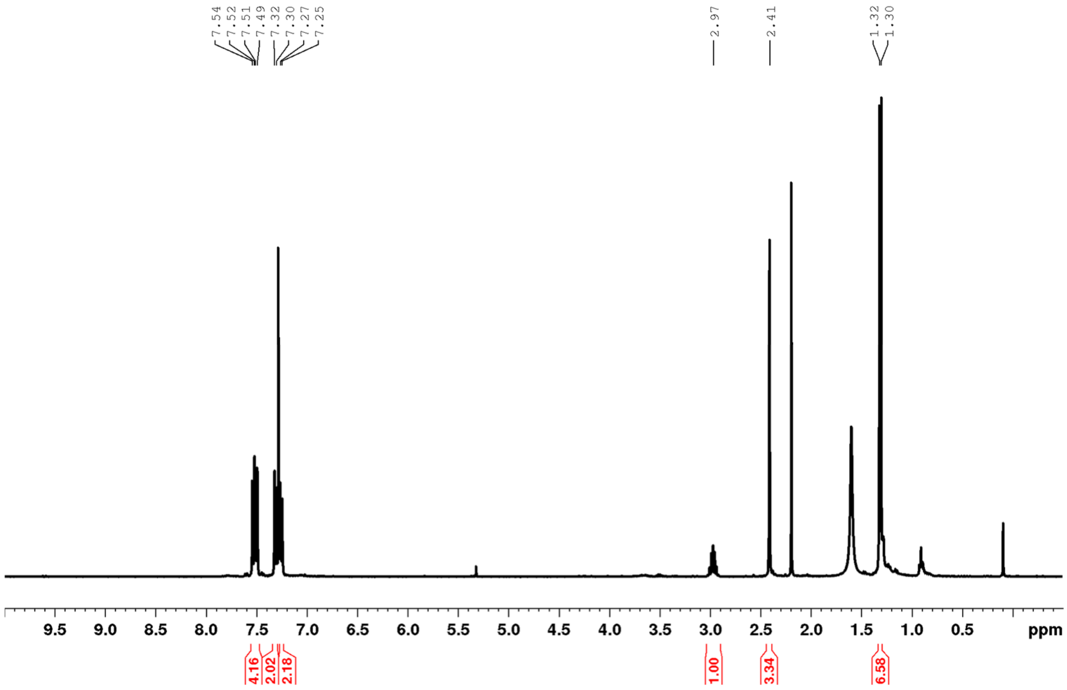

$^{13}C\{^{1}H\}$ NMR (101 MHz, $CDCl_3$)

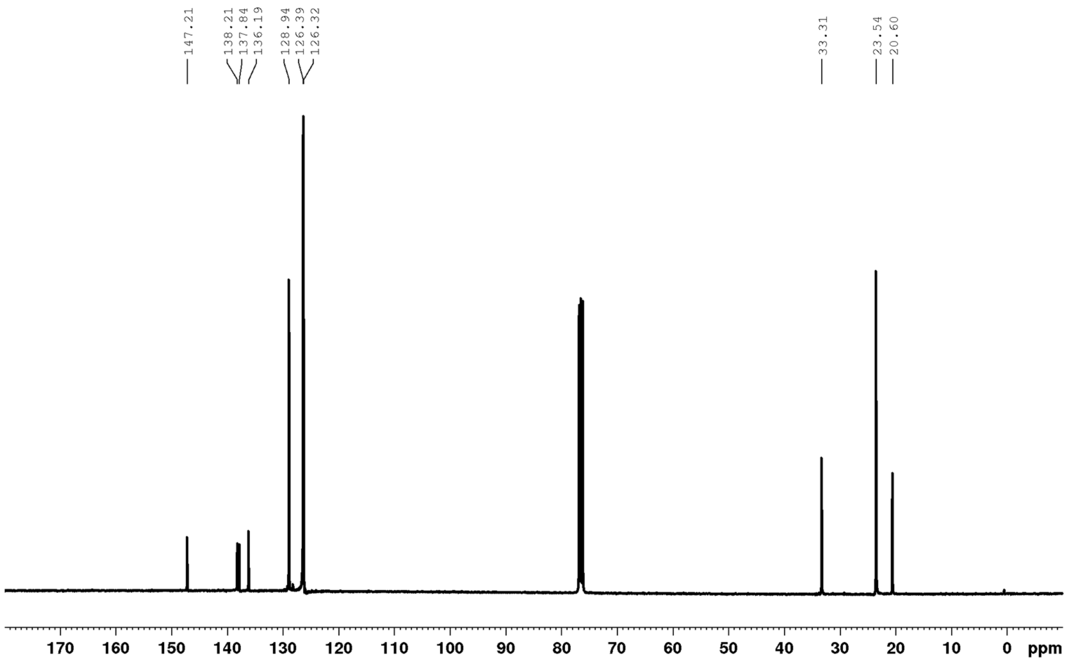

N,N-diethyl-4'-methyl-[1,1'-biphenyl]-4-amine

^{1}H NMR (400 MHz, $CDCl_3$)

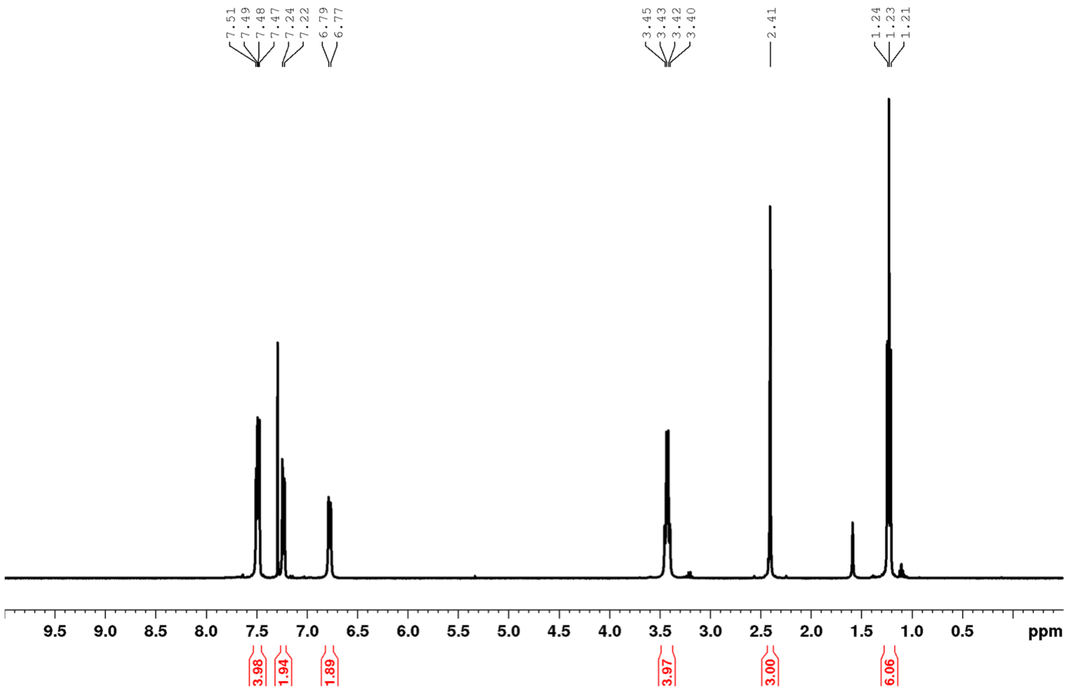

$^{13}C\{^{1}H\}$ NMR (101 MHz, $CDCl_3$)

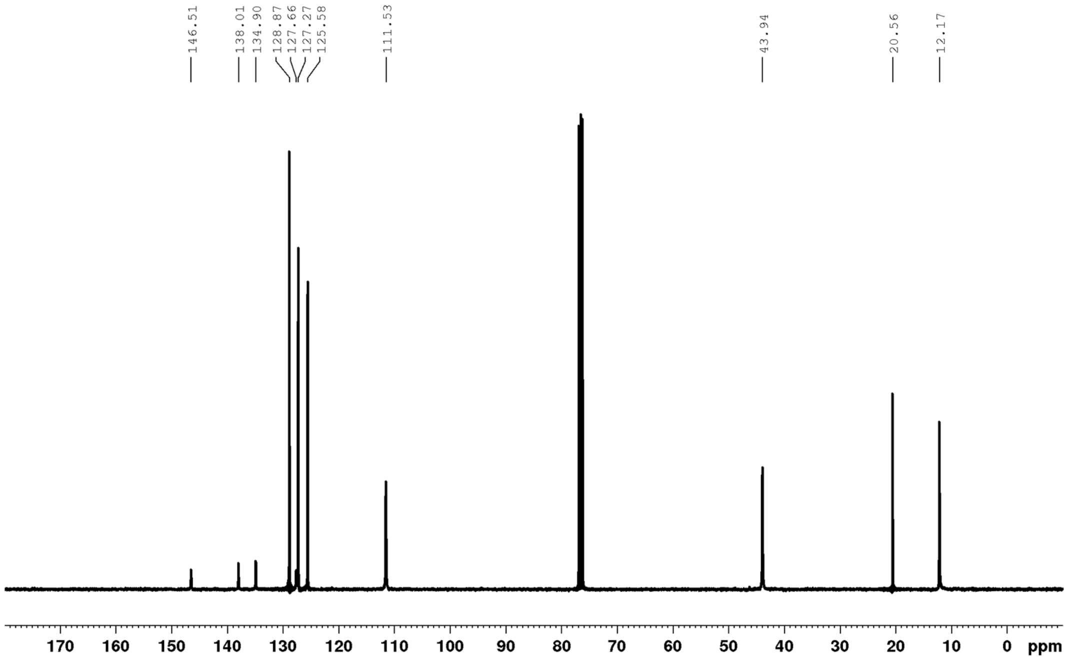

2,2,2-trifluoro-1-(4'-methyl-[1,1'-biphenyl]-4-yl)ethan-1-one

^{1}H NMR (400 MHz, $CDCl_3$)

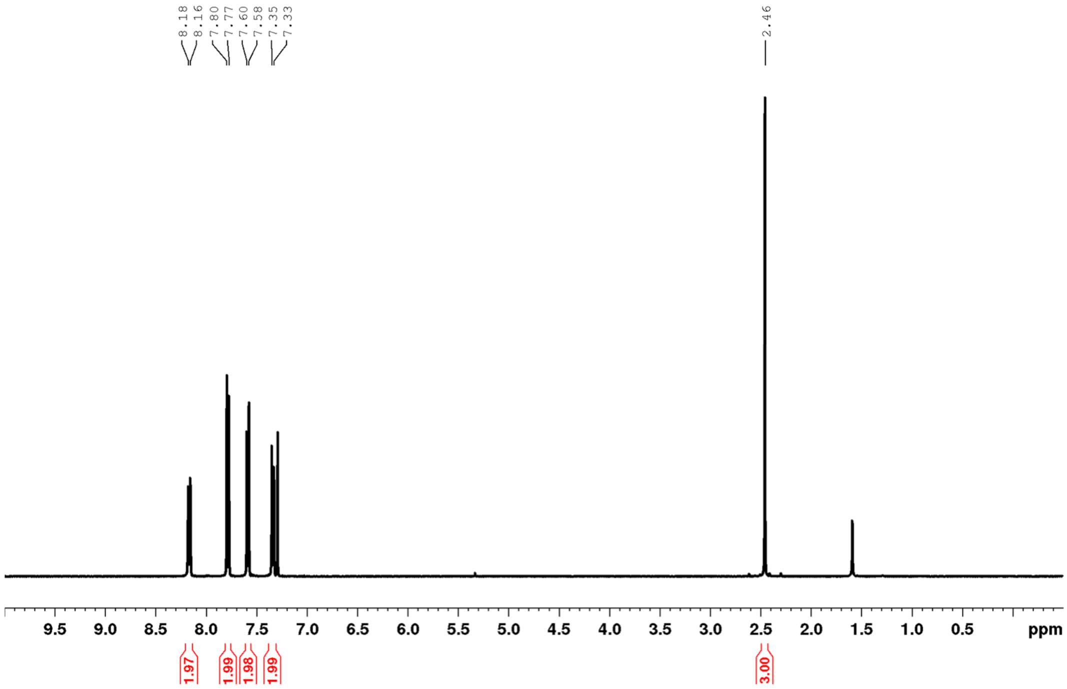

^{13}C{^{1}H} NMR (101 MHz, $CDCl_3$)

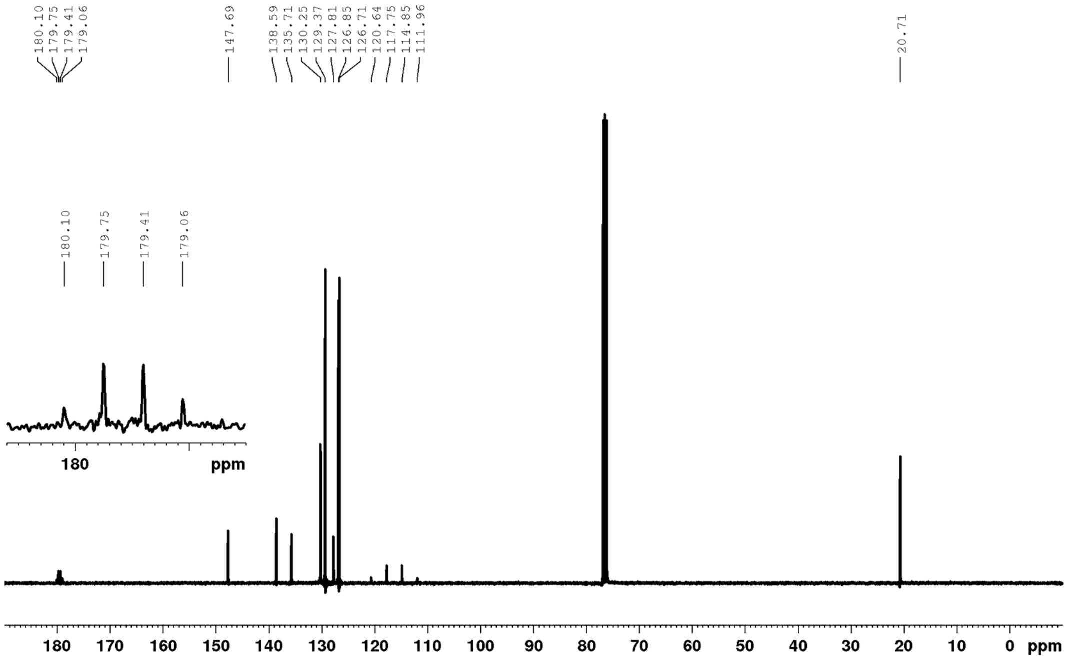

^{19}F NMR (376 MHz, $CDCl_3$)

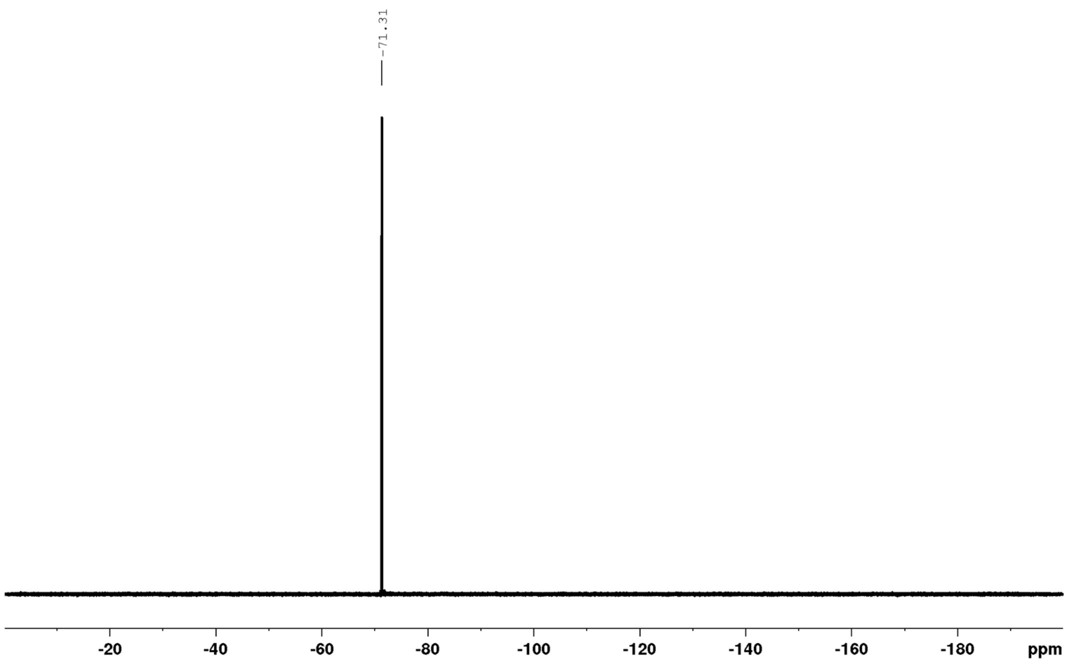

4'-methyl-N-phenyl-[1,1'-biphenyl]-4-amine

^{1}H NMR (400 MHz, $CDCl_3$)

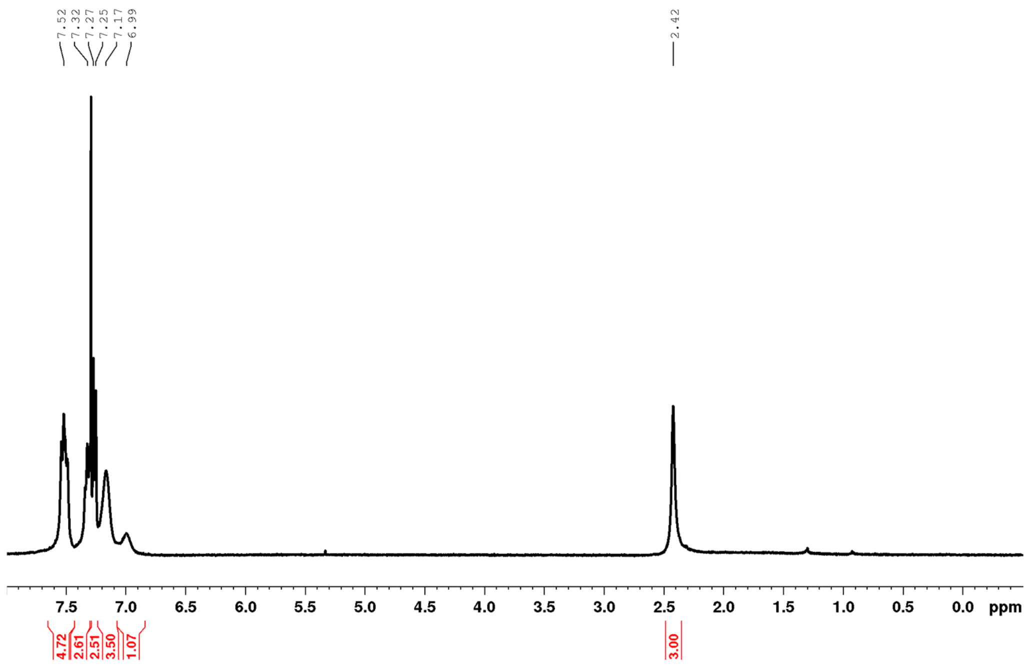

^{13}C{^{1}H} NMR (101 MHz, $CDCl_3$)

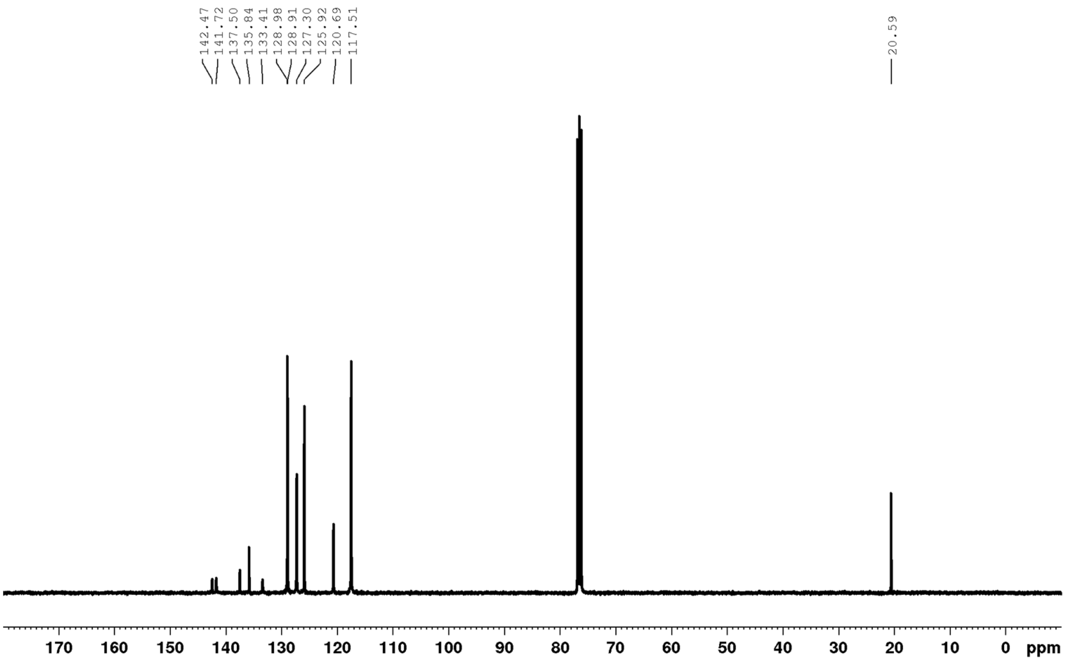

4-methyl-4'-(methylsulfonyl)-1,1'-biphenyl

^{1}H NMR (400 MHz, $CDCl_3$)

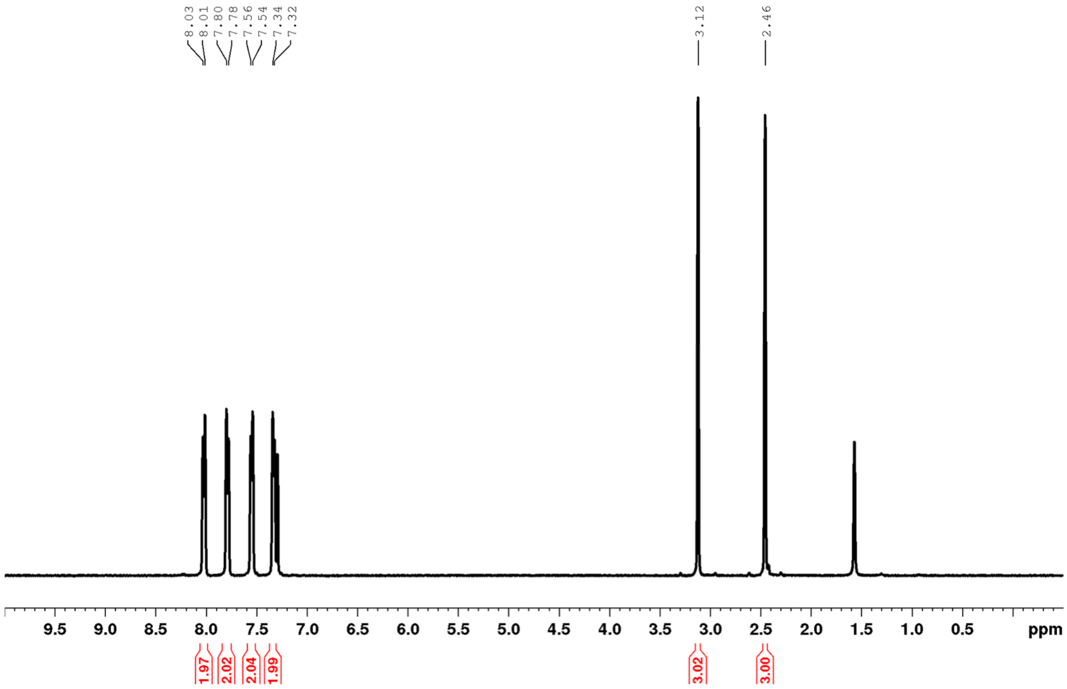

$^{13}C\{^{1}H\}$ NMR (101 MHz, $CDCl_3$)

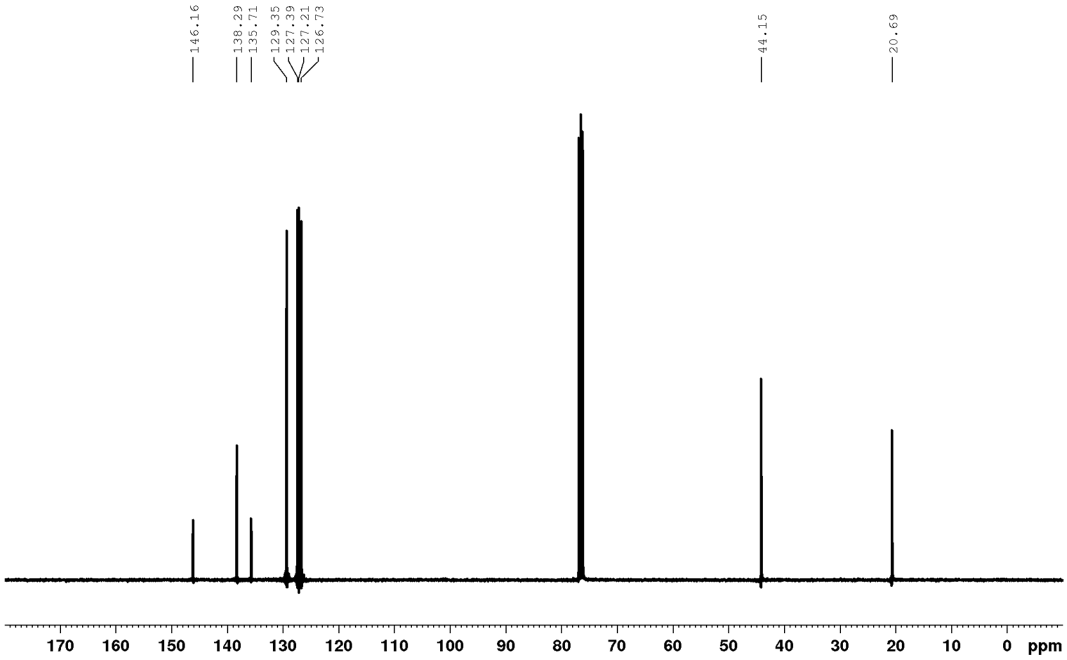

1,3-bis(4-chlorophenyl)propan-1-one

^{1}H NMR (400 MHz, $CDCl_3$)

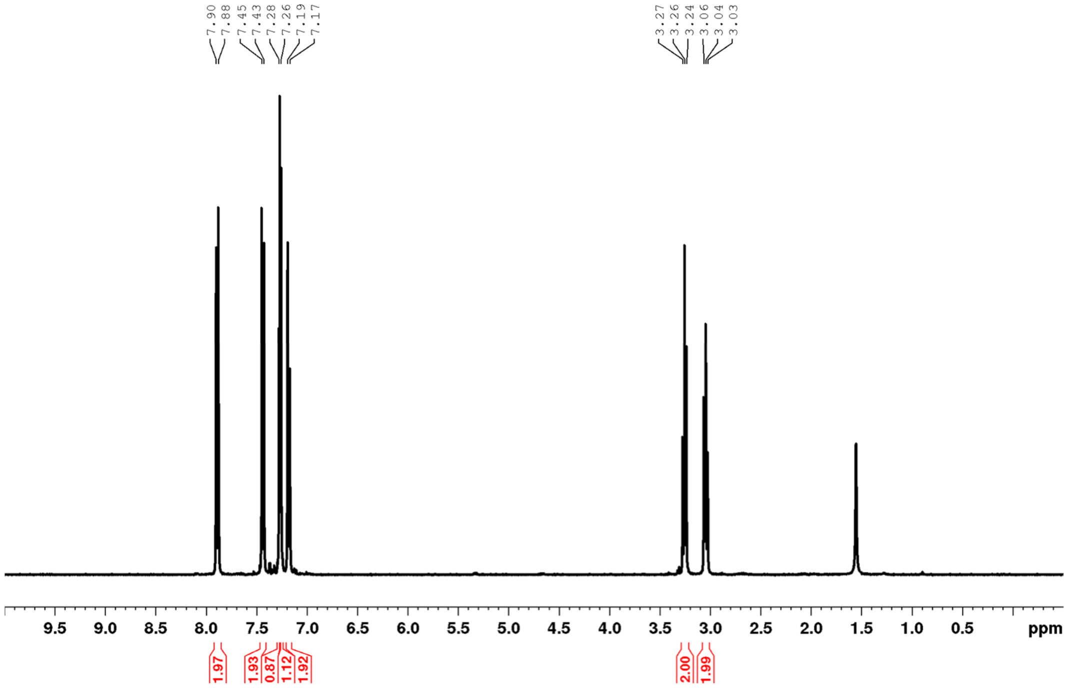

$^{13}C\{^{1}H\}$ NMR (101 MHz, $CDCl_3$)

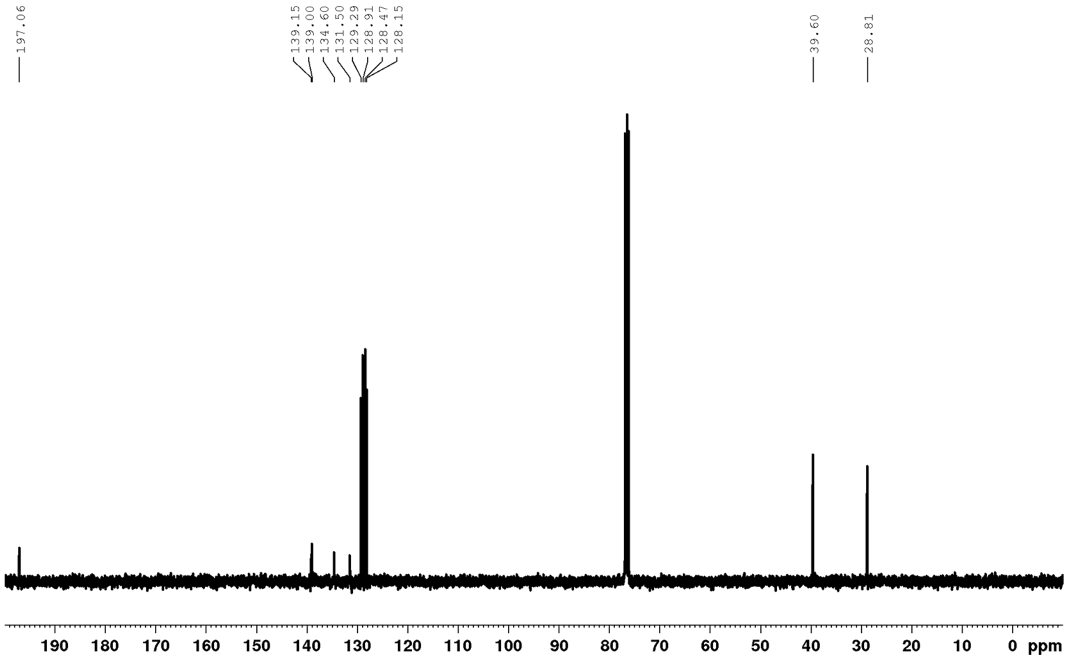

1-([1,1'-biphenyl]-4-yl)-3-(4-chlorophenyl)propan-1-one

^{1}H NMR (400 MHz, $CDCl_3$)

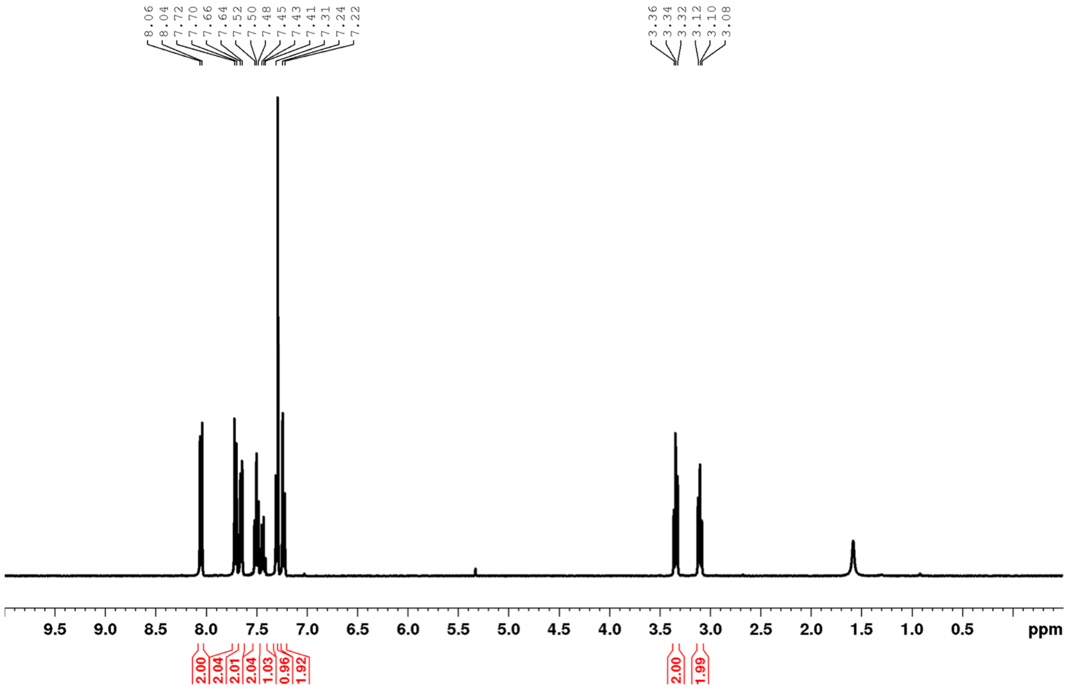

$^{13}C\{^1H\}$ NMR (101 MHz, $CDCl_3$)

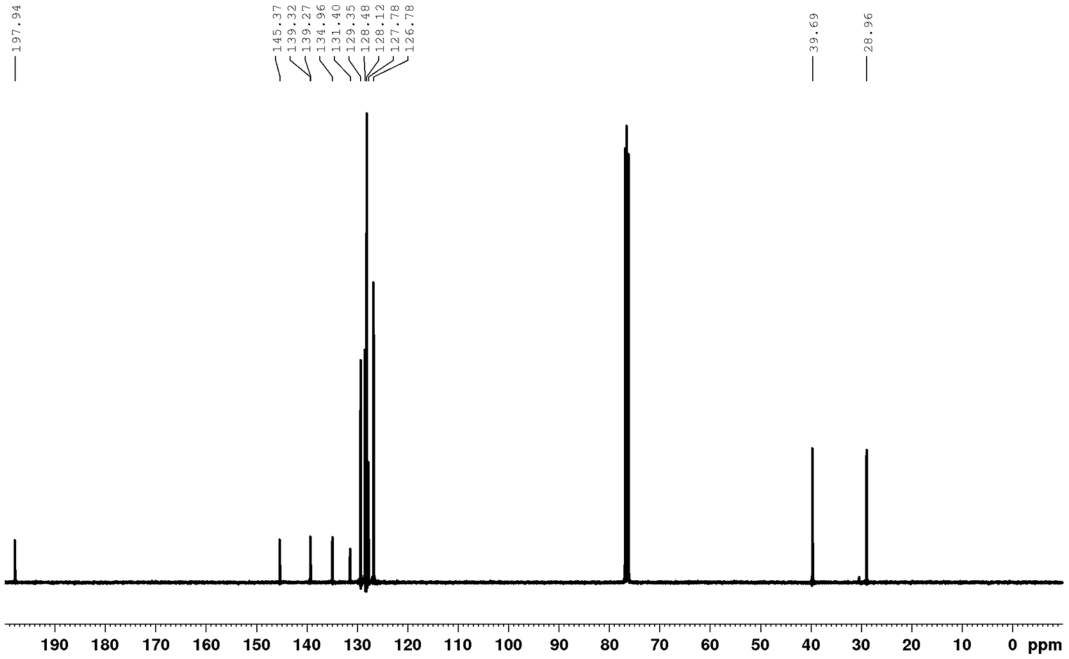

1,3-di([1,1'-biphenyl]-4-yl)propan-1-one

^{1}H NMR (400 MHz, $CDCl_3$)

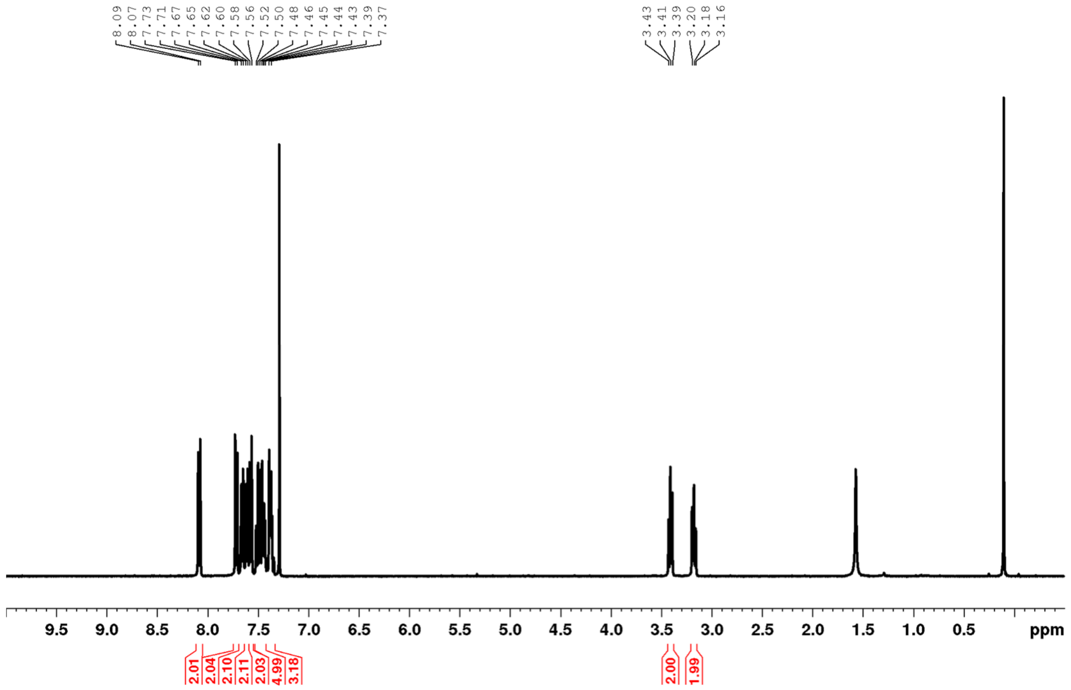

$^{13}C\{^1H\}$ NMR (101 MHz, $CDCl_3$)

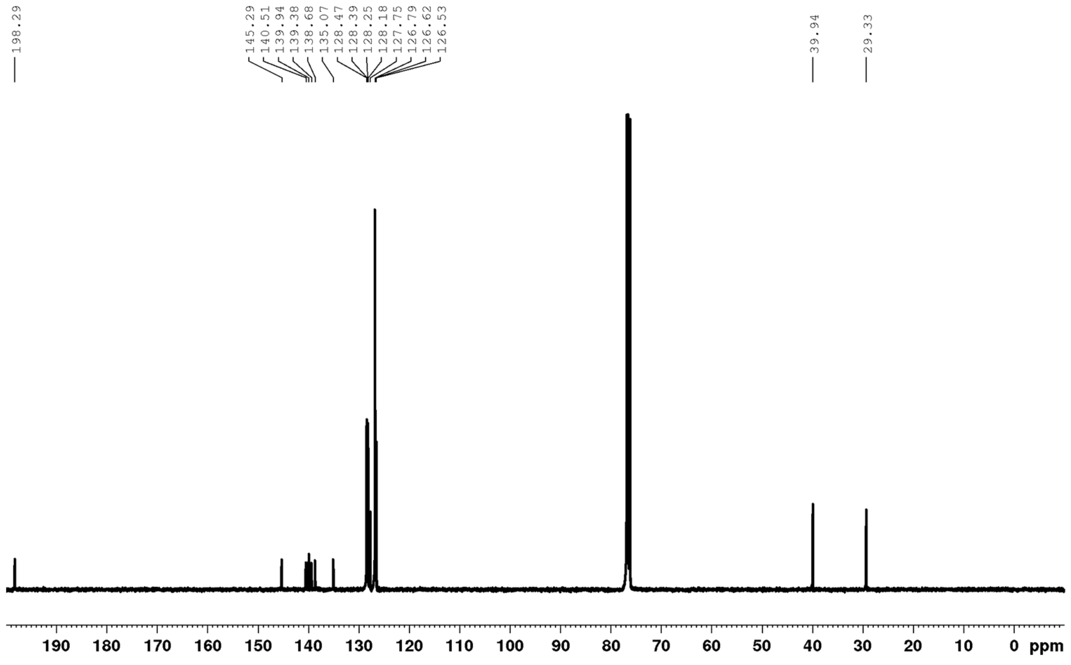

3-methoxy-4'-methyl-1,1'-biphenyl

^1H NMR (400 MHz, $CDCl_3$)

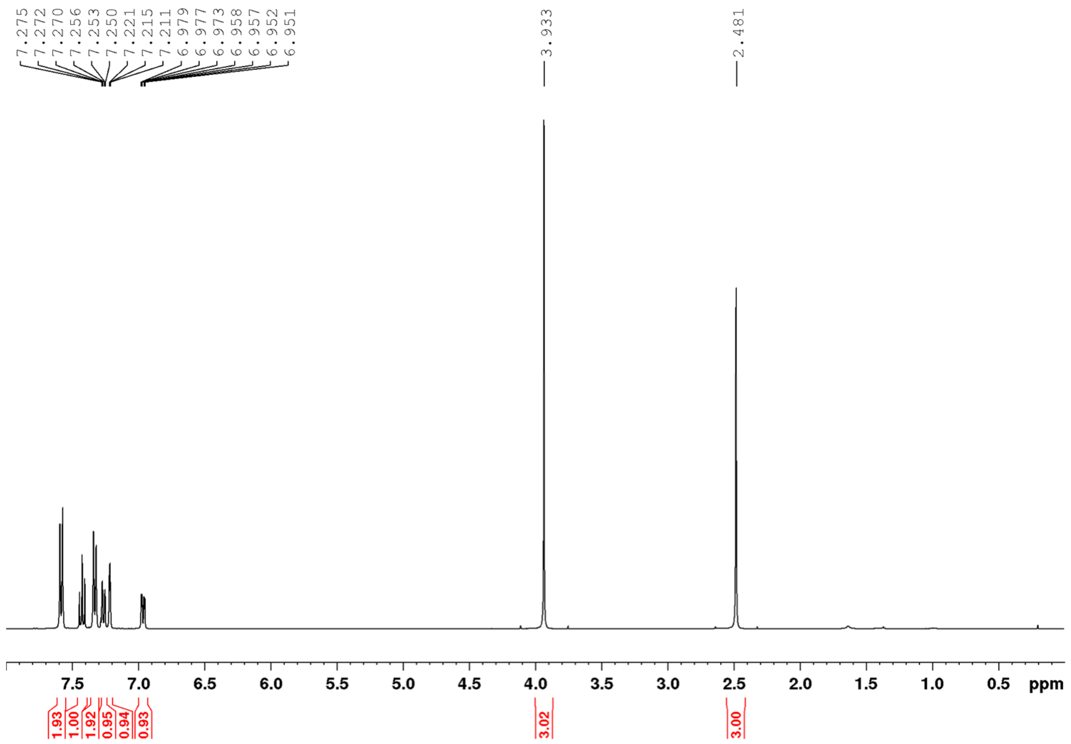

$^{13}C\{^1H\}$ NMR (101 MHz, $CDCl_3$)

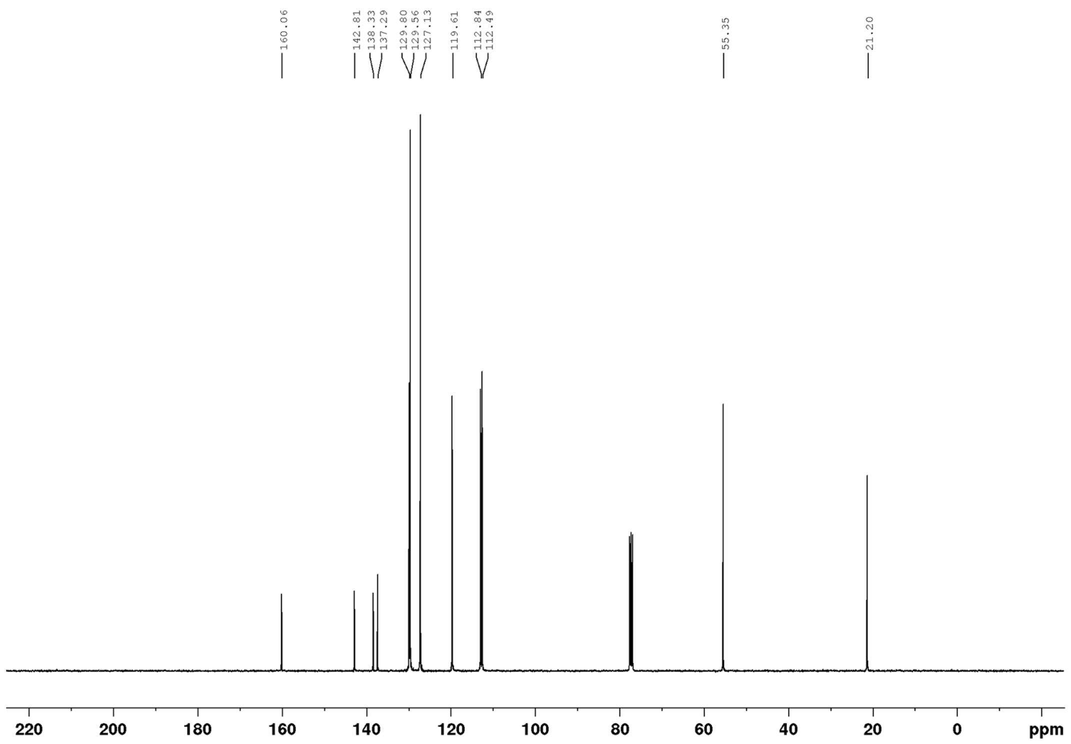

4-methyl-3-(trifluoromethyl)-1,1′-biphenyl

^{1}H NMR (400 MHz, $CDCl_3$)

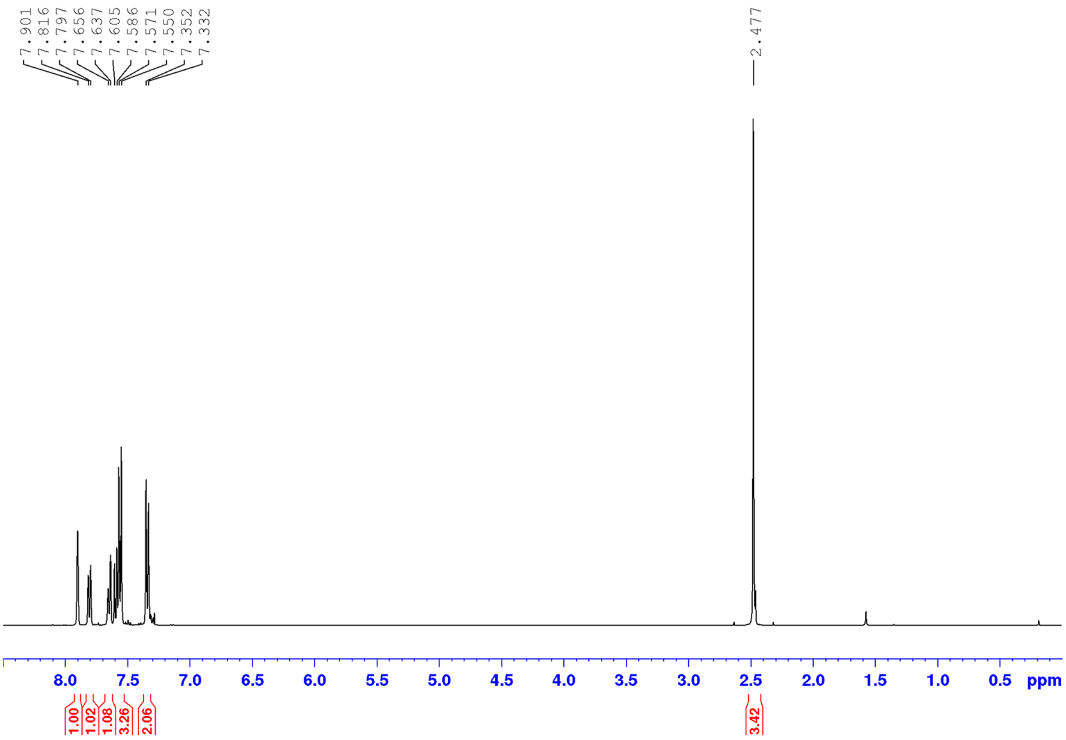

$^{13}C\{^{1}H\}$ NMR (101 MHz, $CDCl_3$)

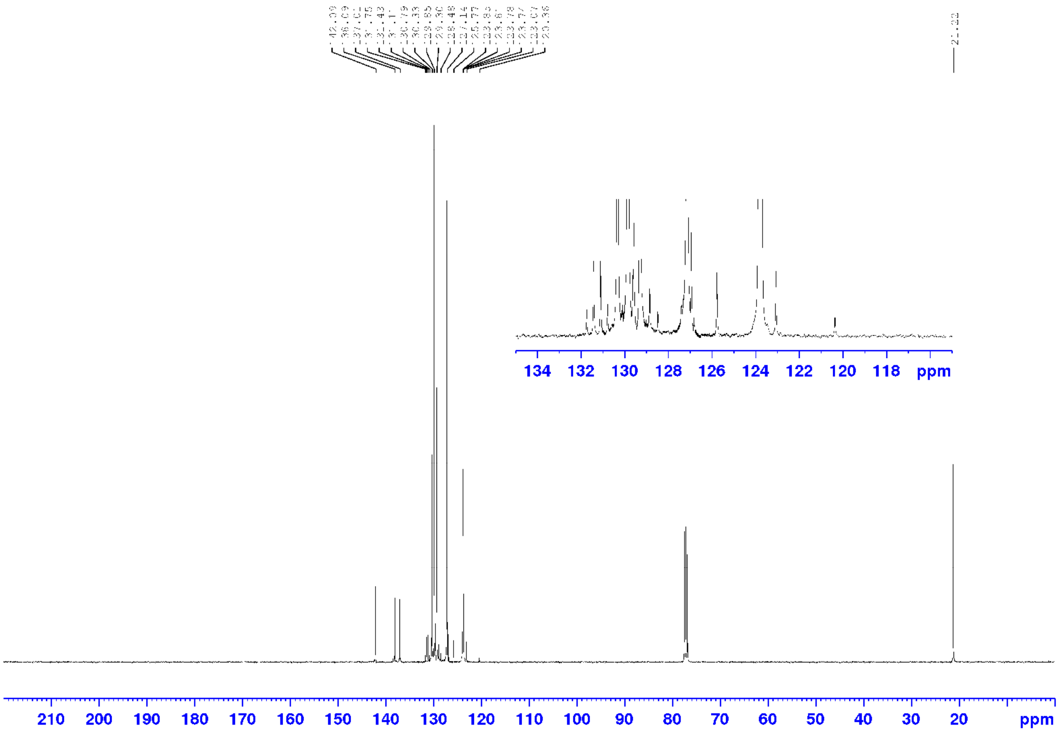

1-(4'-methyl-[1,1'-biphenyl]-3-yl)ethan-1-one

^{1}H NMR (400 MHz, $CDCl_3$)

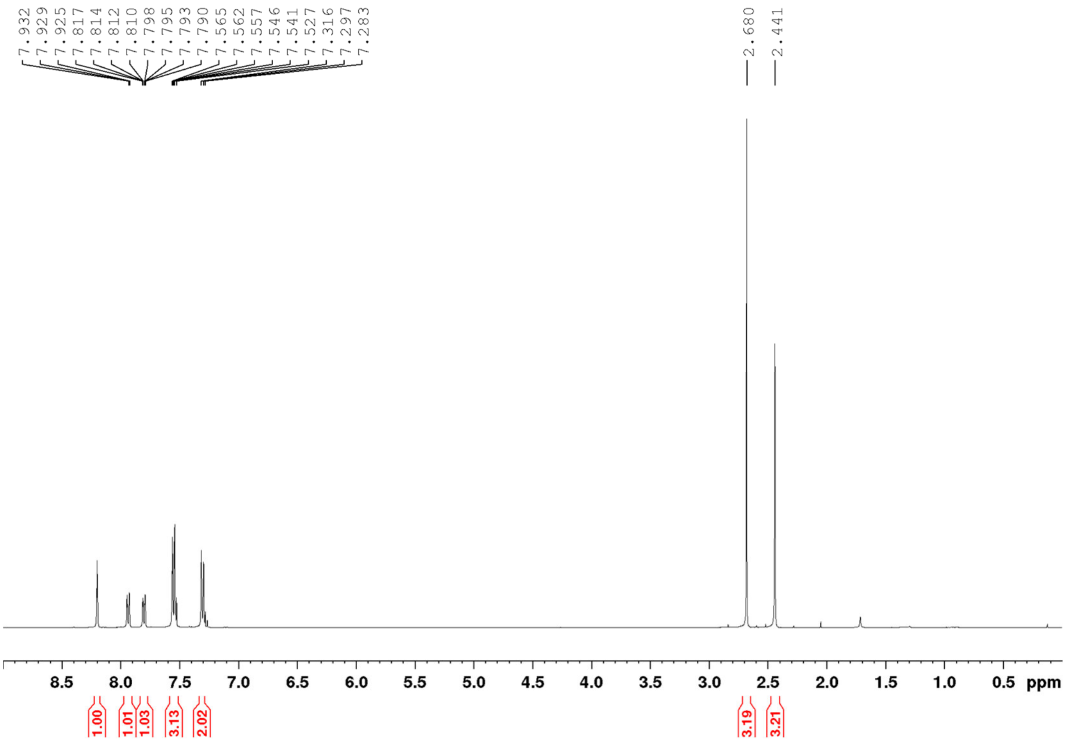

^{13}C{^{1}H} NMR (101 MHz, $CDCl_3$)

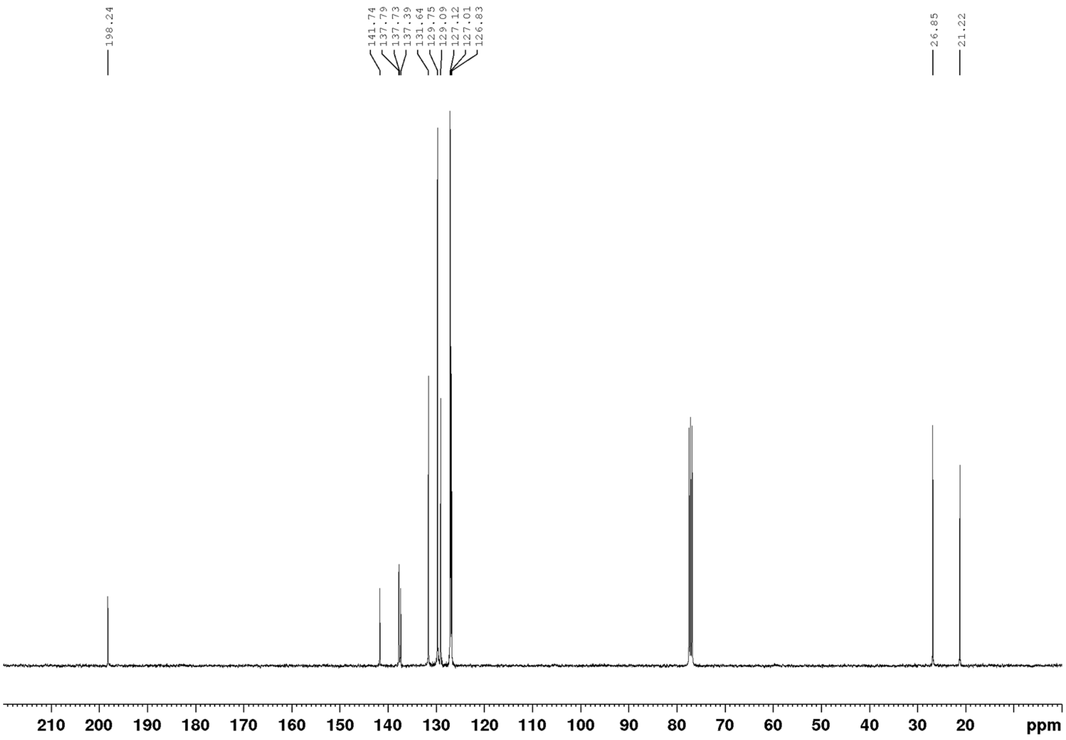

REFERENCES

1. G. Yin, I. Kalvet, U. Englert and F. Schoenebeck, *J. Am. Chem. Soc.*, 2015, **137**, 4164-4172.
2. E. A. Standley, S. J. Smith, P. Müller and T. F. Jamison, *Organometallics*, 2014, **33**, 2012-2018.
3. G. R. Fulmer, A. J. M. Miller, N. H. Sherden, H. E. Gottlieb, A. Nudelman, B. M. Stoltz, J. E. Bercaw and K. I. Goldberg, *Organometallics*, 2010, **29**, 2176-2179.
4. C. Fricke, G. J. Sherborne, I. Funes-Ardoiz, E. Senol, S. Guven and F. Schoenebeck, *Angew. Chem. Int. Ed.*, 2019, **58**, 17788-17795.
5. F. Kloss, T. Neuwirth, V. G. Haensch and C. Hertweck, *Angew. Chem. Int. Ed.*, 2018, **57**, 14476-14481.
6. R. Ambre, H. Yang, W.-C. Chen, G. P. A. Yap, T. Jurca and T.-G. Ong, *Eur. J. Inorg. Chem.*, 2019, **2019**, 3511-3517.
7. S. Kamio, I. Kageyuki, I. Osaka and H. Yoshida, *Chem. Commun.*, 2019, **55**, 2624-2627.
8. F. Serpier, F. Pan, W. S. Ham, J. Jacq, C. Genicot and T. Ritter, *Angew. Chem. Int. Ed.*, 2018, **57**, 10697-10701.
9. K. L. Wilson, J. Murray, C. Jamieson and A. J. B. Watson, *Synlett*, 2018, **29**, 650-654.
10. T. Yamada, K. Saito and T. Akiyama, *Adv. Synth. Catal.*, 2016, **358**, 62-66.
11. M. Bandini, R. Sinisi and A. Umani-Ronchi, *Chem. Commun.*, 2008, DOI: 10.1039/B807640E, 4360-4362.
12. J. Tang, A. Biafora and L. J. Goossen, *Angew. Chem. Int. Ed.*, 2015, **54**, 13130-13133.
13. Z. Luo, L. Xiong, T. Liu, Y. Zhang, S. Lu, Y. Chen, W. Guo, Y. Zhu and Z. Zeng, *J. Org. Chem.*, 2019, **84**, 10559-10568.
14. J. W. Fyfe, N. J. Fazakerley and A. J. Watson, *Angew Chem Int Ed Engl*, 2017, **56**, 1249-1253.
15. Z.-Q. Zhu, J.-S. He, H.-J. Wang and Z.-Z. Huang, *J. Org. Chem.*, 2015, **80**, 9354-9359.
16. A. K. Cooper, P. M. Burton and D. J. Nelson, *Synthesis*, 2020, DOI: 10.1055/s-0039-1690045.
17. S. Bajo, G. Laidlaw, A. R. Kennedy, S. Sproules and D. J. Nelson, *Organometallics*, 2017, **36**, 1662-1672.
18. S. Grimme, J. Antony, S. Ehrlich and H. Krieg, *J. Chem. Phys.*, 2010, **132**, 154104-154119.
19. U. Ryde, R. A. Mata and S. Grimme, *Dalton Trans.*, 2011, **40**, 11176-11183.
20. N. Fey, B. M. Ridgway, J. Jover, C. L. McMullin and J. N. Harvey, *Dalton Trans.*, 2011, **40**, 11184-11191.
21. A. V. Marenich, C. J. Cramer and D. G. Truhlar, *J. Phys. Chem. B*, 2009, **113**, 6378-6396.
22. P. J. Hay and W. R. Wadt, *J. Chem. Phys.*, 1985, **82**, 299-310.
23. W. R. Wadt and P. J. Hay, *J. Chem. Phys.*, 1985, **82**, 284-298.
24. P. J. Hay and W. R. Wadt, *J. Chem. Phys.*, 1985, **82**, 270-283.
25. C. E. Check, T. O. Faust, J. M. Bailey, B. J. Wright, T. M. Gilbert and L. S. Sunderlin, *J. Phys. Chem. A*, 2001, **105**, 8111-8116.
